# Supplementary material for: Evolutionary and biomedical implications of sex differences in the primate brain transcriptome
Source: Cell Genom. 2024 Jun 27;4(7):100589. doi: 10.1016/j.xgen.2024.100589 (PMC11293591; doi:10.1016/j.xgen.2024.100589)
Supplement: Document S1. Figures S1–S19 [file mmc1.pdf]

Cell Genomics, Volume *4*

Supplemental information

Evolutionary and biomedical implications of sex differences in the primate brain transcriptome

Alex R. DeCasien, Kenneth L. Chiou, Camille Testard, Arianne Mercer, Josué E. Negrón-Del Valle, Samuel E. Bauman Surratt, Olga González, Michala K. Stock, Angelina V. Ruiz-Lambides, Melween I. Martínez, Cayo Biobank Research Unit, Susan C. Antón, Christopher S. Walker, Jérôme Sallet, Melissa A. Wilson, Lauren J.N. Brent, Michael J. Montague, Chet C. Sherwood, Michael L. Platt, James P. Higham, and Noah Snyder-Mackler

Supplementary Figures

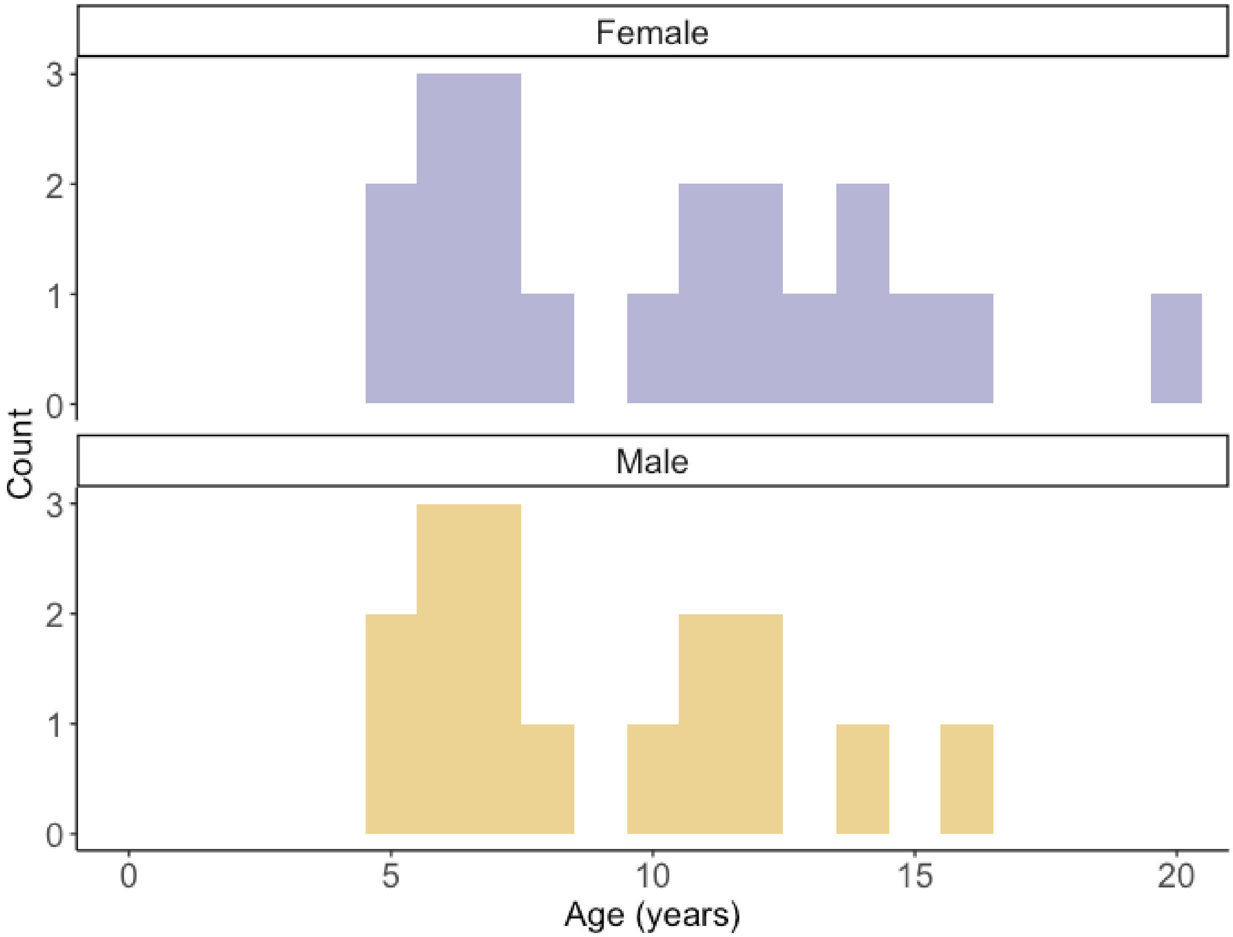

Figure S1 | Age distribution of adult male and female macaques included in this study (see Methods)

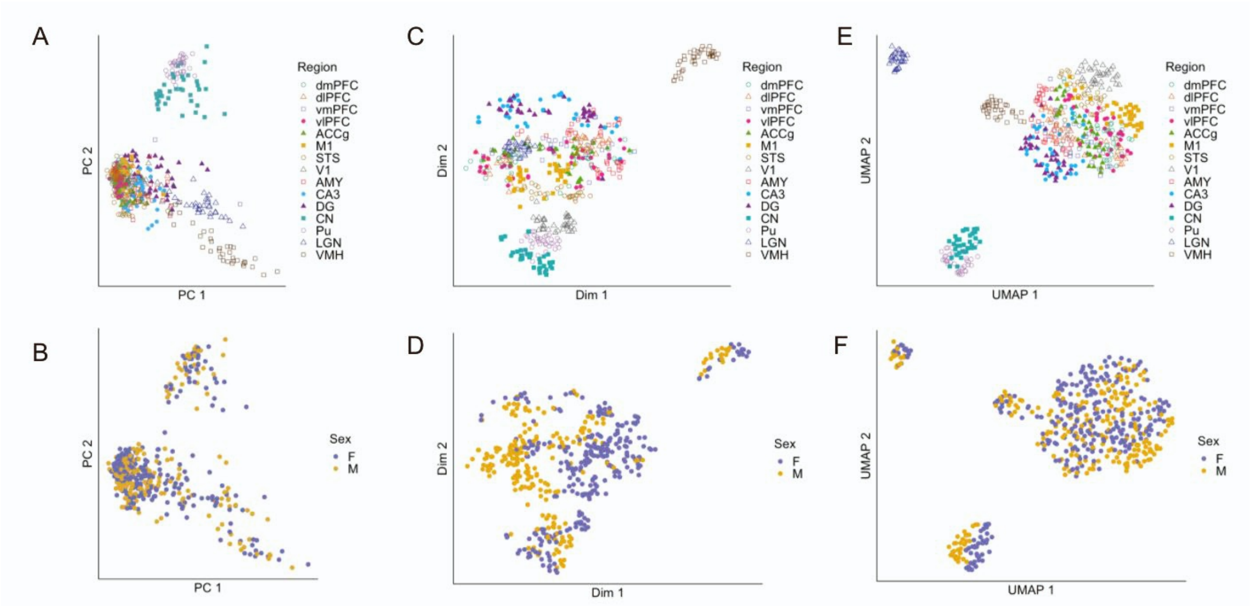

Figure S2 | Visualization of expression data obtained for study, related to Figure 1B.

A) Principal components analysis (PCA) plot of expression data. Each point represents one sample (N=527). Colors and shapes indicate region (see legend).

B) Principal components analysis (PCA) plot of expression data. Each point represents one sample (N=527). Colors indicate sex (see legend).

C) t-SNE plot of expression data. Each point represents one sample (N=527). Colors and shapes indicate region (see legend).

D) t-SNE plot of expression data. Each point represents one sample (N=527). Colors indicate sex (see legend).

E) Uniform Manifold Approximation and Projection (UMAP) plot of expression data. Each point represents one sample (N=527). Colors and shapes indicate region (see legend).

F) Uniform Manifold Approximation and Projection (UMAP) plot of expression data. Each point represents one sample (N=527). Colors indicate sex (see legend).

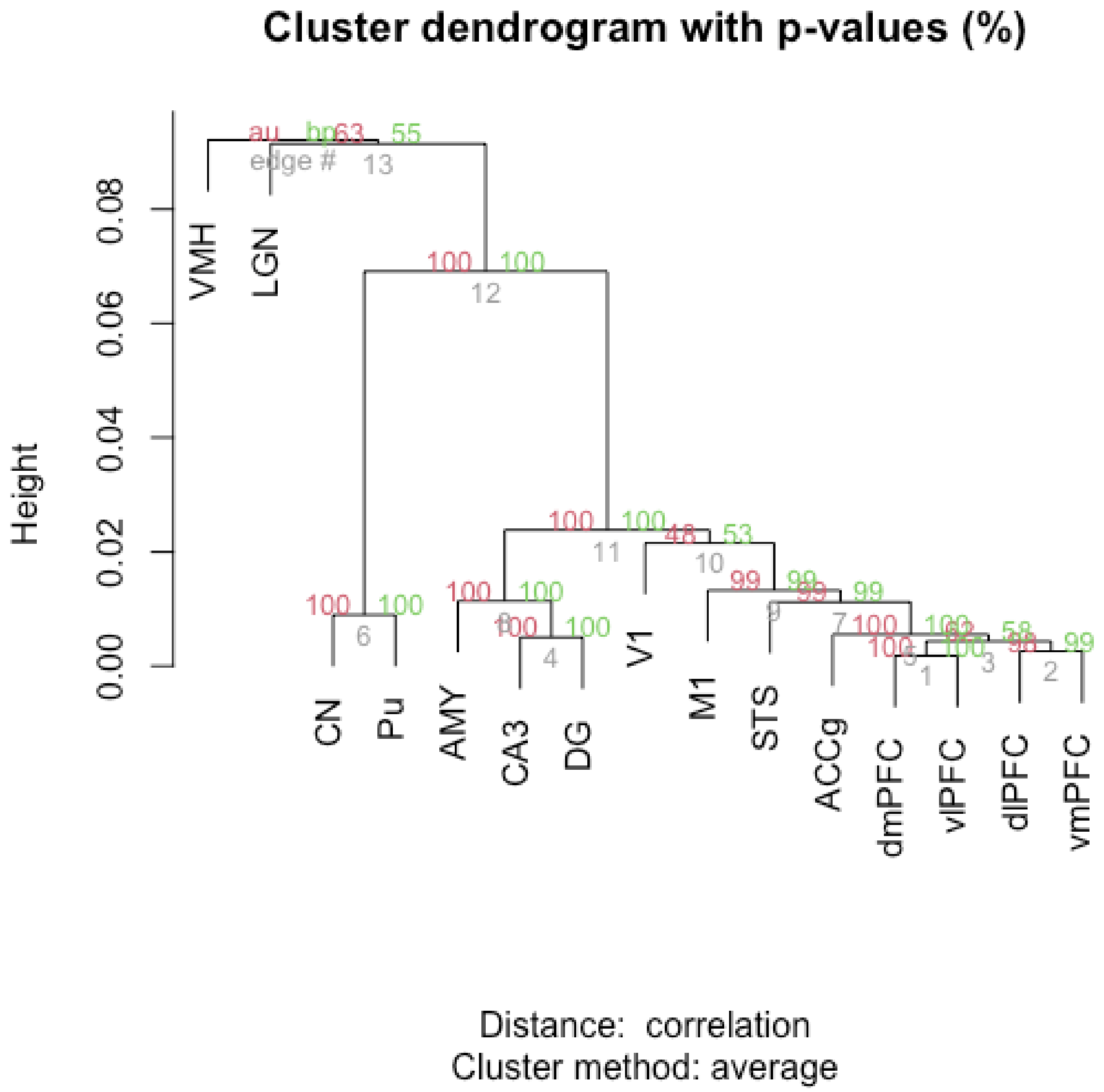

Figure S3 | Hierarchical clustering of brain regions, related to Figure 1B.

Dendrogram depicting hierarchical clustering of brain regions (using expression values averaged across samples within each region after removing batch effects). Approximately unbiased (AU) p-values (red) and bootstrap probability (BP) values (green) are depicted for each cluster. AU values are calculated using multiscale bootstrap resampling, while BP values are calculated by the ordinary bootstrap resampling.

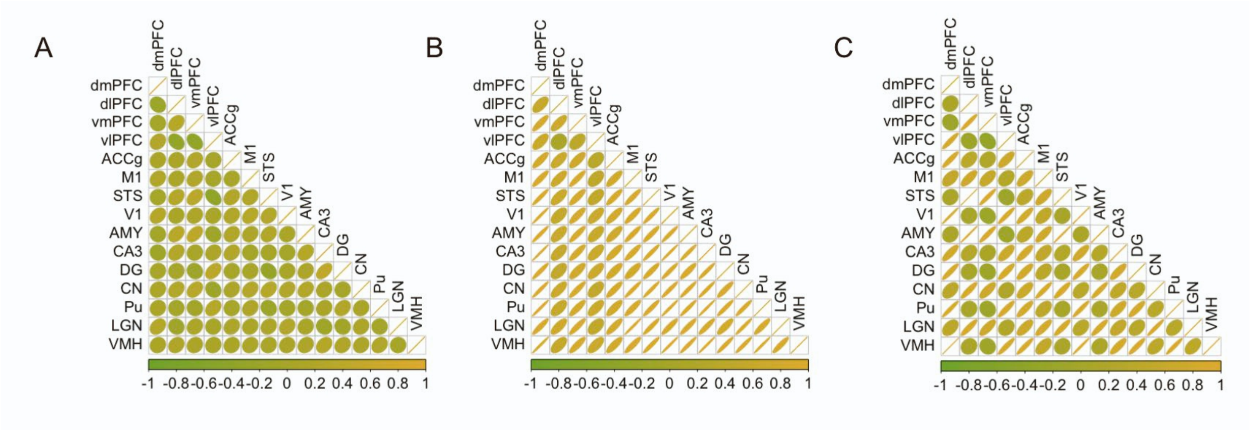

Figure S4 | Correlations of sex effects across regions, related to Figure 2A.

A) Correlations are shown for un-shrunken sex effect sizes (from EMMREML) using cell type corrected data across regions. Of these inter-regional correlations, 83 are significantly positive (p<0.05), 15 are significantly negative (p<0.05), and are 7 not significant (p>0.05). Yellow = positive correlation, green = negative correlation, circle = weak correlation; oval = stronger correlation in the given direction.

B) Correlations are shown for shrunken sex effect sizes (from MASHR) across regions. Of these inter-regional correlations, 105 are significantly positive (p<0.05). Yellow = positive correlation, green = negative correlation, circle = weak correlation; oval = stronger correlation in the given direction.

C) Correlations are shown for shrunken sex effect sizes (from MASHR) using cell type corrected data across regions. Of these inter-regional correlations, 96 are significantly positive (p<0.05) and 9 are significantly negative (p<0.05). Yellow = positive correlation, green = negative correlation, circle = weak correlation; oval = stronger correlation in the given direction.

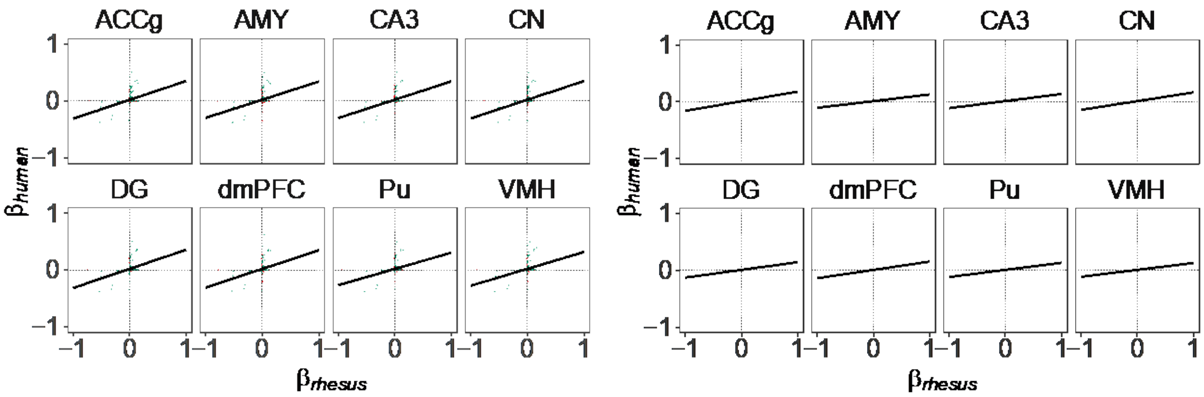

Figure S5 | Comparisons of sex effects in macaques vs. humans, related to Figure 3A.

Scatterplots of estimated sex effects for all one-to-one orthologous X chromosome genes (left) or autosomal genes (right). Green points represent genes with concordant sex-bias across species, while red points represent discordance.

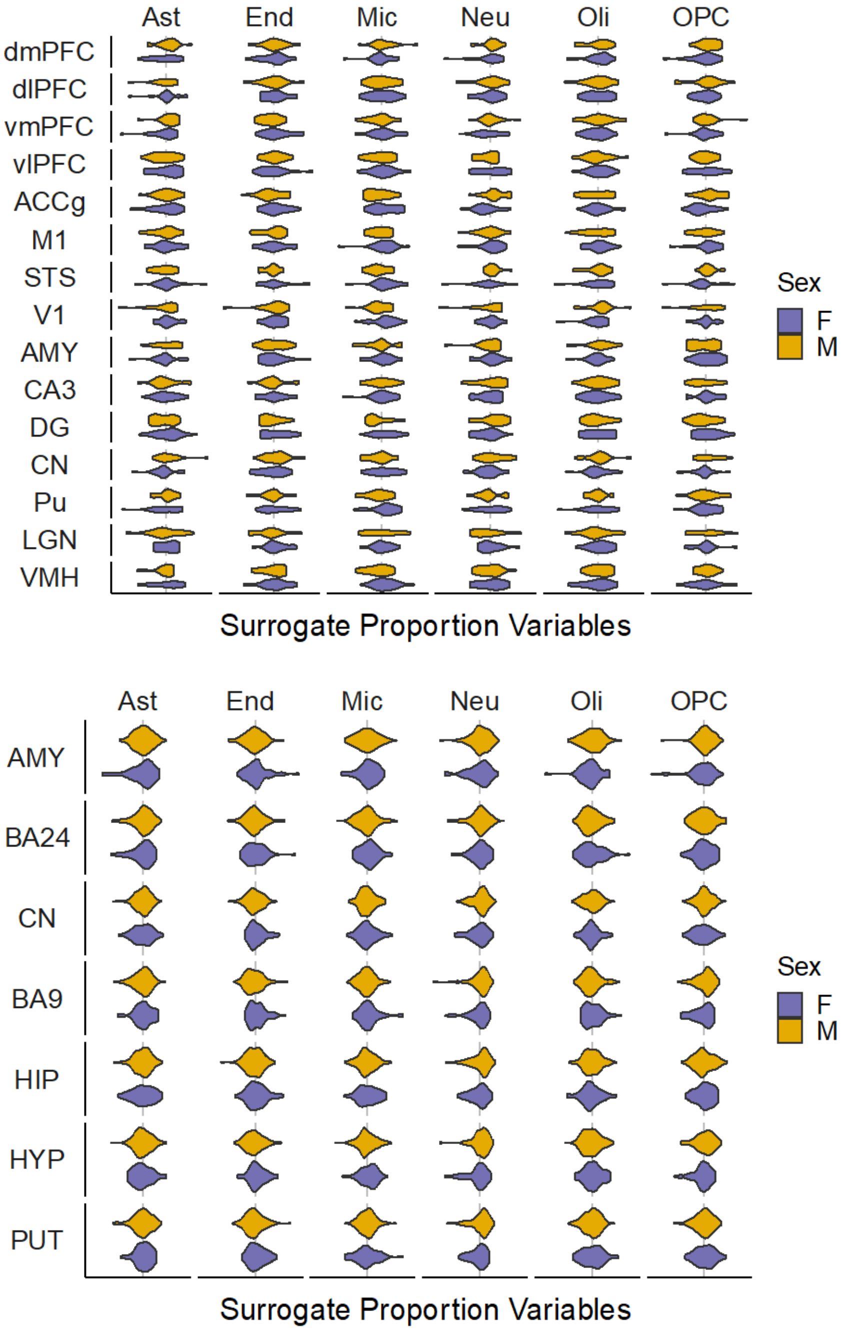

Figure S6 | Sex differences in estimated cell type proportions, related to Figure 3D.

Violin plots of estimated surrogate proportion variables (SPVs) estimated using BRETIGEA (see Methods) for macaques (top) and humans (bottom) within each region (y-axis). Vertical dashed grey line indicates SPV = 0. Ast = astrocytes; End = endothelial; Mic = microglia; Neu = neurons, Oli = oligodendrocytes; OPC = oligodendrocyte precursor cells.

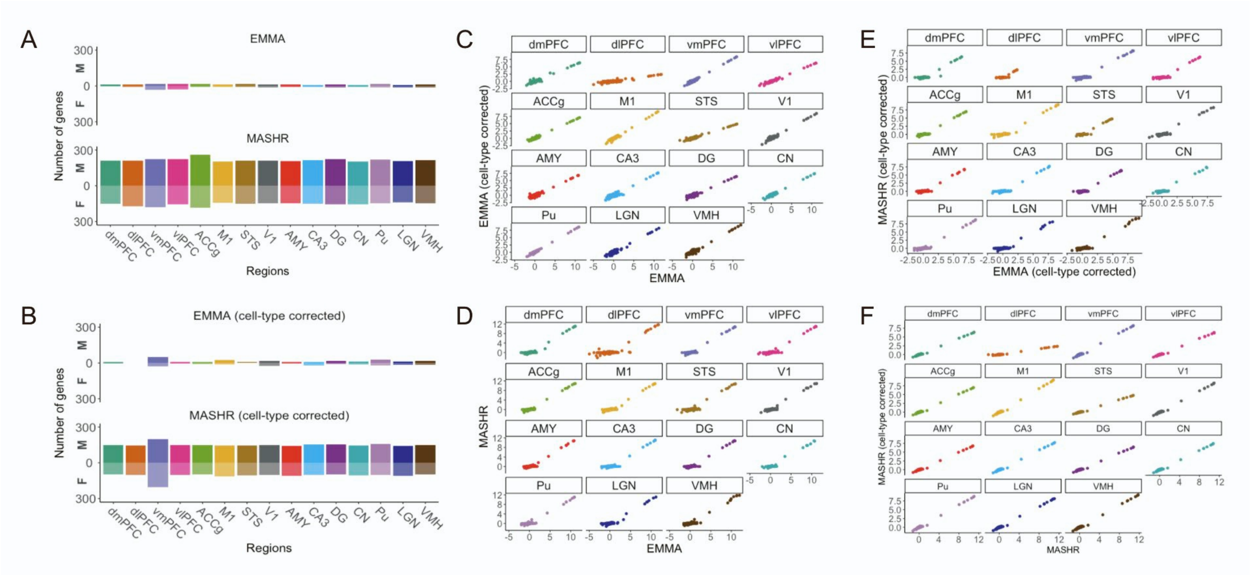

Figure S7 | Comparisons of unadjusted vs. cell type-adjusted analyses, related to Figures 4A-C.

A) Counts of sex-biased genes identified using EMMREML (top) and mashr (bottom) on unadjusted expression data. M = male-biased, F = female-biased.

B) Counts of sex-biased genes identified using EMMREML (top) and mashr (bottom) on cell-type adjusted expression data. M = male-biased, F = female-biased.

C) Sex effects (β) estimated for each gene within each region (x-axis: EMMREML, unadjusted expression data; y-axis: EMMREML, cell-type adjusted expression data) (positive values = male-biased; negative values = female-biased)

D) Sex effects (β) estimated for each gene within each region (x-axis: mashr, unadjusted expression data; y-axis: EMMREML, unadjusted expression data) (positive values = male-biased; negative values = female-biased)

E) Sex effects (β) estimated for each gene within each region (x-axis: EMMREML, cell-type adjusted expression data; y-axis: mashr, cell-type adjusted expression data) (positive values = male-biased; negative values = female-biased)

F) Sex effects (β) estimated for each gene within each region (x-axis: mashr, unadjusted expression data; y-axis: mashr, cell-type adjusted expression data) (positive values = male-biased; negative values = female-biased). Estimated sex effects tended to be in the same direction (i.e., male- or female-biased) whether cell type proportions were considered (74% concordance across all estimated effects; 99% concordance across effects that were significant in at least one analysis; $\rho = 0.635$, $p < 2.20e-16$).

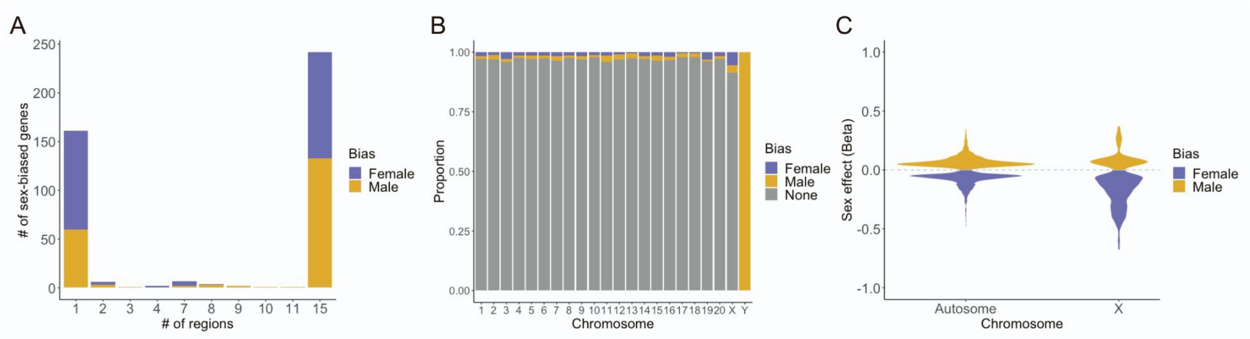

Figure S8 | Results for cell-type adjusted mashr analyses, related to Figures 4A-C.

A) Stacked bar chart of the number of sex-biased genes shared across different numbers of regions. 3.3% (422/12672) of brain expressed genes were differentially expressed in at least one region, and most of these genes exhibited sex-bias in a majority of regions (58.8% were biased in at least 8 tissues).

B) Proportions of genes on each chromosome that are not biased in any region (grey), female-biased in at least 1 region (purple), or male-biased in at least 1 region (yellow). Of the identified sex-biased genes, 9% were X-linked, 2% were Y-linked, and 89% were autosomal.

C) Violin plots of sex effect sizes for sex-biased autosomal versus X chromosome genes. Female-biased genes exhibited significantly larger (t-test: $p < 0.001$) magnitudes of their sex effects (mean $|\beta| = 0.10$) than male-biased genes (mean $|\beta| = 0.07$; excluding N = 9 Y chromosome genes, which are not expressed in females). In particular, female-biased X-linked genes exhibited significantly larger sex effects (N=25 genes; mean $|\beta| = 0.20$) than either male-biased X-linked genes (N = 14; mean $|\beta| = 0.10$; Tukey's HSD $p_{adj} < 0.001$) or sex-biased autosomal genes (female: N = 196, mean $|\beta| = 0.08$, $p_{adj} < 0.001$; male: N = 183: mean $|\beta| = 0.07$, $p_{adj} < 0.001$).

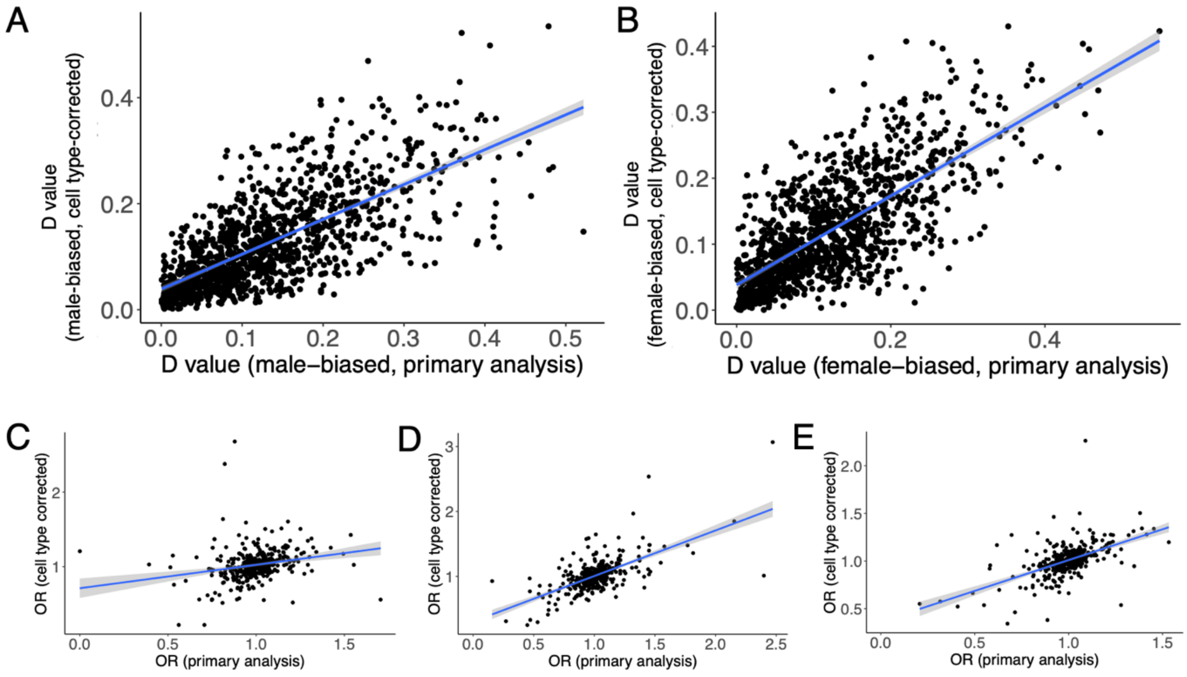

Figure S9 | Comparisons of macaque vs. human disease and motif enrichments, related to Figures 4D and 5.

A) D values for N=1257 diseases across primary and cell-type-corrected analyses (direction = male-biased; $\rho = 0.722$; $p < 2.2e\text{-}16$)

B) D values for N=1257 diseases across primary and cell-type-corrected analyses (direction = female-biased; $\rho = 0.704$; $p < 2.2e\text{-}16$)

C) ORs for N=414 motifs across primary and cell type-corrected analyses for male-biased genes ($\rho = 0.278$; $p = 3.048e\text{-}15$)

D) ORs for N=414 motifs across primary and cell type-corrected analyses for female-biased genes ($\rho = 0.542$; $p < 2.2e\text{-}16$)

E) ORs for N=414 motifs across primary and cell type-corrected analyses for sex-biased genes ($\rho = 0.552$; $p < 2.2e\text{-}16$)

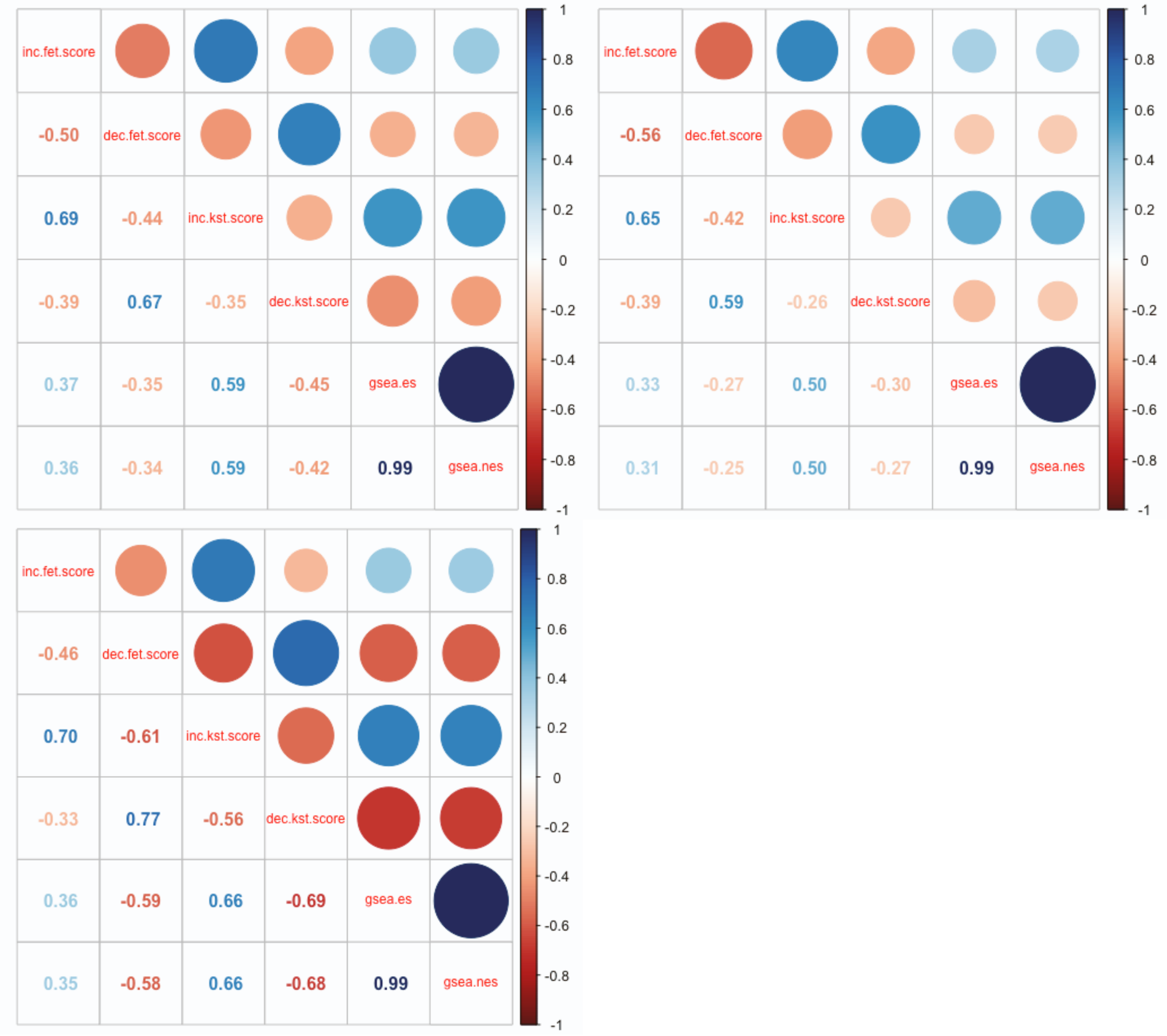

Figure S10 | Similarities across disease enrichment methods, related to Figure 5 (see Methods).

Correlations between disease enrichment scores across three approaches for macaque data (top left), macaque cell type-corrected data (top right), and human data (bottom). “inc.fet.score” = odds ratios (male-biased genes); “dec.fet.score” = odds ratios (female-biased genes); “inc.kst.score” = D-values (male-biased genes); “dec.kst.score” = D-values (female-biased genes); “gsea.es” = GSEA enrichment score (positive = male-biased, negative = female-biased); “gsea.nes” = GSEA normalized enrichment score (positive = male-biased, negative = female-biased). For the purposes of the correlation plot for the human data, diseases without enrichment scores (gsea.es = NA) were removed, and ORs = Inf were set to 15.

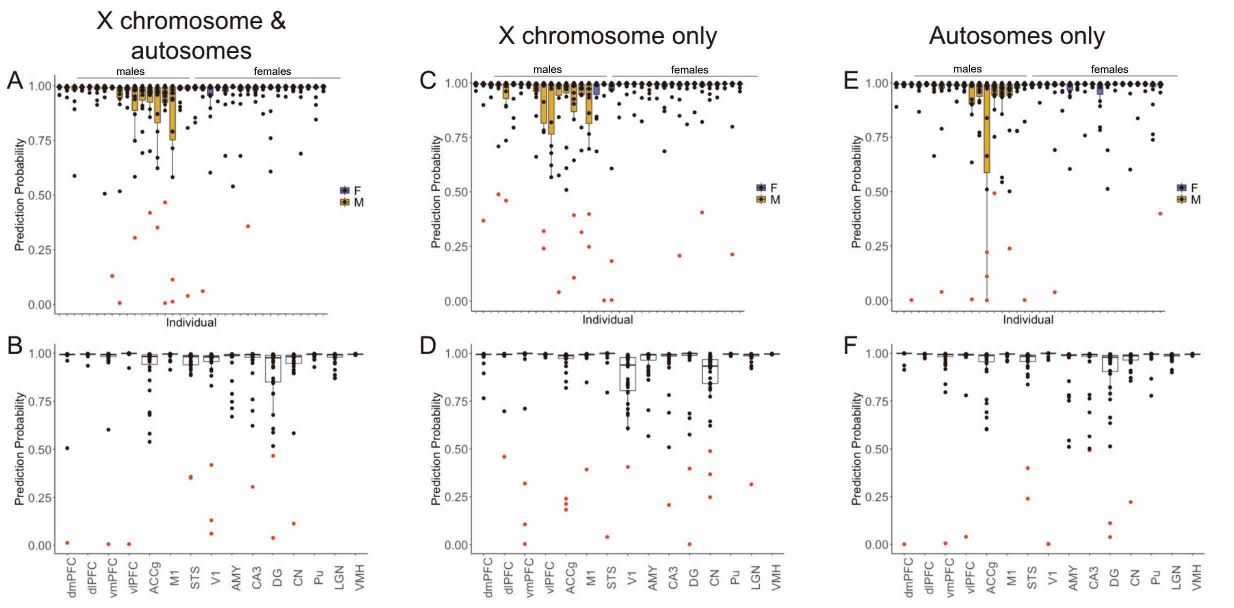

Figure S11 | Per sample sex prediction probabilities, related to Figure 6A.

A-F) Boxplots of prediction probabilities of the known sex per individual (top row) and region (bottom row). Dots indicate values for individual samples. Purple boxes = female, yellow boxes = male, black dots = correctly classified samples, red dots = incorrectly classified sample (prediction probability of correct sex < 0.5). Results are shown separately for models run incorporating all X chromosome and autosomal genes (A,B), X chromosome genes only (C,D), and autosomal genes only (E,F).

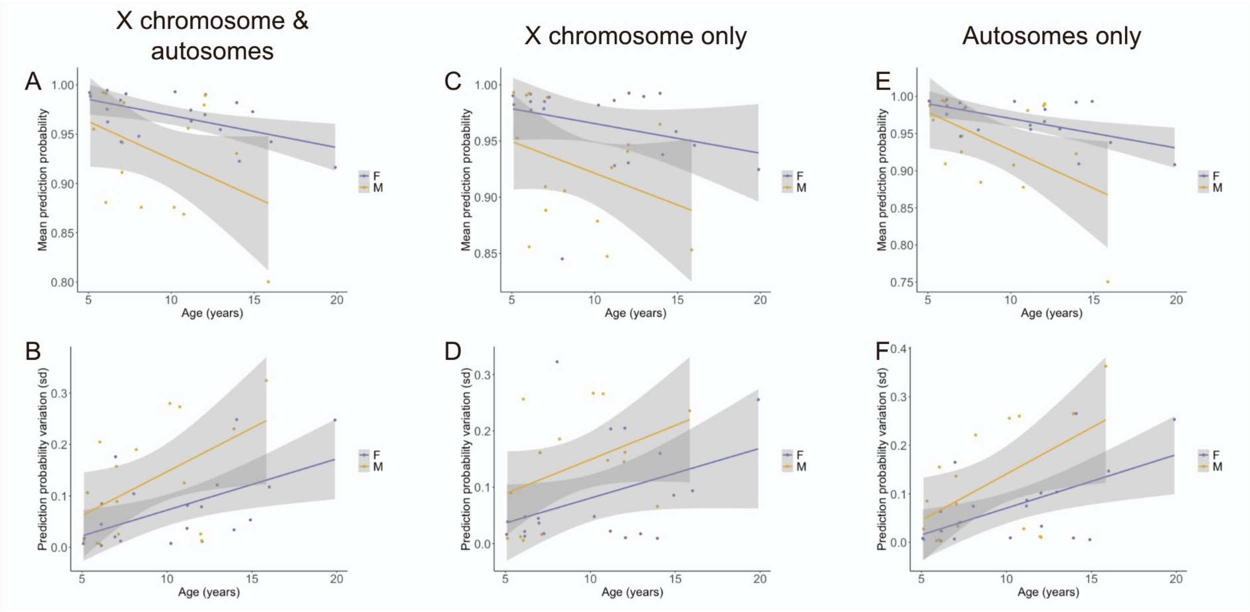

Figure S12 | Sex prediction probability (mean and sd) as a function of sex, related to Figure 6B.

A) Average prediction probability of known sex as a function of age (years) for females (purple) and males (yellow) or models run incorporating all X chromosome and autosomal genes (all: estimate = -0.003750, p = 0.0606; females: estimate = -0.003259, p = 0.00798; males: estimate = -0.007654, p = 0.0893)

B) Average standard deviation of prediction probability of known sex as a function of age (years) for females (purple) and males (yellow) or models run incorporating all X chromosome and autosomal genes (all: estimate = 0.010668, p = 0.0089; females: estimate = 0.009981, p = 0.0115; males: estimate = 0.01698, p = 0.0426)

C) Average prediction probability of known sex as a function of age (years) for females (purple) and males (yellow) or models run incorporating X chromosome genes only (all: estimate = -0.002656, p = 0.211; females: estimate = -0.002640, p = 0.202; males: estimate = -0.005653, p = 0.17)

D) Average standard deviation of prediction probability of known sex as a function of age (years) for females (purple) and males (yellow) or models run incorporating X chromosome genes only (all: estimate = 0.008390, p = 0.0469; females: estimate = 0.008853, p = 0.0853; males: estimate = 0.012116, p = 0.0983)

E) Average prediction probability of known sex as a function of age (years) for females (purple) and males (yellow) or models run incorporating autosomal genes only (all: estimate = -0.005151, p = 0.0158; females: estimate = -0.003971, p = 0.00482; males: estimate = -0.010242, p = 0.0356)

F) Average standard deviation of prediction probability of known sex as a function of age (years) for females (purple) and males (yellow) or models run incorporating autosomal genes only (all: estimate = 0.012182, p = 0.00411; females: estimate = 0.011033, p = 0.00782; males: estimate = 0.019074, p = 0.0315)

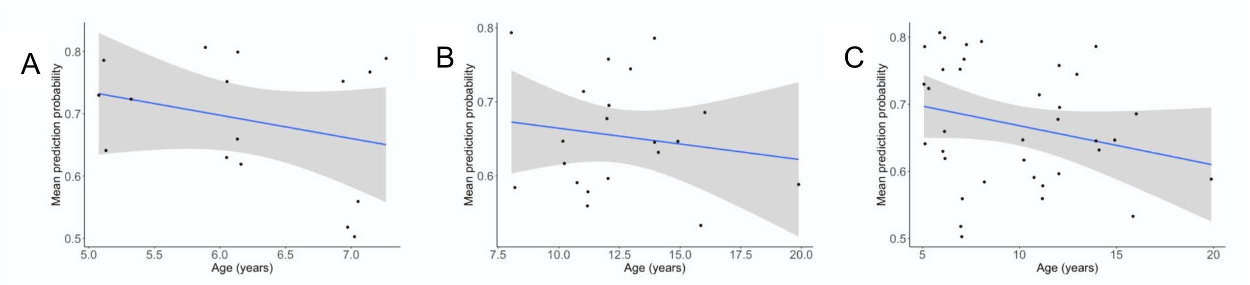

Figure S13 | Sex prediction probability (mean and standard deviation) as a function of sex, related to Figure 6B.

A) Prediction probability per individual (<=8 years group), averaged across regions and simulations (estimate = -0.037, p = 0.282).

B) Prediction probability per individual (>8 years group), averaged across regions and simulations (estimate = -0.004, p = 0.509).

C) Prediction probability per individual (combined results from separate analyses of both age groups), averaged across regions and simulations (estimate = -0.006, p = 0.139).

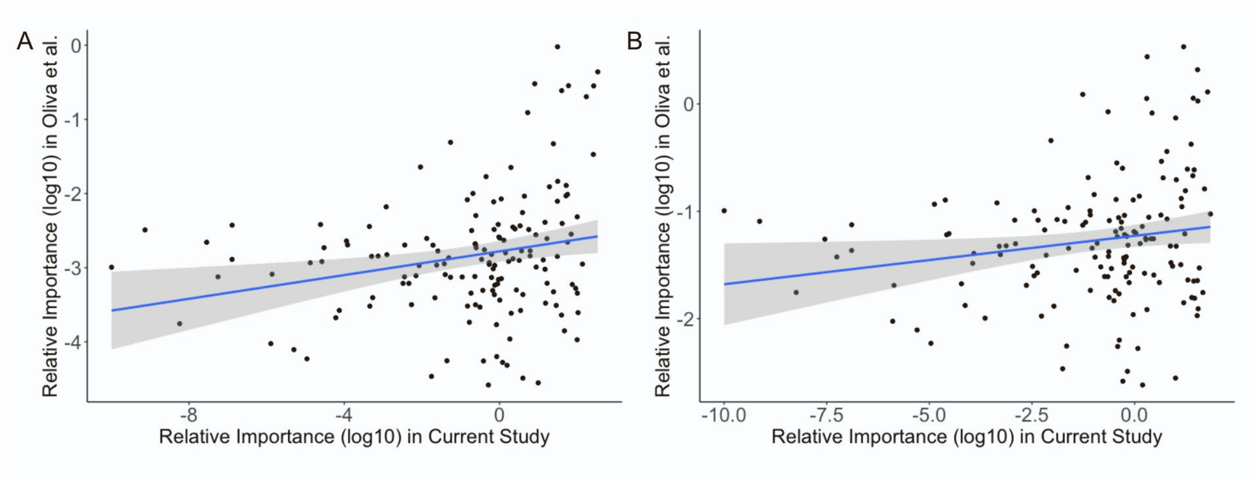

Figure S14 | Relative importance of X chromosome genes for sex prediction in macaques vs. humans, related to Figure 6D.

A) Relevance is summed across regions in both studies (ρ = 0.222, p = 0.006).

B) Relevance is averaged across regions in both studies (ρ = 0.0170, p = 0.038).

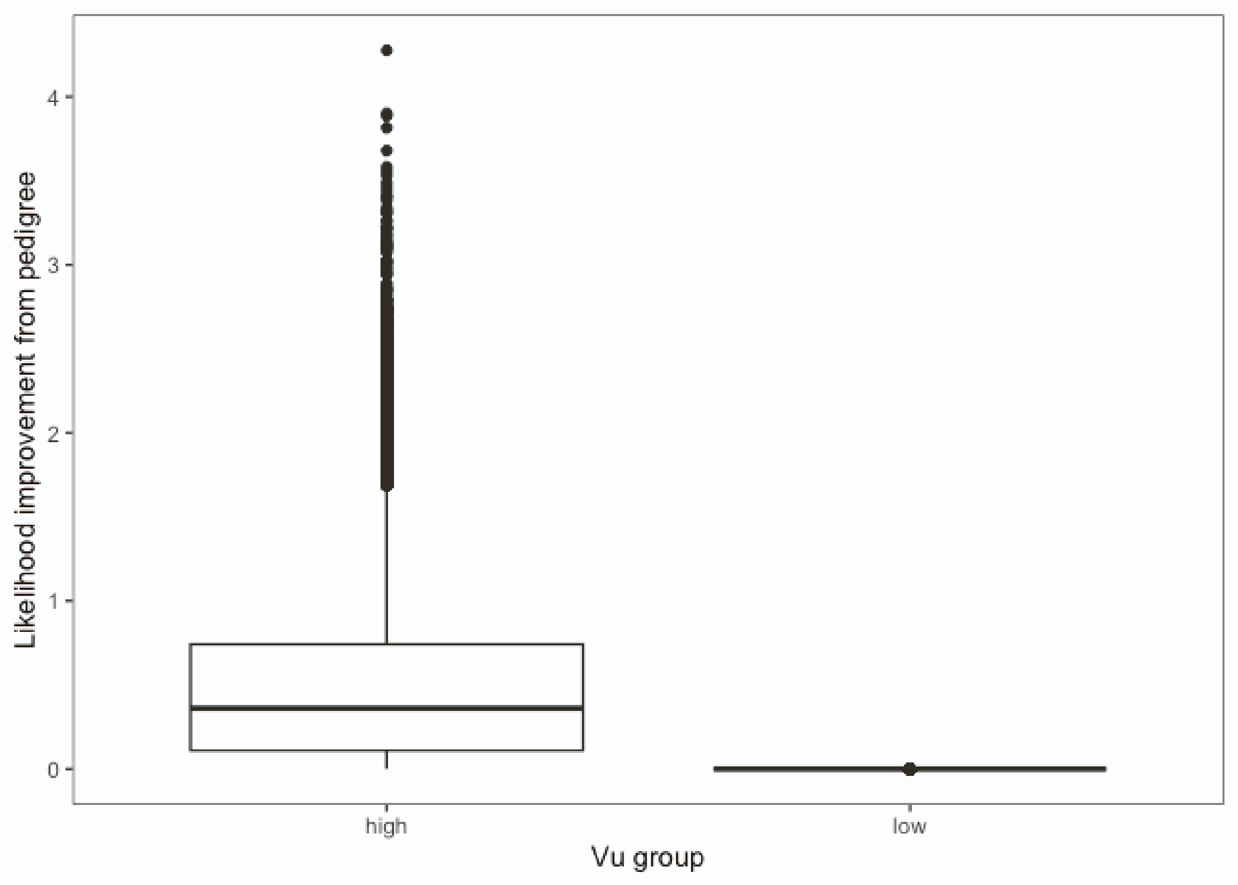

Figure S15 | Difference in likelihood between models that include the pedigree and those that do not, related to Figure 7B (see Methods).

“high *Vu*” group = log(*Vu*) > -15; “low *Vu*” group = log(*Vu*) < -15

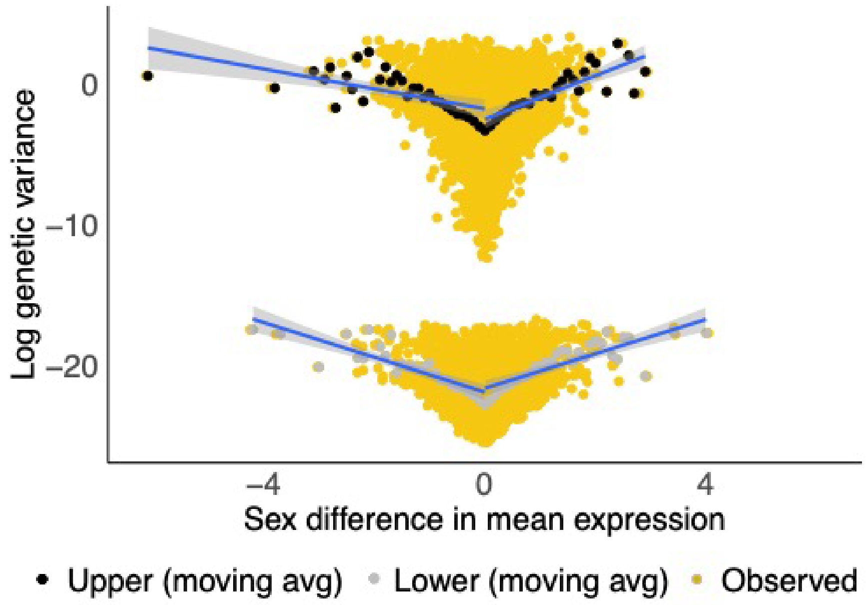

Figure S16 | Genetic variance as a function of sex-biased expression, related to Figure 7B.

Figure 4C with directional (instead of absolute) sex-bias (x-axis). Genetic variance (log) as a function of the difference in mean residual expression per gene and region (male-bias and upper *Vu*: ρ = 0.286; female-bias and upper *Vu*: -0.248; male-bias and lower *Vu*: ρ = 0. 315; female-bias and lower *Vu*: -0. 290; all p-values < 2.2e-16).

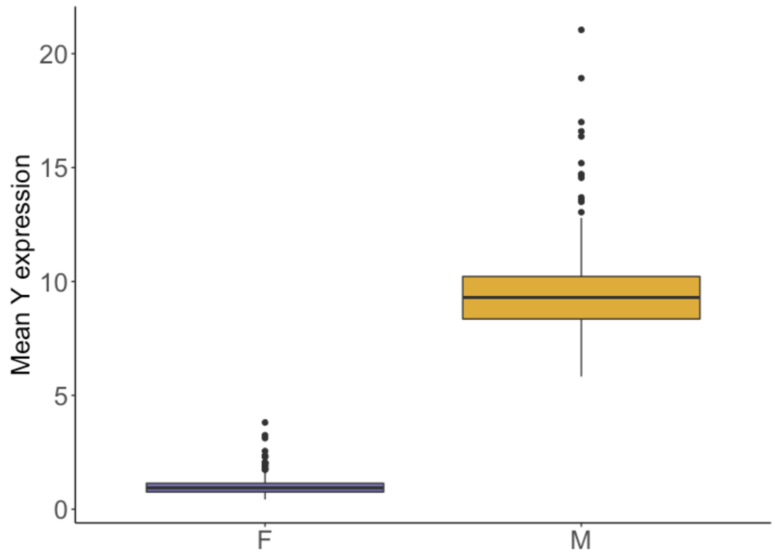

Figure S17 | Confirming chromosomal sex of samples using Y chromosome gene expression (see Methods).

Mean expression (TPM) of all Y genes for female and male samples. Reads were mapped to the original (non-sex specific) transcriptome. The female reads detected here represent the gametologues (X-Y homologues) and are not present in the sex-specific mapped data used in the manuscript. Boxplots indicate the median (black horizontal line), first and third quartiles (i.e., interquartile range, IQR; lower and upper hinges), and ranges extending from each to 1.5 x IQR beyond each hinge (whiskers). Points represent individual genes that are outliers (i.e., beyond whiskers).

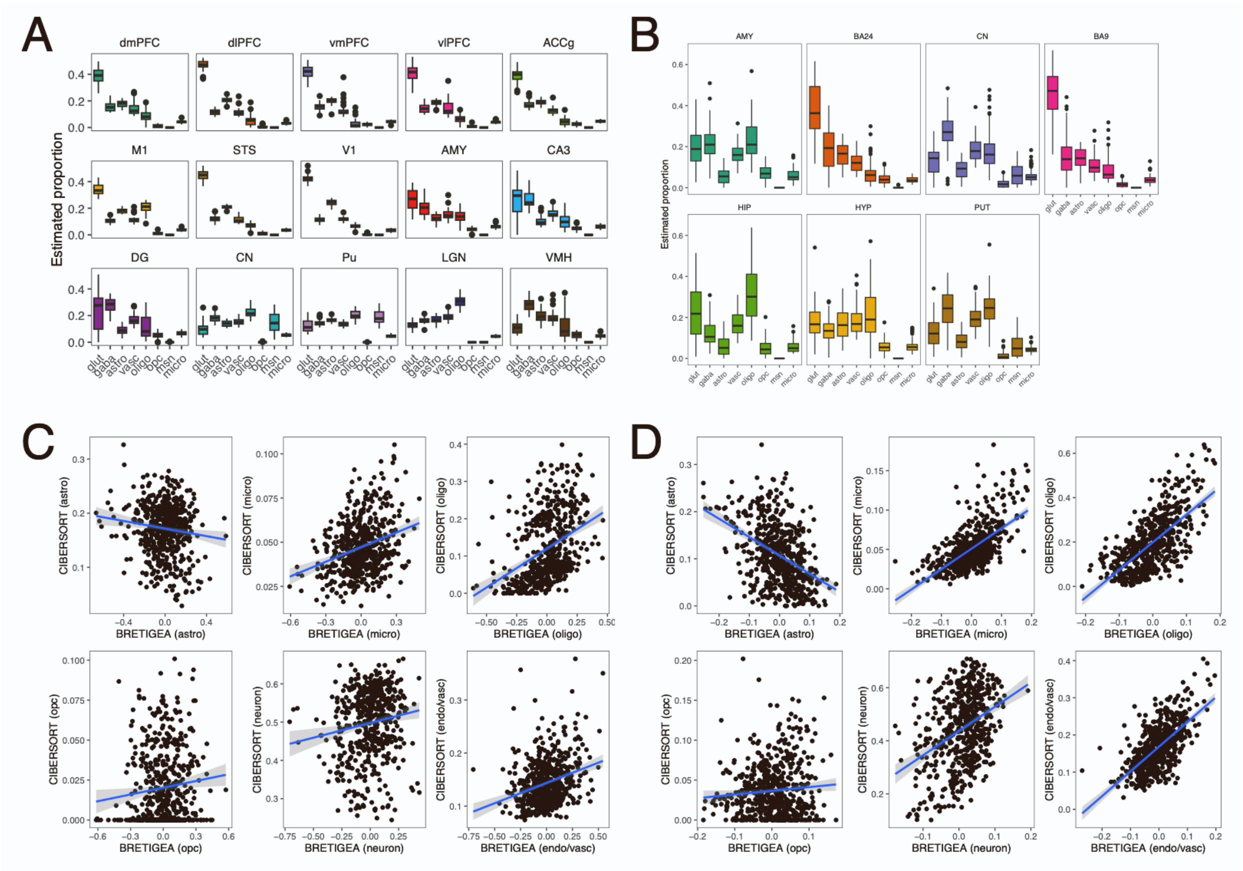

Figure S18 | Estimated cell type proportions across two methods, related to Figure 4A-C (see Methods).

A) CIBERSORT proportions in macaques for each region.

B) CIBERSORT proportions in humans for each region.

C) Correlations between CIBERSORT cell type proportions and BRETIGEA surrogate proportion variables across all macaque samples (astro = astrocytes, ρ = -0.070, p = 0.106; micro = microglia, ρ = 0.251, p = 5.728e-09; oligo = oligodendrocytes, ρ = 0.455, p = < 2.2e-16; opc = OPCs, 0.135, p = 0.002; neuron = neurons in BRETIGEA and glutaminergic + GABAergic neurons in CIBERSORT, ρ = 0.166, p < 0.001; endo/vasc = endothelial in BRETIGEA and vascular in CIBERSORT, ρ = 0.280, p < 7.216e-11).

D) Correlations between CIBERSORT cell type proportions and BRETIGEA surrogate proportion variables across all human samples (astro = astrocytes, ρ = -0.399, p = < 2.2e-16; micro = microglia, ρ = 0.632, p < 2.2e-16; oligo = oligodendrocytes, ρ = 0.656 p = < 2.2e-16; opc = OPCs, ρ = 0.020, p = 0.641; neuron = neurons in BRETIGEA and glutaminergic + GABAergic neurons in CIBERSORT, ρ = 0.327, p = 1.982e-15; endo/vasc = endothelial in BRETIGEA and vascular in CIBERSORT, ρ = 0.626, p < 2.2e-16).

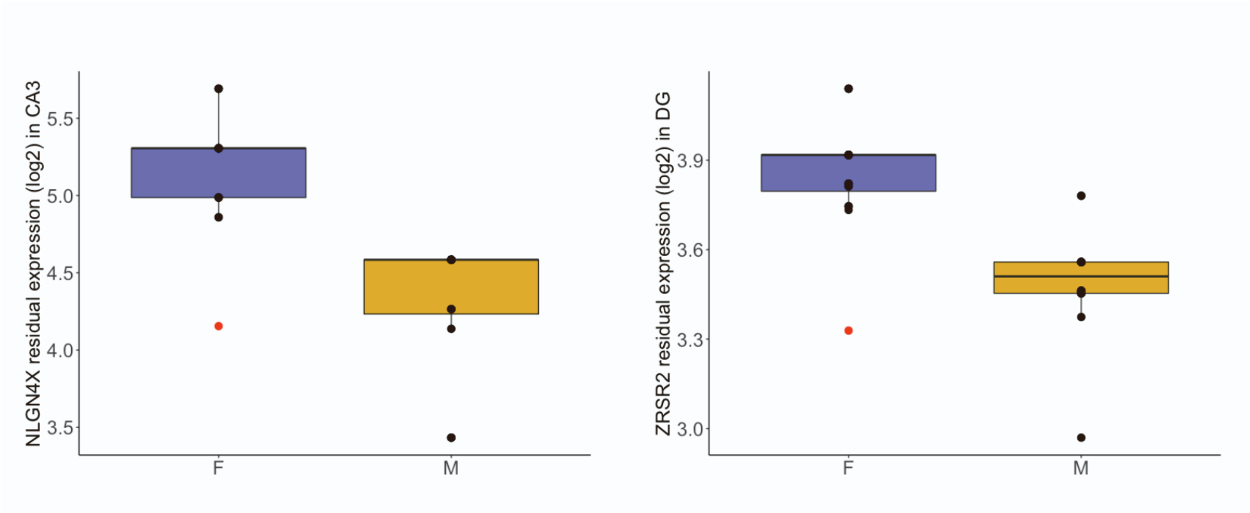

Figure S19 | Interindividual variation in expression of sex-predictive genes, related to Figure 6A (see Methods).

Boxplots of the residual expression values for the most influential gene in a given region from X chromosome gene models (left: *NLGN4X* in CA3; right: *ZRSR2* in DG). The red point represents the misclassified female sample. In humans, *ZRSR2* escapes and *NLGN4X* variably escapes XCI. Boxplots indicate the median (black horizontal line), first and third quartiles (i.e., interquartile range, IQR; lower and upper hinges), and ranges extending from each to 1.5 x IQR beyond each hinge (whiskers). Points represent individual genes that are outliers (i.e., beyond whiskers).
