# Supplementary material for: Evolutionary and biomedical implications of sex differences in the primate brain transcriptome
Source: Cell Genom. 2024 Jun 27;4(7):100589. doi: 10.1016/j.xgen.2024.100589 (PMC11293591; doi:10.1016/j.xgen.2024.100589)
Supplement: Document S2. Article plus supplemental information [file mmc28.pdf]

Cell Genomics

Article

Evolutionary and biomedical implications of sex differences in the primate brain transcriptome

Graphical abstract

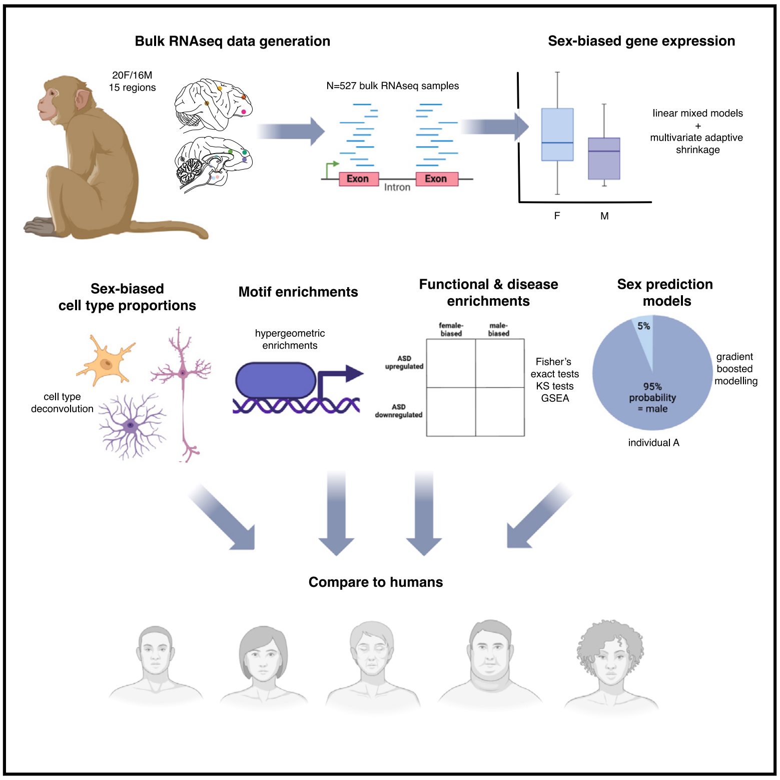

Authors

Alex R. DeCasien, Kenneth L. Chiou, Camille Testard, ..., Michael L. Platt, James P. Higham, Noah Snyder-Mackler

Correspondence

alex.decasien@gmail.com (A.R.D.),
kenny.chiou@gmail.com (K.L.C.),
jhigham@nyu.edu (J.P.H.),
nsnyderm@asu.edu (N.S.-M.)

In brief

DeCasien, Chiou, and colleagues demonstrate that human and rhesus macaque brains exhibit similar sex differences in gene expression and that these shared patterns overlap with genetic risk for a sex-biased condition (autism). These findings suggest that this model species may be appropriate for studying sex-biased human brain conditions.

Highlights

- We characterized sex-biased gene expression across 15 areas of the macaque brain
- Sex-biased gene expression partially reflects cell-type proportion/state differences
- Sex-biased genes are regulated by sex hormones
- Human and macaque brains show similar patterns with links to ASD

DeCasien et al., 2024, Cell Genomics 4, 100589
July 10, 2024 © 2024 The Authors. Published by Elsevier Inc.
https://doi.org/10.1016/j.xgen.2024.100589

CellPress

Cell Genomics

CellPress
OPEN ACCESS

Article

Evolutionary and biomedical implications of sex differences in the primate brain transcriptome

Alex R. DeCasien,[1,2,3,21,23,*] Kenneth L. Chiou,[4,5,6,7,21,*] Camille Testard,[8] Arianne Mercer,[6] Josué E. Negrón-Del Valle,[5] Samuel E. Bauman Surratt,[9] Olga González,[10] Michala K. Stock,[11] Angelina V. Ruiz-Lambides,[9] Melween I. Martínez,[9] Cayo Biobank Research Unit[12] Susan C. Antón,[1,2] Christopher S. Walker,[13] Jérôme Sallet,[14] Melissa A. Wilson,[4,5,15] Lauren J.N. Brent,[16] Michael J. Montague,[8] Chet C. Sherwood,[17] Michael L. Platt,[8,18,19] James P. Higham,[1,2,22,*] and Noah Snyder-Mackler[4,5,6,7,20,22,*]

[1]Department of Anthropology, New York University, New York, NY, USA
[2]New York Consortium in Evolutionary Primatology, New York, NY, USA
[3]Section on Developmental Neurogenomics, National Institute of Mental Health, Bethesda, MD, USA
[4]Center for Evolution and Medicine, Arizona State University, Tempe, AZ, USA
[5]School of Life Sciences, Arizona State University, Tempe, AZ, USA
[6]Department of Psychology, University of Washington, Seattle, WA, USA
[7]Nathan Shock Center of Excellence in the Basic Biology of Aging, University of Washington, Seattle, WA, USA
[8]Department of Neuroscience, University of Pennsylvania, Philadelphia, PA, USA
[9]Caribbean Primate Research Center, University of Puerto Rico, San Juan, PR, USA
[10]Southwest National Primate Research Center, Texas Biomedical Research Institute, San Antonio, TX, USA
[11]Department of Sociology and Anthropology, Metropolitan State University of Denver, Denver, CO, USA
[12]Department of Psychology, University of Pennsylvania, Philadelphia, PA 19104, USA
[13]Department of Molecular Biomedical Sciences, College of Veterinary Medicine, North Carolina State University, Raleigh, NC, USA
[14]Stem Cell and Brain Research Institute, Université Lyon, Lyon, France
[15]Biodesign Center for Mechanisms of Evolution, Arizona State University, Tempe, AZ, USA
[16]Centre for Research in Animal Behavior, University of Exeter, Exeter, UK
[17]Department of Anthropology, The George Washington University, Washington, DC, USA
[18]Department of Psychology, University of Pennsylvania, Philadelphia, PA, USA
[19]Department of Marketing, University of Pennsylvania, Philadelphia, PA, USA
[20]ASU-Banner Neurodegenerative Disease Research Center, Arizona State University, Tempe, AZ, USA
[21]These authors contributed equally
[22]These authors contributed equally
[23]Lead contact
*Correspondence: alex.decasien@gmail.com (A.R.D.), kenny.chiou@gmail.com (K.L.C.), jhigham@nyu.edu (J.P.H.), nsnyderm@asu.edu (N.S.-M.)
https://doi.org/10.1016/j.xgen.2024.100589

SUMMARY

Humans exhibit sex differences in the prevalence of many neurodevelopmental disorders and neurodegenerative diseases. Here, we generated one of the largest multi-brain-region bulk transcriptional datasets for the rhesus macaque and characterized sex-biased gene expression patterns to investigate the translatability of this species for sex-biased neurological conditions. We identify patterns similar to those in humans, which are associated with overlapping regulatory mechanisms, biological processes, and genes implicated in sex-biased human disorders, including autism. We also show that sex-biased genes exhibit greater genetic variance for expression and more tissue-specific expression patterns, which may facilitate rapid evolution of sex-biased genes. Our findings provide insights into the biological mechanisms underlying sex-biased disease and support the rhesus macaque model for the translational study of these conditions.

INTRODUCTION

Humans exhibit sex/gender differences in prevalence, presentation, and progression of many psychiatric, neurodevelopmental, and neurodegenerative conditions. For example, depression[1] and Alzheimer's disease (AD)[2] are more prevalent in females, whereas attention-deficit/hyperactivity disorder (ADHD),[3] autism spectrum disorders (ASDs),[4] and Parkinson's disease[5] occur more often in males. Although gender biases in the applicability of diagnostic criteria contribute to these differences,[6] neurobiological sex differences are likely to play a role since (1) multiple diagnostically distinct disorders show the same sex bias during the same developmental window (e.g., male-biased early-onset neurodevelopmental disorders), and (2) sex-biased disorders tend to emerge during dynamic neurodevelopmental periods that involve changes to sex hormone concentrations (e.g., adolescence, menopause).[7,8] Studies of postmortem human brains have highlighted

© 2024 The Authors. Published by Elsevier Inc.
This is an open access article under the CC BY license (http://creativecommons.org/licenses/by/4.0/).

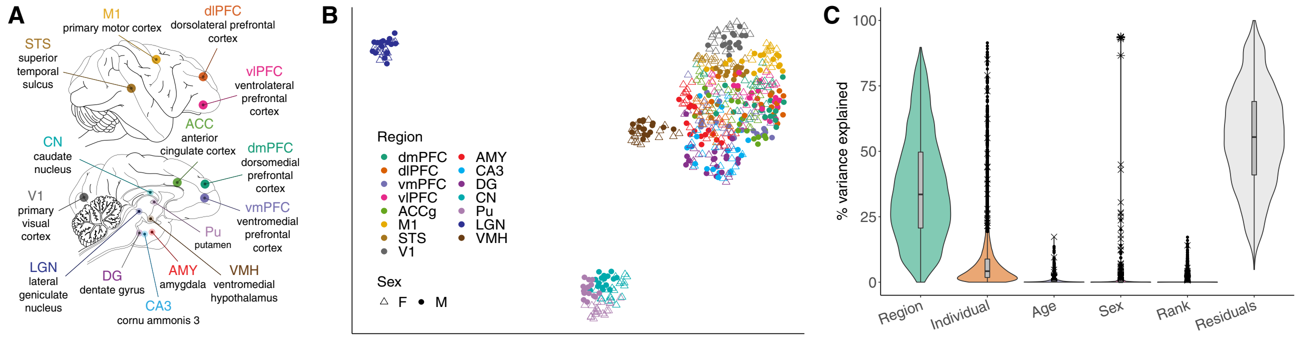

Figure 1. Experimental design and global expression patterns

(A) Fifteen brain regions sampled in the current study. Top = lateral view. Bottom = medial view. Some structures are internal and cannot be viewed from the planes depicted.

(B) Uniform manifold approximation and projection (UMAP) plot of expression data. Each point represents one sample (*n* = 527). Colors indicate region and shapes indicate sex (see legend).

(C) Violin plots with overlaid boxplots of variance proportions for each gene and variable from variance partitioning analysis. Boxplots indicate the median (black horizontal line), first and third quartiles (i.e., interquartile range [IQR]; lower and upper hinges), and ranges extending from each to 1.5 × IQR beyond each hinge (whiskers). Points represent individual genes that are outliers (i.e., beyond whiskers), and their shape indicates the chromosomal location (autosome = ●, X chromosome = ×, Y chromosome = *).

molecular mechanisms that may underlie such differences: many genes associated with sex-biased neurological conditions are also expressed at different levels in healthy male and female brains.[9–17]

However, our understanding of the proximate and evolutionary sources of normative transcriptomic sex differences in human brains is currently limited due to (1) a dearth of postmortem human brain samples, which tend to be heterogeneous in terms of co-occurring diseases and processing methods, and (2) the fact that most work on neurobiological sex differences has been conducted on laboratory rodents, which are distantly related to and neuroanatomically distinct from humans. Among existing animal models, rhesus macaques (*Macaca mulatta*) arguably have the greatest potential translatability to humans, due to their relatively close evolutionary relatedness and overall similar biology and behavior. Like humans, macaques (1) have primate-specific prefrontal cortical areas that are implicated in neurological disorders,[18] (2) exhibit complex social behaviors that are mediated by similar neural circuits,[19] and (3) undergo extended brain development relative to smaller model species (including, e.g., rodents and marmosets).[20] Furthermore, the wide availability of macaques, combined with our deep knowledge of the species—acquired through over a century of cumulative biological and behavioral study—provide rich context for comparison.

Additional aspects of translatability—including the extent to which model species exhibit human-like brain sex differences—have yet to be tested. This represents a critical knowledge gap, since many species are likely to show non-human-like patterns due to species-specific evolutionary mechanisms (e.g., mate choice, mate competition, and parental care)[21] that determine sex-biased behaviors and neurobiology. This may be particularly relevant to transcriptomes, since (1) sex-biased gene expression patterns tend to be species specific[22] and (2) sex-biased genes evolve faster than non-sex-biased genes (in terms of changes to coding sequences and expression levels).[23–29] Previous transcriptomic studies of rhesus macaque brains did not focus on sex differences and/or had limited sampling of individuals.[30–34] Accordingly, we have little to no understanding of how sex-biased brain gene expression patterns in macaques compare to those in humans or of the evolutionary mechanisms that may have contributed to these differences. These gaps impede our understanding of the utility of the rhesus macaque model and our ability to develop therapies for sex-biased brain disorders in humans.

To address this, we generated one of the largest nonhuman primate brain transcriptional datasets (*n* = 527 samples) and quantified sex differences in gene expression across 15 brain regions (Figure 1A; Table S1) from 36 free-ranging adults (20 females, 16 males, identified using chromosomal and phenotypic sex; Figure S1; Table S2; see STAR Methods). This substantial sample size allowed us to characterize patterns of sex-biased gene expression across the rhesus macaque brain, link these patterns to human sex differences in the brain and disease, and illuminate evolutionary mechanisms underlying these patterns.

RESULTS

Sex-biased gene expression is largely shared across macaque brain regions

We first quantified the drivers of global gene expression variation across all 527 samples for 12,672 expressed genes in 15 regions (Figure 1; Tables S1 and S2; STAR Methods). As expected, the primary driver of variance in macaque brain gene expression was the sampled brain region (mean = 36.12%; Figures 1B and 1C; Table S3), reflecting regional differences in cell composition, developmental origin, and function. Indeed, regions in topographical proximity and with functional overlap exhibited more similar transcriptional profiles (Figures 1B, S2, and S3). Demographic and behavioral factors explained much less variation in the expression of individual genes across the brain (means: sex = 0.50%, dominance rank = 0.41%, age = 0.49%; Figure 1C;

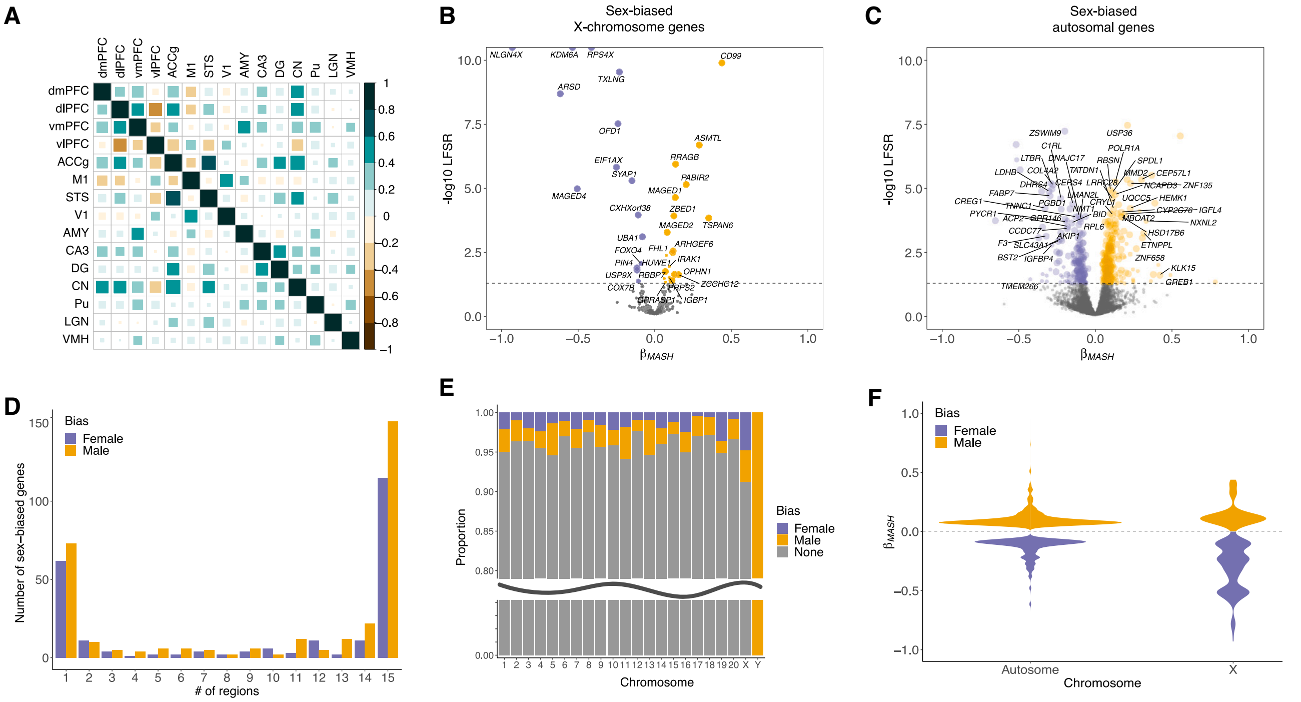

Figure 2. Regional and chromosomal distributions of sex-biased genes in macaque brains

(A) Correlation plot for pre-mashr sex effect sizes (from EMMREML) across regions (Spearman's ρ). Teal = positive correlation, brown = negative correlation, size of square indicates strength of correlation. Of these interregional correlations, 77 are significantly positive, 23 are significantly negative, and are five not significant ($p > 0.05$).

(B) Volcano plot of sex-biased X chromosome genes. Each point = one gene; minimum LFSR (x axis) and maximum β (y axis) across regions are shown; point size is proportional to the # of regions in which the gene is sex biased (LFSR < 0.05); positive β = male biased, negative β = female biased.

(C) As in (B) for sex-biased autosomal genes.

(D) Bar chart of the number of sex-biased genes (LFSR < 0.05) shared across different numbers of regions identified by our primary mashr analyses.

(E) Proportions of genes on each chromosome that are not biased in any region (gray), female biased in at least one region (purple), or male biased in at least one region (yellow). The sex chromosomes are enriched for sex-biased genes (Fisher's exact tests: $p < 0.05$).

(F) Violin plots of sex effect sizes (mashr β) for sex-biased autosomal versus X chromosome genes.

Tables S3 and S4; see Chiou et al.[35] for our analysis of age effects). Sex explained significantly more variance for genes located on sex chromosomes compared to autosomal genes (overall brain means: Y chromosome = 92.74%, X chromosome = 1.16%, autosomes = 0.41%; Tukey's honestly significant difference [HSD] adjusted p [p_{adj}] < 0.001; Figure 1C; Table S3).

Next, we estimated sex biases in gene expression (within each brain region) using linear mixed models controlling for age, dominance rank, technical covariates, and genetic relatedness (see STAR Methods).[36] Sex effects were largely similar across brain regions (73% of regional pairwise correlations were significant and positive) (Figures 2A and S4; Table S5), such that genes more highly expressed in females in one region tended to also be more highly expressed in females in all other regions. This is consistent with observations of shared sex effects across other tissues in multiple species and suggests shared gene regulation across functionally and cellularly distinct tissues.[22] Sex effects were most similar among brain areas involved in macaque sociality (dorsal prefrontal cortex [PFC], anterior cingulate gyrus [ACCg], caudate nucleus [CN], and superior temporal sulcus [STS])[37,38] (Figure 2A; Table S5), which may reflect evolved sex differences in social group size and dominance hierarchy dynamics.[39]

We then implemented joint multivariate analysis across regions to increase power, improve precision of our sex effect estimates, and estimate local false sign rates (LFSRs).[40] LFSRs quantify our confidence in the direction of effect estimates, while less conservative local false discovery rates (LFDRs) measure the confidence that the effect is non-zero.[40] In total, 4.4% (561 out of 12,672) of genes expressed in the brain were differentially expressed between males and females (LFSR < 0.05) in at least one region (Figures 2B, 2C, and 2D; Table S6), similar to human studies (average across eight overlapping brain tissues = 6.5%).[10] Most sex-biased genes were biased same direction in most regions (66.8% were biased in at least eight out of 15 tissues; Figure 2D). Of the identified sex-biased genes, 7.1% were located on the X chromosome, 1.6% on the Y chromosome, and 91.3% on autosomes (Figure 2E; chromosomal enrichment analyses are below). Female-biased X chromosome genes exhibited larger sex effects ($n = 22$ genes; mean $|\beta| = 0.32$) than either male-biased X chromosome genes ($n = 18$; mean $|\beta| = 0.15$; Tukey's HSD $p_{adj} < 0.001$) or sex-biased autosomal genes (female, $n = 218$, mean $|\beta| = 0.14$, $p_{adj} < 0.001$; male, $n = 294$, mean $|\beta| = 0.11$, $p_{adj} < 0.001$) (Figure 2F), which is likely to reflect escape from X chromosome inactivation (XCI). All female-biased X-linked genes that we identified

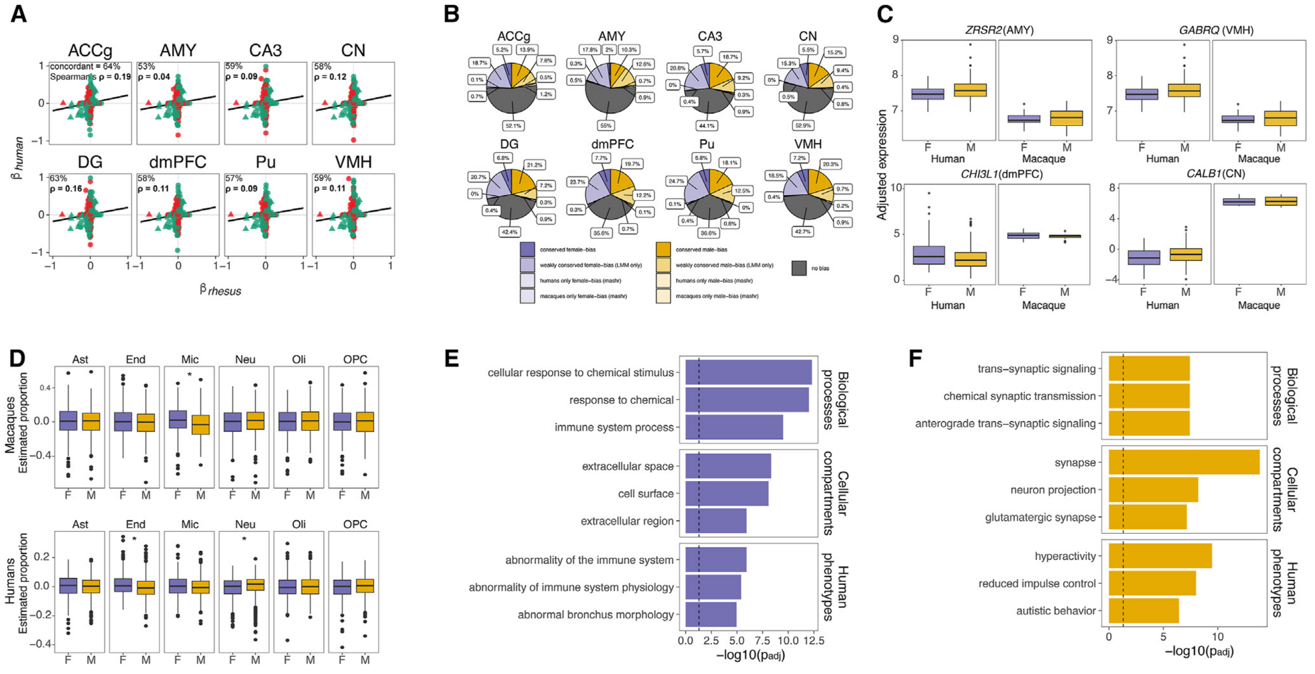

Figure 3. Comparisons of sex-biased gene expression patterns, cell types, and biological processes in macaque and human brains

(A) Scatterplots of estimated sex effects (mashr β) for all one-to-one orthologous genes (excluding the Y chromosome) in humans (GTEx) versus rhesus macaques (this study) (circles = autosomal genes; triangles = X chromosome genes). Green points represent genes with concordant sex bias (mashr β) across species, while red points represent discordance. Significant correlations (Spearman's ρ; $p < 0.05$) are in bold.

(B) Pie charts showing the proportions of genes with (1) conserved sex bias ($p_{adj} < 0.05$ for sex in LMMs and mashr βs estimated in the concordant direction in both species; see STAR Methods); (2) "weakly" conserved sex bias ($p_{adj} < 0.05$ for sex in LMMs but inconsistent mashr βs); (3) sex bias in one species only ($p_{adj} > 0.05$ for sex in LMMs and mashr LFSR < 0.05 in one species only); or (4) no sex bias. No genes were identified as having statistically significant divergent sex bias (both mashr LFSRs < 0.05 but mashr βs in opposite directions). Note that we did not detect any genes with human-specific female-bias in the dorsomedial prefrontal cortex (dmPFC) or ventromedial hypothalamus (VMH) (no percentages are shown for these sets).

(C) Examples of conserved sex-biased genes in humans and macaques. Boxplots show covariate-adjusted expression levels for each sex within each species. Genes depicted include *ZRSR2* in the amygdala (female biased, undergoes XCI escape), *GABRQ* in the hypothalamus (male biased, associated with ASD), *CHI3L1* in the prefrontal cortex (female biased, associated with schizophrenia), and *CALB1* (male biased, associated with epilepsy).

(D) Boxplots show estimated relative cell-type proportions (i.e., SPVs from BRETIGEA, see STAR Methods) within each sex for macaques (top) and humans (bottom) for each of six brain cell types. Significant sex differences are indicated with an asterisk (*) (t test: *$p < 0.05$).

(E) g:Profiler enrichment results for genes with conserved female-biased expression in humans and macaques. Top three terms (with lowest $p_{adj} < 0.05$ [adjusted using default g:SCS], shown on the x axis) are shown for biological processes (GO:BP), cellular compartments (GO:CC), and human phenotypes (HP).

(F) As in (E) for conserved male-biased genes.

in macaques are known XCI escapees in humans,[10] which may suggest some level of conserved XCI escape across species. Many human XCI escapees were not sex biased in our dataset, which may reflect cross-species and within-species cross-tissue variation in XCI escape.[41] Notably, some other model organisms, including mice, exhibit a relatively low rate of XCI escape,[42] which may further limit their translatability for sex-linked human conditions.

Sex-biased brain gene expression is similar in rhesus macaques and humans

To investigate whether humans and rhesus macaques exhibit similar sex differences in brain gene expression, we first compared estimated sex effects from this study (described above) to those from an analysis of the human GTEx data (V8) for eight overlapping brain regions (controlling for age and technical effects; see STAR Methods) (Table S6). Similar to our findings in macaques, sex explained 0.49% of the variation in gene expression on average across the human GTEx brain samples. Three genes were located on the X chromosome of one species and on an autosome of the other, and, for these genes, location on the X chromosome was associated with greater female-biased expression in that species (X chromosome in macaques = *HNRNPA1*, in humans = *HMGB3*, *SLC25A6*) (Table S6). We found that transcriptome-wide sex effects were similar across species in each region (Figure 3A), since estimated sex differences in gene expression (mashr βs) were positively and significantly correlated with each other across all one-to-one orthologs, with stronger concordance for sex chromosome genes (autosomal genes, n = 8,240, ρ = 0.03–0.18 [mean = 0.10]; X chromosome genes, n = 286, ρ = 0.22–0.38 [0.32]; non-Y chromosome genes, n = 8,529, ρ = 0.04–0.19 [0.11]; all $p_{adj} < 0.05$) (Figures 3Aand S5; Tables S6 and S7). The magnitude of transcriptome-wide sharing of sex effects between human and macaque brains approached the values reported in previous studies of other tissues and species (e.g., for orthologous

CellPress
OPEN ACCESS

autosomal genes in the spleens of pheasants and peafowls, which diverged ~30 million years ago [mya], $\rho \sim 0.2$).[25] Correlations are higher for cross-species comparisons of gonadal tissue (e.g., pheasants vs. peafowls, $\rho = 0.7$)[25] and whole bodies (e.g., among seven *Drosophila* species that diverged ~50 mya, $\rho = 0.5$),[43] and also for cross-tissue comparisons within the same species (humans, average ρ between brain tissues and all tissues = 0.5).[10] We did not detect a relationship between sex bias and gene homology (e.g., in macaques, sex-biased vs. not, one-to-one ortholog vs. not; Fisher's exact test, odds ratio [OR] = 1.14; $p = 0.31$).

We also present comparisons of sex effects on human and macaque brains using various LFSR cutoffs (Table S7). When these comparisons were restricted to genes that passed a certain threshold in both species, this produced very limited gene sets (Tables S6 and S7). For example, only four autosomal genes were detected in both species at LFSR < 0.05 (male biased, *GAD1*; female biased, *EMP3*, *IGFBP4*, and *SLC16A7*) (Table S6). This is likely to reflect, in part, the small amount of variance explained by sex (Figure 1C; Table S4), combined with differences in power between the datasets. Accordingly, we employed a complementary approach to identify genes with conserved sex bias across both species using linear mixed models (LMMs).[22] We modeled covariate-adjusted expression levels as a function of sex (fixed effect) and species (random effect) using an approach that accounts for between-species differences in sample size and variability (STAR Methods). We identified conserved genes (within each brain region) as those with significant sex terms in the LMMs ($p_{adj} < 0.05$) and mashr βs estimated in the consistent direction in both species-specific analyses. In total, 51.9% (5,043 out of 9,724) of expressed one-to-one orthologous genes exhibited a conserved sex bias in at least one region, most of which were male biased (male biased, 77.8%, $n = 3{,}922$ unique genes; female biased, 21.9%, $n = 1{,}105$; inconsistent bias across regions, 0.3%, $n = 16$) (Figures 3B and 3C). This analysis detected more sex-biased genes than the species-specific analyses in part due to gains in power from the larger, species-combined sample size (STAR Methods). We also identified about twice as many conserved genes compared to Naqvi et al.[22] (proportion of genes conserved = 24%), which is expected given that they examined more distantly related species (i.e., humans, macaques, dogs, and rodents) and more differentiated tissues (e.g., brain, skin, lung). Many species-specific sex-biased genes (i.e., those with mashr LFSRs < 0.05) were also identified as conserved in the LMM analysis (macaques, 485 out of 561 sex-biased genes were one-to-one orthologs, 163 out of 485 [34%] orthologs were conserved; humans, 164 out of 183, 118 out of 164 [72%]). Together, these observations suggest that global transcriptomic sex differences in macaque and human brains are similar in many ways across both cortical and subcortical brain areas.

Sex-biased brain gene expression in humans and macaques partially reflects sex differences in microglia

Observed sex differences in gene expression could reflect differences in cell composition and/or the expression of specific genes within cell types. To test the contribution of sex differences in cell-type proportions/states to sex-biased gene expression in macaque and human brains, we drew on a recent meta-analysis of human brain cell-type markers.[44] For macaques, we found that female-biased genes (LFSR < 0.05 in any region; $n = 270$) were enriched for microglial markers (OR = 2.51; $p_{adj} = 0.010$; Table S8). We also applied cell-type deconvolution, which estimates the abundance of different cell types in each bulk sample based on the expression of marker genes (STAR Methods). Females exhibited higher estimated proportions (i.e., surrogate proportion variables [SPVs]) of microglia across regions (microglia, $p_{adj} = 0.001$; all other cell types, $p_{adj} > 0.05$) (Figures 3D and S6; Table S9). Similarly, our analysis of the human GTEx data showed that female-biased genes were enriched for microglial markers (OR = 4.607, $p_{adj} = 0.003$) and endothelial markers (OR = 17.233, $p_{adj} < 0.001$) (Table S8), although sex differences in SPVs were only significant for endothelial cells ($p_{adj} < 0.001$) (Figure 3D; Table S9). These findings are consistent with (1) previous analyses of the human GTEx data,[45] (2) the presence of a neurodevelopmental gene co-expression module in postnatal human brains that is enriched for both microglial markers and female-biased genes (ME3),[46] (3) reports of female-biased microglial proportions in adult humans,[47] and (4) our finding that human-macaque conserved female-biased genes (LMM $p_{adj} < 0.05$ and mashr $\beta < 0$; $n = 1{,}105$ genes; STAR Methods) were enriched for immune-related biological processes (*g:Profiler* $p_{adj} < 0.05$) (Figure 3E; Tables S10–S12). This shared pattern may reflect evolved sex differences in immune surveillance and response due to the need for birthing people to tolerate an internal, immunologically challenging pregnancy.[48] Furthermore, differences in microglial number, activation, and maturation during development drive sexual differentiation in the brain and are observed across brain disorders and diseases.[49–53] In humans, we also found that male-biased genes were enriched for neuronal markers (OR = 23.07, $p_{adj} < 0.001$) (Table S8) and SPVs for neurons were higher in males ($p_{adj} = 0.004$) (Figure 3D, and Table S9). These results are consistent with (1) reports of male-biased neuronal proportions in humans[54,55] and (2) our finding that human-macaque conserved male-biased genes (LMM $p_{adj} < 0.05$ and mashr $\beta > 0$; $n = 3{,}922$ genes; STAR Methods) were enriched for synaptic functions (*g:Profiler* $p_{adj} < 0.05$) (Figure 3F; Tables S10–S12). This shared pattern may reflect sex differences in average brain size and synaptic density.[56,57] Notably, genes without a conserved sex bias ($n = 4{,}681$) were enriched for very few biological processes (Table S12).

To identify sex-biased gene expression patterns that are not driven by the aforementioned sex differences in cell-type abundances, we estimated sex effects after performing cell-type deconvolution analysis on the macaque expression data (STAR Methods; Table S13) and repeated all enrichment analyses described below using these data. Estimated sex effects tended to be in the same direction (i.e., male or female biased) whether cell-type proportions were considered, and, as expected, fewer sex-biased genes passed our LFSR threshold in this analysis (25% fewer; $n = 422$ genes exhibited sex bias in at least one region) (Figures 4A–4C, S7, and S8).

Many sex-biased genes are regulated by sex hormones

To illuminate the regulatory mechanisms underlying sex-biased gene expression in macaque brains, and to compare these

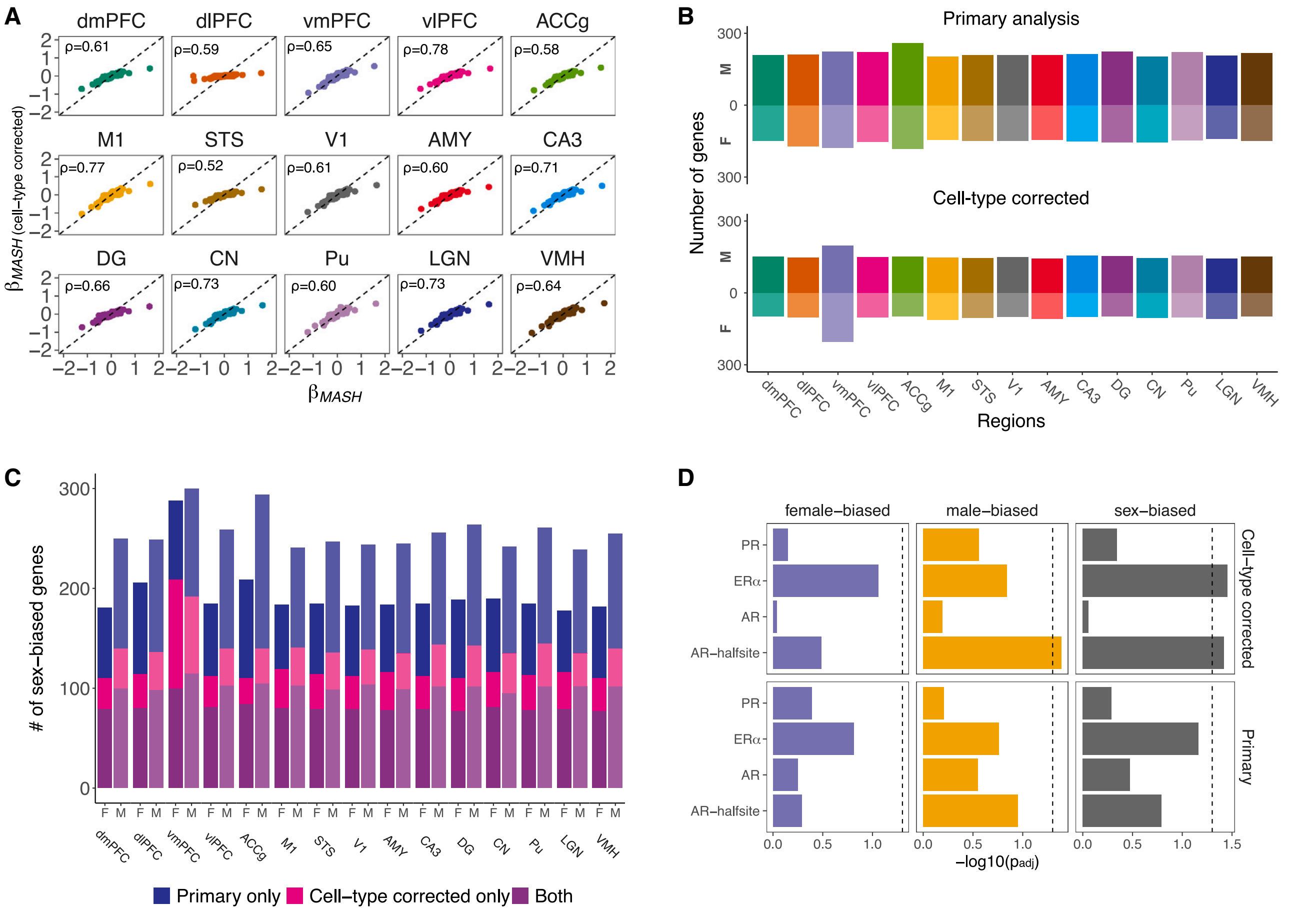

Figure 4. Cell-type-corrected sex-biased gene expression in macaque brains

(A) Scatterplots of sex effects (mashr β) from our unadjusted (primary) analyses (x axis) versus cell-type-corrected analyses (y axis) for each region (dashed line: intercept = 0, slope = 1). Spearman's ρ values are shown.

(B) Counts of sex-biased genes identified by mashr (LFSR < 0.05) using unadjusted (top) and cell-type-corrected (bottom) expression data. M = male-biased, F = female-biased.

(C) Stacked bar plots of the number of male- and female-biased genes identified per region in our primary and/or cell-type corrected analyses. M, male biased; F, female biased.

(D) Bar plots of enrichment results for sex-hormone-related motifs among male-, female-, and sex-biased genes (LFSR < 0.05 in at least one region) identified in our primary and cell-type corrected analyses. Motifs are listed on the y axis (PR, progesterone; ERα, estrogen alpha; AR, androgen) and −log10(p values) from hypergeometric enrichments are on the x axis.

mechanisms with those in humans, we identified motifs that were present within the promoters of sex-biased genes more often than those of non-biased genes (STAR Methods; Figures 4D and S9D; Tables S14 and S15). The promoters of sex-biased genes in macaque brains were enriched for estrogen and androgen binding site motifs after accounting for cell-type effects (ERα, OR = 1.274, p = 0.035; AR half-site, OR = 1.031, p = 0.038) (Figure 4D; Table S15). Separate analyses of male- and female-biased genes demonstrated associations with androgen and estrogen binding, respectively (Figure 4D; Table S15). Although enrichments for sex hormone binding sites only approached significance in the uncorrected analyses (ERα: OR = 1.192, p = 0.069; Figure 4D), the most highly enriched motifs in both analyses were for transcription factors (TFs) that interact with estrogens (e.g., SP1, which binds to estrogen receptor)[58] (Table S14). Interestingly, the SF1 motif was enriched among sex-biased genes and the *SF1* gene exhibited sex differences in expression in our dataset (cell-type-corrected, motif enrichment p = 0.010, male-biased LFSR < 0.05 in all regions; uncorrected, motif enrichment p = 0.084, male-biased LFSR < 0.2 in all regions). These results are consistent with studies of humans, which suggest that sex-biased autosomal genes are directly and indirectly modulated by sex hormones.[12] Although previous work linked sex-biased expression in human brains solely with androgen regulation,[14] this study focused on sex-biased splicing patterns and included many post-menopausal women.[14] Many other enriched motifs identified here also regulate sex-biased gene expression across human tissues (e.g., estrogens, NRF1, and ELK1)[10] and/or have sex-biased regulatory targeting patterns in humans (e.g., MAZ, IRF8, and Nkx2.1).[11] These similarities may reflect some conserved regulatory mechanisms across species and

CellPress
OPEN ACCESS

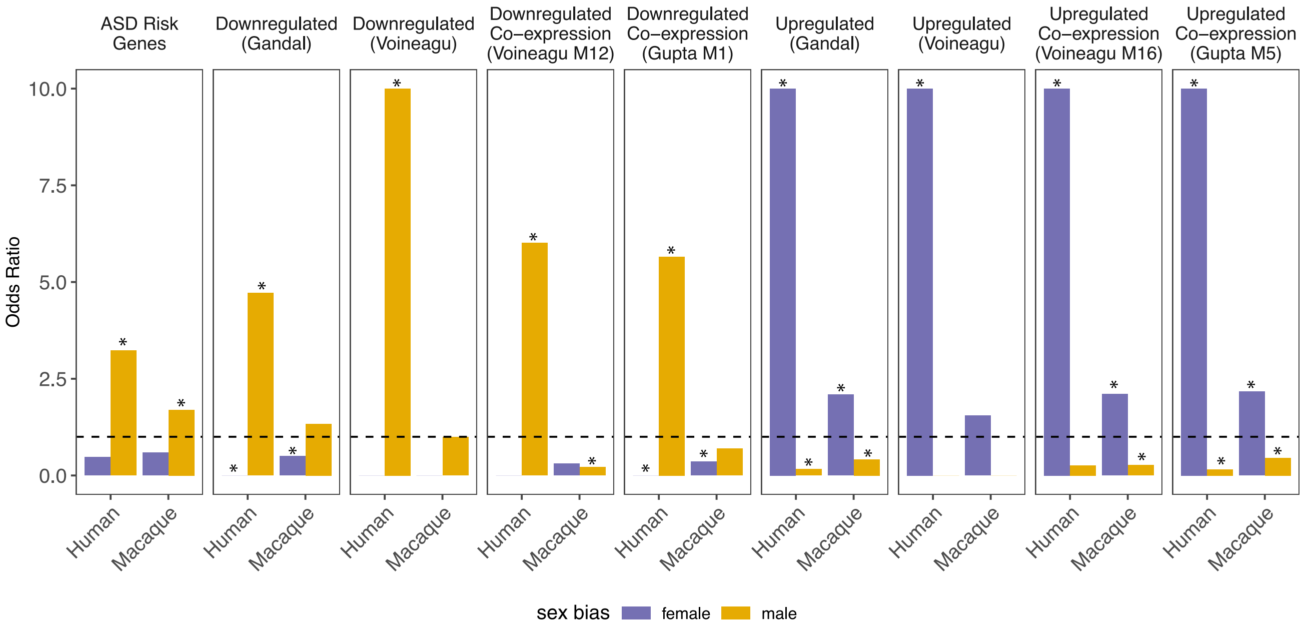

Figure 5. Sex-biased genes in macaque and human brains are enriched for similar ASD-related gene sets

Enrichment results (odds ratios [ORs] from Fisher's exact tests) linking ASD-related gene sets to sex-biased genes in human and macaque brains. ASD risk genes are from the DISEASES database (DOID: 12849); ASD down- and upregulated gene sets are from Gandal and colleagues,[59] Voineagu and colleagues,[60] or Gupta and colleagues.[61] Dashed line represents OR = 1. *$p < 0.05$. For visualization purposes, we limited the y axis to a maximum OR of 10.

tissues, although we also identified potentially divergent mechanisms (i.e., motifs that were enriched among sex-biased genes in the current dataset but have not been reported in studies of humans[10,11]; e.g., ZNF264).

Sex-biased genes are implicated in sex-biased neurological disorders

Given that sex-biased genes in human brains have been linked to sex-biased neurological conditions,[9–17] we investigated whether sex-biased genes in macaque brains were similarly enriched for disease risk genes. We used three complementary approaches that differ in their consideration of the relative order and/or magnitude of per-gene sex effects, namely (1) Fisher's exact tests, (2) Kolmogorov-Smirnov (K-S) tests, and (3) gene set enrichment analysis (GSEA) (see STAR Methods). Fisher's exact tests and K-S tests showed that male-biased gene expression in macaque brains was linked to risk genes for multiple neurodevelopmental and psychiatric conditions, including ASD, intellectual disability, schizophrenia, epilepsy, bipolar disorder, and ADHD (all $p_{adj} < 0.05$) (Figure 5; Table S16). All three methods produced similar enrichment scores, although associations were not significant according to GSEA (Table S16; Figure S10). Consistent with this, we also found that (1) male-biased gene expression in the human GTEx data was associated with risk genes for neurodevelopmental and psychiatric conditions across all three enrichment approaches (all $p_{adj} < 0.05$) (Figures 5 and S10; Table S17), and (2) genes with conserved male bias across humans and macaques (LMM $p_{adj} < 0.05$ and mashr $\beta > 0$; $n = 3{,}922$ genes) were enriched for ASD and ADHD (*g:Profiler* $p_{adj} < 0.05$) (Table S11). Drivers of these shared enrichments include male-biased expression of *GAD1* in both species (LFSRs < 0.05), a gene that controls γ-aminobutyric acid (GABA) synthesis and is implicated in multiple neurodevelopmental conditions (e.g., ASD). These findings are similar to previous studies of human brains[12–14] (but cf. Werling et al.[9]) and suggest that sex differences present in typically developing individuals (i.e., normative sex differences in gene expression) modulate the relative impact of genetic risk variants, thereby contributing to sex differences in disease prevalence.[9] Female-biased gene expression was not associated with any neurodevelopmental or psychiatric conditions in macaque or human brains (Tables S16 and S17).

To further investigate links between human disorders and sex-biased genes in macaque brains, we tested whether these genes were enriched for genes that exhibit altered expression levels in the brains of people with ASD. Female-biased genes were associated with cortex-wide ASD-upregulated genes identified by Gandal et al.[59] (OR = 2.091, $p < 0.001$) and with genes in ASD-upregulated microglial co-expression modules (Voineagu et al.[60] [M16], OR = 2.108, $p = 0.030$; Gupta et al.[61] [M5], OR = 2.172, $p = 0.002$) (Figure 5; Table S18). Male-biased genes were depleted for ASD-upregulated genes (OR = 0.414, $p = 0.001$) and co-expression modules (M16,[60] OR = 0.265, $p = 0.049$; M5,[61] OR = 0.453, $p = 0.040$) and enriched for ASD-downregulated genes (OR = 1.301, $p = 0.086$) (Figure 5; Table S18).[59] Similar patterns were recovered in our analysis of the human GTEx data, which, as expected, showed even stronger enrichments among female-biased genes (ASD-upregulated genes from Gandal et al.,[59] OR = 14.906, $p < 0.001$; from Voineagu et al.,[60] OR = 29.918, $p < 0.001$; M16,[60] OR = 41.638, $p < 0.001$; M5,[61] OR = 28.193, $p < 0.001$) and among

male-biased genes (ASD-downregulated genes from Gandal et al.,[59] OR = 4.719, $p < 0.001$; from Voineagu et al.,[60] OR = 11.582, $p < 0.001$; M12,[60] OR = 6.013, $p < 0.001$; M1,[61] OR = 5.654, $p < 0.001$) (Figure 5; Table S18).

After cell-type correction, we did not find any significant enrichment of disease risk genes or of ASD differentially expressed genes (Table S19), even though enrichment scores were correlated across analyses ($\rho = \sim 0.7$) (Figure S9). Combined with our primary analyses (i.e., not accounting for cell composition), these findings suggest that sex differences in cell-type proportions and states may contribute, in part, to sex differences in the expression of disease-related risk genes (in typically developing individuals) and thus sex differences in disease susceptibility. In particular, our finding that ASD risk genes tend to be more highly expressed in male brains may reflect that (1) ASD risk genes tend to be expressed in neurons[59,60,62–64] and (2) male brains may contain a higher proportion of neurons.[54,55] This highlights a potential mechanism through which male brains may be affected more strongly by genetic alterations associated with ASD. These genetic alterations are thought to affect brain development in ways that produce dysregulated neuronal activity and microglial responses (e.g., higher densities of larger, more activated microglia),[53] and these changes are reflected by global downregulation of neuronal, synaptic genes and upregulation of microglial, immune-related genes in ASD.[59–61,63,65] Accordingly, our findings linking ASD-downregulated neuronal and ASD-upregulated microglial genes to male-biased and female-biased genes, respectively, may reflect transcriptomic convergence between typical sex differences and ASD-related alterations[59–61,63,65] in neuronal and microglial abundance and function. In fact, many female-biased, ASD-upregulated genes in both species were also more highly expressed in microglia, and, among genes identified as female biased in either species ($n = 249$), genes that were also upregulated in ASD ($n = 66$) were enriched for cytokine- and immune-related pathways ($p < 0.05$) (Table S20).

Accurate sex prediction in macaques requires few genes and is less reliable in older individuals

To investigate heterogeneity in sex-biased gene expression across individuals (of the same sex) and to identify potential drivers of this variation, we constructed and evaluated region-specific sex prediction models of the rhesus macaque brain transcriptome (model construction repeated using three gene sets [non-Y genes, X chromosome genes only, autosomal genes only] within each region, resulting in 15 regions × 3 gene sets = 45 models total). We could accurately predict sex from the expression levels of relatively few genes (models of non-Y genes: mean accuracy = 0.977, mean number of genes = 39; models of X chromosome genes and autosomal genes performed similarly; Table S21), similar to previous work in human tissues.[10]

Non-Y gene models tended to be better at correctly classifying females than males (Figures 6A and S11; Tables S21–S23), which may reflect that, of genes that were influential in at least one region ($n = 501$), X chromosome genes were more influential than autosomal genes (average of summed relative influence: X chromosome, mean = 14.28; autosomes, mean = 8.42) (Tables S21–S23). Accuracy was also lower in predicting the sex of older individuals (linear regression of known sex probability modeled as a function of age: $p = 0.016$) in models of autosomal genes (Figure S12). This effect was stronger among males of all ages and older individuals (>8 years) (Figures 6B, S12, and S13). In fact, the most often misclassified individual was also the oldest male in our sample (misclassified as female in seven out of 45 models of this individual, spanning three different regions and all gene sets; out of 527 samples × 3 gene sets = 1,581 classifications; there were only 40 [2.5%] misclassifications total) (Table S23). These sex and age differences in prediction accuracy may reflect that males, particularly older males (>8 years), exhibit higher within-sex gene expression variation compared to females (median pairwise Euclidean distance of residual gene expression among: old males = 137.9, young males = 135.9, old females = 134.2, young females = 133.7; all differences were significant except young males vs. old females, Tukey's HSD $p_{adj} < 0.05$; Figure 6C). This is in line with age-related declines in the control of molecular phenotypes, which increase variance of molecular and anatomical phenotypes in older individuals.[66,67] Prediction accuracy may also be affected by interactive effects of sex and age on brain transcriptomes. In fact, the most influential genes for the autosomal models include multiple genes that we previously found to be affected by age,[35] and some of these age-related changes appear to occur in one sex only (Table S24; see STAR Methods), suggestive of sex differences in brain aging (beyond the scope of the current manuscript).

Models of X chromosome genes highlighted similarities with humans, in that the most influential genes in this study were also the most influential genes in models constructed in a recent study across 44 human tissues[10] ($n = 150$ one-to-one orthologs with non-zero influence in both studies; $\rho = 0.222$, $p = 0.006$; Figures 6D and S14). This reflects species similarities in the magnitude of sex-biased expression across X chromosome genes ($\rho > 0.69$ across eight overlapping regions with the human GTEx data; Figures 3A and S5; Table S7).

Rapid evolution of sex-biased genes is likely to reflect higher tissue specificity and genetic variance

To better understand the evolutionary dynamics underlying sex differences in macaque brain transcriptomes, we examined four mechanisms that may facilitate the rapid evolution of sex-biased gene expression observed in other studies: (1) their tendency to be located on sex chromosomes (due to sex-specific patterns of selection and inheritance[68]), since the smaller effective population size of these chromosomes may lead their genes to evolve more rapidly[69]; (2) higher tissue specificity (i.e., lower pleiotropy),[70,71] since pleiotropy may constrain evolutionary change due to widespread multivariate stabilizing selection[72,73]; (3) higher genetic variance in gene expression, since genes whose expression is attributable to genetic variance (vs. environmental variance) can better respond to selection[71]; and (4) higher genic tolerance, since this would allow for more coding sequence mutations without losing function.

We found that sex-biased genes tend to be located on the sex chromosomes (mechanism 1 above) and that sex

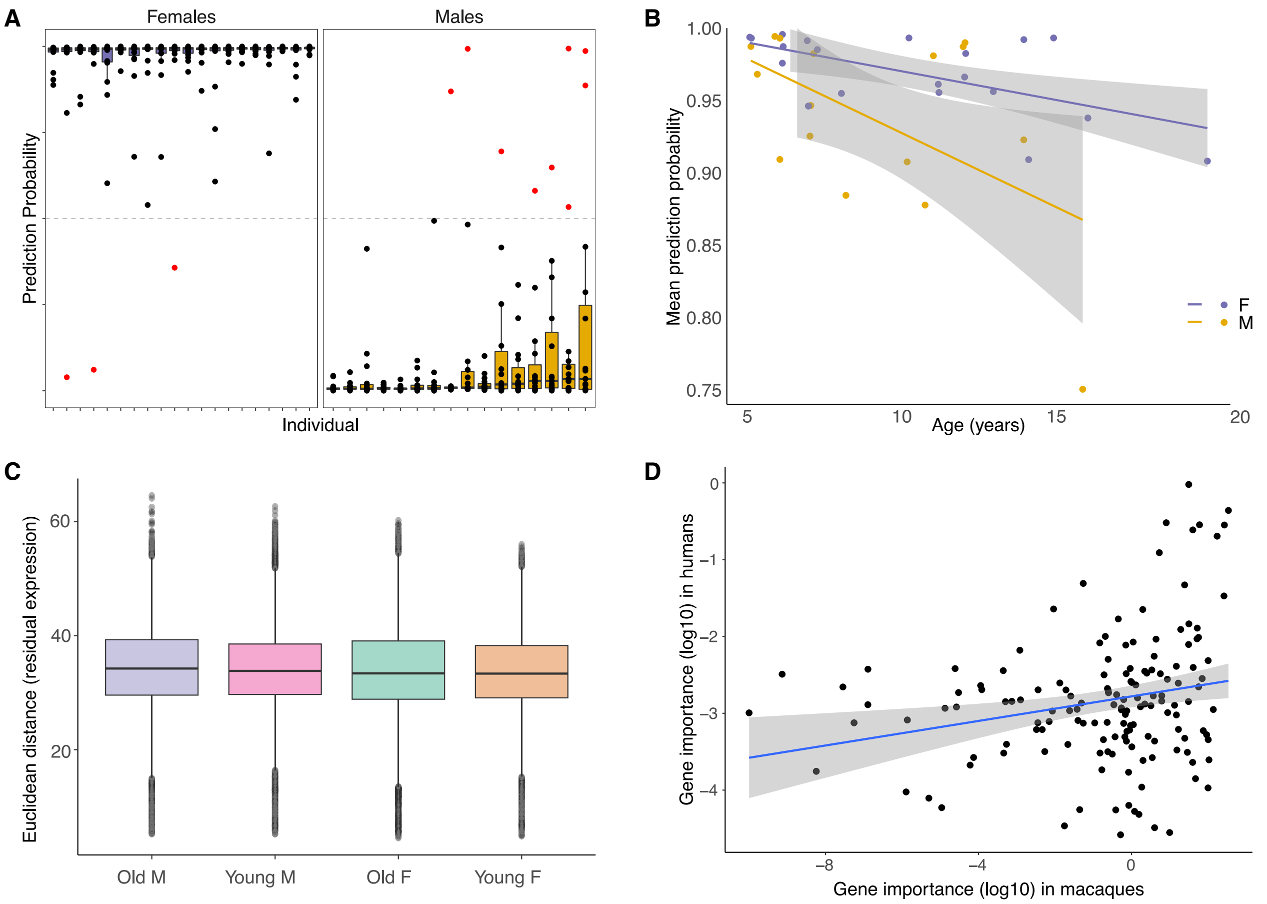

Figure 6. Sex-prediction modeling highlights age effects and similarities between macaque and human brains

(A) Boxplots of prediction probabilities of the known sex per individual (from models of non-Y chromosome genes). Top = higher probability of being female. Bottom = higher probability of being male. Dots indicate values for individual samples. Purple boxes = female, yellow boxes = male, black dots = correctly classified samples, red dots = incorrectly classified samples (prediction probability of correct sex < 0.5). Boxplots indicate the median (black horizontal line), first and third quartiles IQR (lower and upper hinges), and ranges extending from each to 1.5 × IQR beyond each hinge (whiskers).

(B) Prediction probability (averaged across regions) of known sex per individual as a function of age (years) for females (purple) and males (yellow) from models of autosomal genes only.

(C) Boxplots of pairwise Euclidean distances of residual expression (for genes that are informative in any autosomal sex prediction modal, n = 718). Old M/F, males/females > 8 years old; Young M/F, males/females < 8 years old. All differences were significant except young males vs. old females, Tukey's HSD $padj$ < 0.05.

(D) Relative importance of X chromosome genes for sex prediction in X chromosome gene models (summed across regions) in the current study and Oliva and colleagues[10] (ρ = 0.222, p = 0.006).

differences in expression were positively correlated with tissue specificity (2) and genetic variance (3), but not genic tolerance (4). Specifically, (1) the X and Y chromosomes were enriched for sex-biased genes (X chromosome, OR = 2.16; p_{adj} = 0.002; Y chromosome, OR = Inf; p_{adj} < 0.001), and these enrichments were driven by female- and male-biased genes, respectively (female-biased X chromosome, OR = 2.79; p_{adj} = 0.004; male-biased X chromosome, OR = 1.62; p_{adj} = 1; Y chromosome expression is male specific) (Figure 2E). Female-biased gene enrichment on the X chromosome is consistent with a preponderance of female-beneficial mutations that are dominant, since these mutations occur in females two-thirds of the time and are, therefore, selected for (in females) more often than selected against (in males).[68] (2) Tissue specificity estimates ranged from 0.018 to 1 (mean = 0.172; SD = 0.148; Table S25) and genes exhibiting larger sex differences in residual expression also showed more tissue-specific expression (ρ = 0.332; p < 2.2e−16) (Figure 7A). (3) The structure of our data resulted in a bimodal distribution for estimates of genetic variance (Vu) (see STAR Methods; Figure S15), so we evaluated the relationship between log(Vu) and sex differences in residual expression separately within each distribution and found significant positive associations in both (upper distribution, ρ = 0.234, p < 2.2e−16; lower distribution, ρ = 0.290, p < 2.2e−16) (Figures 7B and S16). (4) We did not detect a relationship between absolute sex differences in residual expression and loss-of-function (LOF) mutation tolerance (ρ = 0.006, p = 0.622) (Figure 7C).

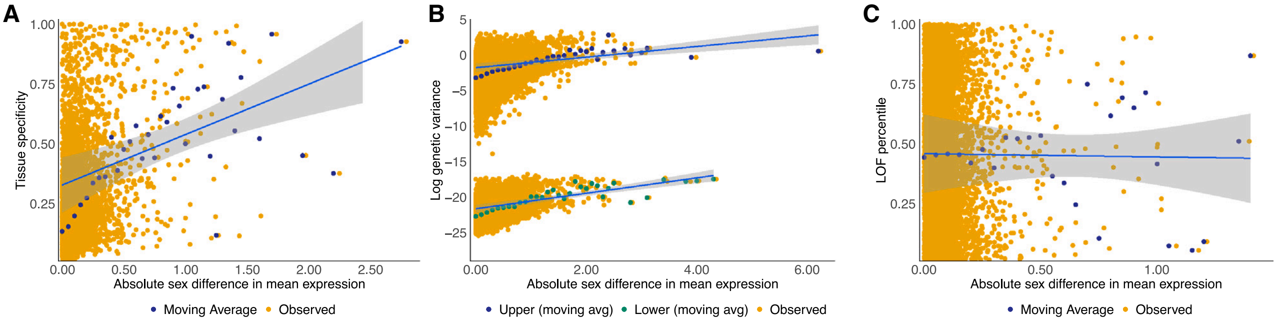

Figure 7. Evolutionary characteristics of sex-biased gene expression in macaque brains

(A) Tissue specificity as a function of the absolute difference in mean residual expression per gene (averaged across regions) (n = 12,663 non-Y chromosome genes) ($\rho = 0.332$; $p < 2.2e{-16}$).

(B) Genetic variance (log) as a function of the absolute difference in mean residual expression per gene and region (n = 152,431 non-Y chromosome gene × region combinations) (upper distribution, $\rho = 0.234$, $p < 2.2e{-16}$; lower distribution, $\rho = 0.290$, $p < 2.2e{-16}$).

(C) Loss-of-function (LOF) tolerance as a function of the absolute difference in mean residual expression per gene (averaged across regions) (n = 7,786 non-Y chromosome one-to-one orthologs in the LOFtools database) ($\rho = 0.006$, $p = 0.622$).

DISCUSSION

This work provides an in-depth characterization of the patterns, biological functions, disease associations, regulatory factors, and evolutionary mechanisms relevant to sex-biased gene expression in rhesus macaque brains. Our work suggests that sex differences in rhesus macaque brain transcriptomes are similar to those reported in humans, bolstering the translatability of this indispensable model species for studies of sex-biased neurological conditions. Not only are gene expression levels biased in the same direction (i.e., female or male biased) across species in multiple brain areas, but adult macaque and human brains appear to share sex-hormone-mediated regulation of sex-biased genes, upregulation of the neuroimmune system in females, and sex-biased expression of genes implicated in sex-biased brain disorders, including ASD. Furthermore, we highlight mechanisms that contribute to the rapid evolution of sex-biased genes, including their tendencies to be located on sex chromosomes and to exhibit greater genetic variance for expression and tissue-specific patterns of expression.

Limitations of the study

Given that our results suggest that sex-biased gene expression partly reflects sex differences in the proportion of different cell types, we suggest that future studies analyze both uncorrected and cell-type-corrected data, ensuring that the latter are produced by removing compositional variation prior to modeling sex effects. Studies using single-nucleus RNA sequencing will allow us to more directly examine sex differences in brain cell-type proportions and states, in addition to cell-type-specific gene expression patterns. Analyses of bulk tissue remain important since they provide a fuller picture of cellular activity by capturing cytoplasmic RNA.[74]

We also find that female-biased genes in human and macaque brains overlap with upregulated genes in ASD. Although these results appear to contrast with previous work linking *male-biased* genes in human brains to microglial markers and ASD-upregulated genes,[9,75] differences in sample size and developmental period may explain the apparent discrepancy. Our analyses include more samples for both the ASD and normative sex datasets, but these data represent different developmental periods (i.e., the ASD dataset analyzed here includes children and adults[59]; the macaque and human normative datasets include adults only). This may affect results since ASD and sex both represent "developmentally moving targets."[75] Recent studies (with larger sample sizes) report that ASD-upregulated microglial/immune gene modules are male biased prenatally but are then female biased during certain postnatal periods.[75] This is consistent with prenatally male-biased and postnatally female-biased expression of a microglia-enriched neurodevelopmental gene module in the human brain (ME3),[46] in addition to reversal of sex differences in microglial colonization and activation prior to adolescence in rats.[76] Future comparative studies of early neurodevelopmental periods will be required to confirm the extent to which sex-biased expression of human disease-linked genes are conserved across species.

Throughout, we used the term biological "sex" to refer to a label that nonhuman animals are assigned by researchers and that people are assigned at birth based on their anatomy, which typically corresponds with one of two sex chromosome complements. We use the phrase "sex differences" to refer to group-level average differences between individuals with "typical" (of the majority) anatomical and sex chromosome complements (i.e., XY males with testes, XX females with ovaries), although we acknowledge that these criteria are not confirmed in many of the human studies discussed here (which instead rely on self-identification). In addition, while most people are categorized as female or male at birth, sex is not strictly binary. In fact, about 1% of the population exhibits variable (i.e., nontypical of the majority) sex chromosome combinations, sex hormone concentrations and receptors, and bodily phenotypes. We also distinguish sex from gender, a culturally defined and malleable concept. A person's gender need not align with their assigned sex, and since an individual's experiences in society can be affected by their perceived gender, these biological and

psychosocial influences are difficult (if not impossible) to disentangle in humans. Possible implications of the molecular sex differences reported here should not be extrapolated beyond what is demonstrated in the current study.[77,78]

STAR★METHODS

Detailed methods are provided in the online version of this paper and include the following:

- KEY RESOURCES TABLE
- RESOURCE AVAILABILITY
  - Lead contact
  - Materials availability
  - Data and code availability
- METHOD DETAILS
  - Tissue procurement and processing
  - Tissue dissection
  - RNA extraction
  - RNA quality assessment
  - Library preparation and sequencing
  - Reference genomes and read alignment
  - Quality assessment
  - Read normalization
  - Genotyping
  - Behavioral data collection
- QUANTIFICATION AND STATISTICAL ANALYSIS
  - Dimensionality reduction
  - Hierarchical clustering
  - Variance partitioning
  - Modeling sex effects on gene expression
  - Multivariate adaptive shrinkage (MASH)
  - Human (GTEx) comparison
  - Conservation analysis
  - Cell type enrichment analysis
  - Deconvoluting cell type proportions and modeling sex effects on gene expression
  - Gene ontology enrichment analyses
  - Disease gene set enrichment analysis
  - Functional enrichment analyses of conserved/divergent genes
  - Motif enrichment analyses
  - Sex prediction
  - Sex differences in gene expression heterogeneity
  - Interactive sex and aging effects
  - Chromosome overrepresentation analysis
  - Tissue specificity
  - Comparisons of loss-of-function (LOF) mutation tolerance
  - Comparisons of genetic variance

SUPPLEMENTAL INFORMATION

Supplemental information can be found online at https://doi.org/10.1016/j.xgen.2024.100589.

ACKNOWLEDGMENTS

We thank those who make our research possible, particularly the Caribbean Primate Research Center and staff from both the Cayo Santiago Field Station and the Sabana Seca Field Station. We thank Madeleine Rosenstein for assisting in data collection and Dr. Armin Raznahan for providing valuable feedback. We also thank the reviewers for their suggestions and help with the paper.

The Genotype-Tissue Expression (GTEx) Project was supported by the Common Fund of the Office of the Director of the National Institutes of Health (commonfund.nih.gov/GTEx) and by NCI, NHGRI, NHLBI, NIDA, NIMH, and NINDS. The datasets used for the analyses described in this manuscript were obtained from dbGaP at http://www.ncbi.nlm.nih.gov/gap through dbGaP accession number phs000424.v8.p2.

Funding support for this research was provided by the National Institutes of Health (NIMH R01MH118203, NIMH U01MH121260, NIMH R01MH096875, NIA R01AG060931, NIA R00AG051764, and NIA R01AG084706) and the National Science Foundation (BCS 1800558 and BCS 1752393). The Caribbean Primate Research Center is supported by the National Institutes of Health (NCRR/ORIP P40OD012217). A.R.D.'s past and current support includes the New York University MacCracken Fellowship, a Graduate Research Fellowship from the National Science Foundation (DGE1342536), and a Postdoctoral Intramural Research Award from the National Institute of Mental Health. K.L.C. was and is supported by National Institutes of Health fellowships (NIA T32AG000057 and NIA K99AG075241).

AUTHOR CONTRIBUTIONS

A.R.D., J.P.H., C.C.S., K.L.C., and N.S.-M. conceptualized the research. M.J.M., N.S.-M., K.L.C., O.G., and S.E.B.S., facilitated by M.I.M., A.V.R.-L., J.S., C.S.W., S.C.A., M.K.S., J.P.H., M.L.P., and C.B.R.U., collected brain tissue. A.R.D. and C.C.S. dissected the brain tissue. M.A.W. contributed data. A.R.D., K.L.C., and A.M. performed genomic lab work. J.E.N.-D.V. collected behavioral data using a protocol designed by L.J.N.B. A.R.D. and K.L.C. performed genomic analysis with input from N.S.-M. C.T. and L.J.N.B. performed behavioral analysis. A.R.D., K.L.C., J.P.H., and N.S.-M. wrote the manuscript. All authors reviewed and revised the manuscript.

DECLARATION OF INTERESTS

The authors declare no competing interests.

Received: June 15, 2023
Revised: December 28, 2023
Accepted: May 31, 2024
Published: June 27, 2024

REFERENCES

1. Eid, R.S., Gobinath, A.R., and Galea, L.A.M. (2019). Sex differences in depression: Insights from clinical and preclinical studies. Prog. Neurobiol. *176*, 86–102. https://doi.org/10.1016/j.pneurobio.2019.01.006.

2. Hebert, L.E., Weuve, J., Scherr, P.A., and Evans, D.A. (2013). Alzheimer disease in the United States (2010-2050) estimated using the 2010 census. Neurology *80*, 1778–1783. https://doi.org/10.1212/WNL.0b013e31828726f5.

3. Bálint, S., Czobor, P., Komlósi, S., Mészáros, A., Simon, V., and Bitter, I. (2009). Attention deficit hyperactivity disorder (ADHD): gender-and age-related differences in neurocognition. Psychol. Med. *39*, 1337–1345. https://doi.org/10.1017/S0033291708004236.

4. Ferri, S.L., Abel, T., and Brodkin, E.S. (2018). Sex differences in autism spectrum disorder: A review. Curr. Psychiatry Rep. *20*, 9. https://doi.org/10.1007/s11920-018-0874-2.

5. Wooten, G.F., Currie, L.J., Bovbjerg, V.E., Lee, J.K., and Patrie, J. (2004). Are men at greater risk for Parkinson's disease than women? J. Neurol. Neurosurg. Psychiatry *75*, 637–639. https://doi.org/10.1136/jnnp.2003.020982.

6. Hull, L., Petrides, K.V., and Mandy, W. (2020). The female autism phenotype and camouflaging: A narrative review. Rev. J. Autism Dev. Disord. *7*, 306–317. https://doi.org/10.1007/s40489-020-00197-9.

7. Santos, S., Ferreira, H., Martins, J., Gonçalves, J., and Castelo-Branco, M. (2022). Male sex bias in early and late onset neurodevelopmental disorders: Shared aspects and differences in Autism Spectrum Disorder, Attention Deficit/hyperactivity Disorder, and Schizophrenia. Neurosci. Biobehav. Rev. *135*, 104577. https://doi.org/10.1016/j.neubiorev.2022.104577.

8. Pinares-Garcia, P., Stratikopoulos, M., Zagato, A., Loke, H., and Lee, J. (2018). Sex: A Significant Risk Factor for Neurodevelopmental and Neurodegenerative Disorders. Brain Sci. *8*, 154. https://doi.org/10.3390/brainsci8080154.

9. Werling, D.M., Parikshak, N.N., and Geschwind, D.H. (2016). Gene expression in human brain implicates sexually dimorphic pathways in autism spectrum disorders. Nat. Commun. *7*, 10717. https://doi.org/10.1038/ncomms10717.

10. Oliva, M., Muñoz-Aguirre, M., Kim-Hellmuth, S., Wucher, V., Gewirtz, A.D.H., Cotter, D.J., Parsana, P., Kasela, S., Balliu, B., Viñuela, A., et al. (2020). The impact of sex on gene expression across human tissues. Science *369*, eaba3066. https://doi.org/10.1126/science.aba3066.

11. Lopes-Ramos, C.M., Chen, C.-Y., Kuijjer, M.L., Paulson, J.N., Sonawane, A.R., Fagny, M., Platig, J., Glass, K., Quackenbush, J., and DeMeo, D.L. (2020). Sex differences in gene expression and regulatory networks across 29 human tissues. Cell Rep. *31*, 107795. https://doi.org/10.1016/j.celrep.2020.107795.

12. Mayne, B.T., Bianco-Miotto, T., Buckberry, S., Breen, J., Clifton, V., Shoubridge, C., and Roberts, C.T. (2016). Large scale gene expression meta-analysis reveals tissue-specific, sex-biased gene expression in humans. Front. Genet. *7*, 183. https://doi.org/10.3389/fgene.2016.00183.

13. Shi, L., Zhang, Z., and Su, B. (2016). Sex biased gene expression profiling of human brains at major developmental stages. Sci. Rep. *6*, 21181–21189. https://doi.org/10.1038/srep21181.

14. Trabzuni, D., Ramasamy, A., Imran, S., Walker, R., Smith, C., Weale, M.E., Hardy, J., and Ryten, M.; North American Brain Expression Consortium (2013). Widespread sex differences in gene expression and splicing in the adult human brain. Nat. Commun. *4*, 2771. https://doi.org/10.1038/ncomms3771.

15. Kang, H.J., Kawasawa, Y.I., Cheng, F., Zhu, Y., Xu, X., Li, M., Sousa, A.M.M., Pletikos, M., Meyer, K.A., Sedmak, G., et al. (2011). Spatio-temporal transcriptome of the human brain. Nature *478*, 483–489. https://doi.org/10.1038/nature10523.

16. Ziats, M.N., and Rennert, O.M. (2013). Sex-biased gene expression in the developing brain: implications for autism spectrum disorders. Mol. Autism. *4*, 10. https://doi.org/10.1186/2040-2392-4-10.

17. Ziats, M.N., and Rennert, O.M. (2014). Identification of differentially expressed microRNAs across the developing human brain. Mol. Psychiatry *19*, 848–852. https://doi.org/10.1038/mp.2013.93.

18. Preuss, T.M. (1995). Do Rats Have Prefrontal Cortex? The Rose-Woolsey-Akert Program Reconsidered. J. Cogn. Neurosci. *7*, 1–24. https://doi.org/10.1162/jocn.1995.7.1.1.

19. Watson, K.K., and Platt, M.L. (2012). Of mice and monkeys: using non-human primate models to bridge mouse- and human-based investigations of autism spectrum disorders. J. Neurodev. Disord. *4*, 21. https://doi.org/10.1186/1866-1955-4-21.

20. Li, H.W., Zhang, L., and Qin, C. (2019). Current state of research on non-human primate models of Alzheimer's disease. Anim. Models Exp. Med. *2*, 227–238. https://doi.org/10.1002/ame2.12092.

21. Darwin, C.R. (1871). The Descent of Man and Selection in Relation to Sex (Murray).

22. Naqvi, S., Godfrey, A.K., Hughes, J.F., Goodheart, M.L., Mitchell, R.N., and Page, D.C. (2019). Conservation, acquisition, and functional impact of sex-biased gene expression in mammals. Science *365*, eaaw7317. https://doi.org/10.1126/science.aaw7317.

23. Cutter, A.D., and Ward, S. (2005). Sexual and temporal dynamics of molecular evolution in *C. elegans* development. Mol. Biol. Evol. *22*, 178–188. https://doi.org/10.1093/molbev/msh267.

24. Good, J.M., and Nachman, M.W. (2005). Rates of protein evolution are positively correlated with developmental timing of expression during mouse spermatogenesis. Mol. Biol. Evol. *22*, 1044–1052. https://doi.org/10.1093/molbev/msi087.

25. Harrison, P.W., Wright, A.E., Zimmer, F., Dean, R., Montgomery, S.H., Pointer, M.A., and Mank, J.E. (2015). Sexual selection drives evolution and rapid turnover of male gene expression. Proc. Natl. Acad. Sci. USA *112*, 4393–4398. https://doi.org/10.1073/pnas.1501339112.

26. Pröschel, M., Zhang, Z., and Parsch, J. (2006). Widespread adaptive evolution of *Drosophila* genes with sex-biased expression. Genetics *174*, 893–900. https://doi.org/10.1534/genetics.106.058008.

27. Ranz, J.M., Castillo-Davis, C.I., Meiklejohn, C.D., and Hartl, D.L. (2003). Sex-dependent gene expression and evolution of the *Drosophila* transcriptome. Science *300*, 1742–1745. https://doi.org/10.1126/science.1085881.

28. Torgerson, D.G., Kulathinal, R.J., and Singh, R.S. (2002). Mammalian sperm proteins are rapidly evolving: Evidence of positive selection in functionally diverse genes. Mol. Biol. Evol. *19*, 1973–1980. https://doi.org/10.1093/oxfordjournals.molbev.a004021.

29. Zhang, Z., Hambuch, T.M., and Parsch, J. (2004). Molecular evolution of sex-biased genes in *Drosophila*. Mol. Biol. Evol. *21*, 2130–2139. https://doi.org/10.1093/molbev/msh223.

30. Bakken, T.E., Miller, J.A., Ding, S.-L., Sunkin, S.M., Smith, K.A., Ng, L., Szafer, A., Dalley, R.A., Royall, J.J., Lemon, T., et al. (2016). A comprehensive transcriptional map of primate brain development. Nature *535*, 367–375. https://doi.org/10.1038/nature18637.

31. Kojima, T., Higo, N., Sato, A., Oishi, T., Nishimura, Y., Yamamoto, T., Murata, Y., Yoshino-Saito, K., Onoe, H., and Isa, T. (2013). Functional annotation of genes differentially expressed between primary motor and prefrontal association cortices of macaque brain. Neurochem. Res. *38*, 133–140. https://doi.org/10.1007/s11064-012-0900-4.

32. Liu, S., Wang, Z., Chen, D., Zhang, B., Tian, R.-R., Wu, J., Zhang, Y., Xu, K., Yang, L.-M., Cheng, C., et al. (2017). Annotation and cluster analysis of spatiotemporal- and sex-related lncRNA expression in rhesus macaque brain. Genome Res. *27*, 1608–1620. https://doi.org/10.1101/gr.217463.116.

33. Yin, S., Lu, K., Tan, T., Tang, J., Wei, J., Liu, X., Hu, X., Wan, H., Huang, W., Fan, Y., et al. (2020). Transcriptomic and open chromatin atlas of high-resolution anatomical regions in the rhesus macaque brain. Nat. Commun. *11*, 474. https://doi.org/10.1038/s41467-020-14368-z.

34. Zhu, Y., Sousa, A.M.M., Gao, T., Skarica, M., Li, M., Santpere, G., Esteller-Cucala, P., Juan, D., Ferrández-Peral, L., Gulden, F.O., et al. (2018). Spatiotemporal transcriptomic divergence across human and macaque brain development. Science *362*, eaat8077. https://doi.org/10.1126/science.aat8077.

35. Chiou, K.L., DeCasien, A.R., Rees, K.P., Testard, C., Spurrell, C.H., Gogate, A.A., Pliner, H.A., Tremblay, S., Mercer, A., Whalen, C.J., et al. (2022). Multiregion transcriptomic profiling of the primate brain reveals signatures of aging and the social environment. Nat. Neurosci. *25*, 1714–1723. https://doi.org/10.1038/s41593-022-01197-0.

36. Akdemir, D., and Okeke, U. (2015). EMMREML: Fitting mixed models with known covariance structures.

37. Sallet, J., Mars, R.B., Noonan, M.P., Andersson, J.L., O'Reilly, J.X., Jbabdi, S., Croxson, P.L., Jenkinson, M., Miller, K.L., and Rushworth, M.F.S. (2011). Social network size affects neural circuits in macaques. Science *334*, 697–700. https://doi.org/10.1126/science.1210027.

38. Noonan, M.P., Sallet, J., Mars, R.B., Neubert, F.X., O'Reilly, J.X., Andersson, J.L., Mitchell, A.S., Bell, A.H., Miller, K.L., and Rushworth, M.F.S. (2014). A Neural Circuit Covarying with Social Hierarchy in Macaques. PLoS Biol. *12*, e1001940. https://doi.org/10.1371/journal.pbio.1001940.

39. Cooper, E.B., Brent, L.J.N., Snyder-Mackler, N., Singh, M., Sengupta, A., Khatiwada, S., Malaivijitnond, S., Qi Hai, Z., and Higham, J.P. (2022). The rhesus macaque as a success story of the Anthropocene. eLife *11*, e78169. https://doi.org/10.7554/eLife.78169.

40. Stephens, M. (2017). False discovery rates: a new deal. Biostatistics *18*, 275–294. https://doi.org/10.1093/biostatistics/kxw041.

CellPress
OPEN ACCESS

41. Balaton, B.P., Fornes, O., Wasserman, W.W., and Brown, C.J. (2021). Cross-species examination of X-chromosome inactivation highlights domains of escape from silencing. Epigenet. Chromatin *14*, 12. https://doi.org/10.1186/s13072-021-00386-8.

42. Ma, W., Bonora, G., Berletch, J.B., Deng, X., Noble, W.S., and Disteche, C.M. (2018). X-Chromosome Inactivation and Escape from X Inactivation in Mouse. In X-Chromosome Inactivation Methods in Molecular Biology, T. Sado, ed. (Springer), pp. 205–219. https://doi.org/10.1007/978-1-4939-8766-5_15.

43. Zhang, Y., Sturgill, D., Parisi, M., Kumar, S., and Oliver, B. (2007). Constraint and turnover in sex-biased gene expression in the genus Drosophila. Nature *450*, 233–237. https://doi.org/10.1038/nature06323.

44. McKenzie, A.T., Wang, M., Hauberg, M.E., Fullard, J.F., Kozlenkov, A., Keenan, A., Hurd, Y.L., Dracheva, S., Casaccia, P., Roussos, P., and Zhang, B. (2018). Brain cell type specific gene expression and co-expression network architectures. Sci. Rep. *8*, 8868. https://doi.org/10.1038/s41598-021-97284-6.

45. Fass, S.B., Mulvey, B., Chase, R., Yang, W., Selmanovic, D., Chaturvedi, S., Tycksen, E., Weiss, L.A., and Dougherty, J.D. (2024). Relationship between sex biases in gene expression and sex biases in autism and Alzheimer's disease. Biol. Sex Differ. *15*, 47. https://doi.org/10.1186/s13293-024-00622-2.

46. Li, M., Santpere, G., Imamura Kawasawa, Y., Evgrafov, O.V., Gulden, F.O., Pochareddy, S., Sunkin, S.M., Li, Z., Shin, Y., Zhu, Y., et al. (2018). Integrative functional genomic analysis of human brain development and neuropsychiatric risks. Science *362*, eaat7615. https://doi.org/10.1126/science.aat7615.

47. Pelvig, D.P., Pakkenberg, H., Stark, A.K., and Pakkenberg, B. (2008). Neocortical glial cell numbers in human brains. Neurobiol. Aging *29*, 1754–1762. https://doi.org/10.1016/j.neurobiolaging.2007.04.013.

48. Natri, H., Garcia, A.R., Buetow, K.H., Trumble, B.C., and Wilson, M.A. (2019). The pregnancy pickle: Evolved immune compensation due to pregnancy underlies sex differences in human diseases. Trends Genet. *35*, 478–488. https://doi.org/10.1016/j.tig.2019.04.008.

49. Hanamsagar, R., Alter, M.D., Block, C.S., Sullivan, H., Bolton, J.L., and Bilbo, S.D. (2017). Generation of a microglial developmental index in mice and in humans reveals a sex difference in maturation and immune reactivity. Glia *65*, 1504–1520. https://doi.org/10.1002/glia.23176.

50. Lenz, K.M., and McCarthy, M.M. (2015). A Starring Role for Microglia in Brain Sex Differences. Neuroscientist *21*, 306–321. https://doi.org/10.1177/1073858414536468.

51. Gober, R., Ardalan, M., Shiadeh, S.M.J., Duque, L., Garamszegi, S.P., Ascona, M., Barreda, A., Sun, X., Mallard, C., and Vontell, R.T. (2022). Microglia activation in postmortem brains with schizophrenia demonstrates distinct morphological changes between brain regions. Brain Pathol. *32*, e13003. https://doi.org/10.1111/bpa.13003.

52. Griffin, A., Chen, M., and Tiwari, V.K. (2023). Dissection of cellular disruptions in autism spectrum disorder comorbidities. Eur. J. Neurosci. *58*, 3921–3931. https://doi.org/10.1111/ejn.16155.

53. Morgan, J.T., Chana, G., Pardo, C.A., Achim, C., Semendeferi, K., Buckwalter, J., Courchesne, E., and Everall, I.P. (2010). Microglial activation and increased microglial density observed in the dorsolateral prefrontal cortex in autism. Biol. Psychiatry *68*, 368–376. https://doi.org/10.1016/j.biopsych.2010.05.024.

54. Oliveira-Pinto, A.V., Andrade-Moraes, C.H., Oliveira, L.M., Parente-Bruno, D.R., Santos, R.M., Coutinho, R.A., Alho, A.T.L., Leite, R.E.P., Suemoto, C.K., Grinberg, L.T., et al. (2016). Do age and sex impact on the absolute cell numbers of human brain regions? Brain Struct. Funct. *221*, 3547–3559. https://doi.org/10.1007/s00429-015-1118-4.

55. Rabinowicz, T., Petetot, J.M.-C., Gartside, P.S., Sheyn, D., Sheyn, T., and de, C.M. (2002). Structure of the Cerebral Cortex in Men and Women. J. Neuropathol. Exp. Neurol. *61*, 46–57. https://doi.org/10.1093/jnen/61.1.46.

56. Bauernfeind, A.L., and Babbitt, C.C. (2020). Metabolic changes in human brain evolution. Evol. Anthropol. *29*, 201–211. https://doi.org/10.1002/evan.21831.

57. Alonso-Nanclares, L., Gonzalez-Soriano, J., Rodriguez, J.R., and DeFelipe, J. (2008). Gender differences in human cortical synaptic density. Proc. Natl. Acad. Sci. USA *105*, 14615–14619. https://doi.org/10.1073/pnas.0803652105.

58. Nilsson, S., Mäkelä, S., Treuter, E., Tujague, M., Thomsen, J., Andersson, G., Enmark, E., Pettersson, K., Warner, M., and Gustafsson, J.-Å. (2001). Mechanisms of Estrogen Action. Physiol. Rev. *81*, 1535–1565. https://doi.org/10.1152/physrev.2001.81.4.1535.

59. Gandal, M.J., Haney, J.R., Wamsley, B., Yap, C.X., Parhami, S., Emani, P.S., Chang, N., Chen, G.T., Hoftman, G.D., de Alba, D., et al. (2022). Broad transcriptomic dysregulation occurs across the cerebral cortex in ASD. Nature *611*, 532–539. https://doi.org/10.1038/s41586-022-05377-7.

60. Voineagu, I., Wang, X., Johnston, P., Lowe, J.K., Tian, Y., Horvath, S., Mill, J., Cantor, R.M., Blencowe, B.J., and Geschwind, D.H. (2011). Transcriptomic analysis of autistic brain reveals convergent molecular pathology. Nature *474*, 380–384. https://doi.org/10.1038/nature10110.

61. Gupta, S., Ellis, S.E., Ashar, F.N., Moes, A., Bader, J.S., Zhan, J., West, A.B., and Arking, D.E. (2014). Transcriptome analysis reveals dysregulation of innate immune response genes and neuronal activity-dependent genes in autism. Nat. Commun. *5*, 5748. https://doi.org/10.1038/ncomms6748.

62. Satterstrom, F.K., Kosmicki, J.A., Wang, J., Breen, M.S., De Rubeis, S., An, J.-Y., Peng, M., Collins, R., Grove, J., Klei, L., et al. (2020). Large-Scale Exome Sequencing Study Implicates Both Developmental and Functional Changes in the Neurobiology of Autism. Cell *180*, 568–584.e23. https://doi.org/10.1016/j.cell.2019.12.036.

63. Gandal, M.J., Haney, J.R., Parikshak, N.N., Leppa, V., Ramaswami, G., Hartl, C., Schork, A.J., Appadurai, V., Buil, A., Werge, T.M., et al. (2018). Shared molecular neuropathology across major psychiatric disorders parallels polygenic overlap. Science *359*, 693–697. https://doi.org/10.1126/science.aad6469.

64. Ruzzo, E.K., Pérez-Cano, L., Jung, J.-Y., Wang, L.K., Kashef-Haghighi, D., Hartl, C., Singh, C., Xu, J., Hoekstra, J.N., Leventhal, O., et al. (2019). Inherited and de novo genetic risk for autism impacts shared networks. Cell *178*, 850–866.e26. https://doi.org/10.1016/j.cell.2019.07.015.

65. Parikshak, N.N., Swarup, V., Belgard, T.G., Irimia, M., Ramaswami, G., Gandal, M.J., Hartl, C., Leppa, V., Ubieta, L.d.l.T., Huang, J., et al. (2016). Genome-wide changes in lncRNA, splicing, and regional gene expression patterns in autism. Nature *540*, 423–427. https://doi.org/10.1038/nature20612.

66. Yerbury, J.J., Ooi, L., Dillin, A., Saunders, D.N., Hatters, D.M., Beart, P.M., Cashman, N.R., Wilson, M.R., and Ecroyd, H. (2016). Walking the tightrope: proteostasis and neurodegenerative disease. J. Neurochem. *137*, 489–505. https://doi.org/10.1111/jnc.13575.

67. Jauny, G., Eustache, F., and Hinault, T. (2022). Connectivity dynamics and cognitive variability during aging. Neurobiol. Aging *118*, 99–105. https://doi.org/10.1016/j.neurobiolaging.2022.07.001.

68. Ellegren, H., and Parsch, J. (2007). The evolution of sex-biased genes and sex-biased gene expression. Nat. Rev. Genet. *8*, 689–698. https://doi.org/10.1038/nrg2167.

69. Vicoso, B., and Charlesworth, B. (2009). Effective population size and the faster-X effect: an extended model. Evolution *63*, 2413–2426. https://doi.org/10.1111/j.1558-5646.2009.00719.x.

70. Mank, J.E., Hultin-Rosenberg, L., Zwahlen, M., and Ellegren, H. (2008). Pleiotropic constraint hampers the resolution of sexual antagonism in vertebrate gene expression. Am. Nat. *171*, 35–43. https://doi.org/10.1086/523954.

71. Allen, S.L., Bonduriansky, R., and Chenoweth, S.F. (2018). Genetic constraints on microevolutionary divergence of sex-biased gene expression. Philos. Trans. R. Soc. Lond. B Biol. Sci. *373*, 20170427. https://doi.org/10.1098/rstb.2017.0427.

72. Lande, R. (1980). Genetic variation and phenotypic evolution during allopatric speciation. Am. Nat. *116*, 463–479. https://doi.org/10.1086/283642.

73. Fisher, R.A. (1958). The Genetical Theory of Natural Selection, 2nd edition (Dover Publications).

74. Bakken, T.E., Hodge, R.D., Miller, J.A., Yao, Z., Nguyen, T.N., Aevermann, B., Barkan, E., Bertagnolli, D., Casper, T., Dee, N., et al. (2018). Single-nucleus and single-cell transcriptomes compared in matched cortical cell types. PLoS One *13*, e0209648. https://doi.org/10.1371/journal.pone.0209648.

75. Kissel, L.T., and Werling, D.M. (2022). Neural Transcriptomic Analysis of Sex Differences in Autism Spectrum Disorder: Current Insights and Future Directions. Biol. Psychiatry *91*, 53–60. https://doi.org/10.1016/j.biopsych.2020.11.023.

76. Schwarz, J.M., Sholar, P.W., and Bilbo, S.D. (2012). Sex differences in microglial colonization of the developing rat brain: Sex differences in microglial colonization. J. Neurochem. *120*, 948–963. https://doi.org/10.1111/j.1471-4159.2011.07630.x.

77. DeCasien, A.R., Guma, E., Liu, S., and Raznahan, A. (2022). Sex differences in the human brain: a roadmap for more careful analysis and interpretation of a biological reality. Bio. Sex Differ. *13*, 43. https://doi.org/10.1186/s13293-022-00448-w.

78. Rippon, G., Eliot, L., Genon, S., and Joel, D. (2021). How hype and hyperbole distort the neuroscience of sex differences. PLoS Biol. *19*, e3001253. https://doi.org/10.1371/journal.pbio.3001253.

79. Testard, C., Brent, L.J.N., Andersson, J., Chiou, K.L., Negron-Del Valle, J.E., DeCasien, A.R., Acevedo-Ithier, A., Stock, M.K., Antón, S.C., Gonzalez, O., et al. (2022). Social connections predict brain structure in a multidimensional free-ranging primate society. Sci. Adv. *8*, eabl5794. https://doi.org/10.1126/sciadv.abl5794.

80. Saleem, K.S., and Logothetis, N.K. (2012). A Combined MRI and Histology Atlas of the Rhesus Monkey Brain in Stereotaxic Coordinates (Academic Press).

81. He, Z., Han, D., Efimova, O., Guijarro, P., Yu, Q., Oleksiak, A., Jiang, S., Anokhin, K., Velichkovsky, B., Grünewald, S., and Khaitovich, P. (2017). Comprehensive transcriptome analysis of neocortical layers in humans, chimpanzees and macaques. Nat. Neurosci. *20*, 886–895. https://doi.org/10.1038/nn.4548.

82. Keuker, J.I.H., Luiten, P.G.M., and Fuchs, E. (2003). Preservation of hippocampal neuron numbers in aged rhesus monkeys. Neurobiol. Aging *24*, 157–165.

83. Wong, K.S., and Pang, H.m. (2013). Simplifying HT RNA Quality & Quantity Analysis. Genetic Engineering & Biotechnology News *33*, 17. https://doi.org/10.1016/s0197-4580(02)00062-3.

84. Auer, H., Lyianarachchi, S., Newsom, D., Klisovic, M.I., Marcucci, G., and Kornacker, K. (2003). Chipping away at the chip bias: RNA degradation in microarray analysis. Nat. Genet. *35*, 292–293. https://doi.org/10.1038/ng1203-292.

85. Bray, N.L., Pimentel, H., Melsted, P., and Pachter, L. (2016). Near-optimal probabilistic RNA-seq quantification. Nat. Biotechnol. *34*, 525–527. https://doi.org/10.1038/nbt.3519.

86. Webster, T.H., Couse, M., Grande, B.M., Karlins, E., Phung, T.N., Richmond, P.A., Whitford, W., and Wilson, M.A. (2019). Identifying, understanding, and correcting technical artifacts on the sex chromosomes in next-generation sequencing data. GigaScience *8*, giz074.

87. Hughes, J.F., Skaletsky, H., Brown, L.G., Pyntikova, T., Graves, T., Fulton, R.S., Dugan, S., Ding, Y., Buhay, C.J., Kremitzki, C., et al. (2012). Strict evolutionary conservation followed rapid gene loss on human and rhesus Y chromosomes. Nature *483*, 82–86. https://doi.org/10.1038/nature10843.

88. Robinson, M.D., McCarthy, D.J., and Smyth, G.K. (2010). edgeR: a Bioconductor package for differential expression analysis of digital gene expression data. Bioinformatics *26*, 139–140. https://doi.org/10.1093/gigascience/giz074.

89. Ritchie, M.E., Phipson, B., Wu, D., Hu, Y., Law, C.W., Shi, W., and Smyth, G.K. (2015). limma powers differential expression analyses for RNA-sequencing and microarray studies. Nucleic Acids Res. *43*, e47. https://doi.org/10.1093/nar/gkv007.

90. Dobin, A., Davis, C.A., Schlesinger, F., Drenkow, J., Zaleski, C., Jha, S., Batut, P., Chaisson, M., and Gingeras, T.R. (2013). STAR: ultrafast universal RNA-seq aligner. Bioinformatics *29*, 15–21. https://doi.org/10.1093/bioinformatics/bts635.

91. Danecek, P., Bonfield, J.K., Liddle, J., Marshall, J., Ohan, V., Pollard, M.O., Whitwham, A., Keane, T., McCarthy, S.A., Davies, R.M., and Li, H. (2021). Twelve years of SAMtools and BCFtools. GigaScience *10*, giab008. https://doi.org/10.1093/gigascience/giab008.

92. McKenna, A., Hanna, M., Banks, E., Sivachenko, A., Cibulskis, K., Kernytsky, A., Garimella, K., Altshuler, D., Gabriel, S., Daly, M., and DePristo, M.A. (2010). The Genome Analysis Toolkit: A MapReduce framework for analyzing next-generation DNA sequencing data. Genome Res. *20*, 1297–1303. https://doi.org/10.1101/gr.107524.110.

93. Lipatov, M., Sanjeev, K., Patro, R., and Veeramah, K. (2015). Maximum Likelihood Estimation of Biological Relatedness from Low Coverage Sequencing Data. Preprint at bioRxiv. https://doi.org/10.1101/023374.

94. Danecek, P., Auton, A., Abecasis, G., Albers, C.A., Banks, E., DePristo, M.A., Handsaker, R.E., Lunter, G., Marth, G.T., Sherry, S.T., et al. (2011). The variant call format and VCFtools. Bioinformatics *27*, 2156–2158. https://doi.org/10.1093/bioinformatics/btr330.

95. Lea, A.J., Akinyi, M.Y., Nyakundi, R., Mareri, P., Nyundo, F., Kariuki, T., Alberts, S.C., Archie, E.A., and Tung, J. (2018). Dominance rank-associated gene expression is widespread, sex-specific, and a precursor to high social status in wild male baboons. Proc. Natl. Acad. Sci. USA *115*, E12163–E12171. https://doi.org/10.1073/pnas.1811967115.

96. Pallé, A., Zorzo, C., Luskey, V.E., Mcgreevy, K.R., Fernández, S., and Trejo, J.L. (2019). Social dominance differentially alters gene expression in the medial prefrontal cortex without affecting adult hippocampal neurogenesis or stress and anxiety-like behavior. FASEB J. *33*, 6995–7008. https://doi.org/10.1096/fj.201801600R.

97. So, N., Franks, B., Lim, S., and Curley, J.P. (2015). A Social Network Approach Reveals Associations between Mouse Social Dominance and Brain Gene Expression. PLoS One *10*, e0134509. https://doi.org/10.1371/journal.pone.0134509.

98. Testard, C., Larson, S.M., Watowich, M.M., Kaplinsky, C.H., Bernau, A., Faulder, M., Marshall, H.H., Lehmann, J., Ruiz-Lambides, A., Higham, J.P., et al. (2021). Rhesus macaques build new social connections after a natural disaster. Curr. Biol. *31*, 2299–2309.e7. https://doi.org/10.1016/j.cub.2021.03.029.

99. van Noordwijk, M., and Van Schaik, C.P. Sexual selection and the careers of primate males: paternity concentration, dominance acquisition-tactics and transfer decisions. In Sexual Selection in Primates: New and Comparative Perspectives (Cambridge University Press), pp. 208–229.

100. Madlon-Kay, S., Montague, M.J., Brent, L.J.N., Ellis, S., Zhong, B., Snyder-Mackler, N., Horvath, J.E., Skene, J.H.P., and Platt, M.L. (2018). Weak effects of common genetic variation in oxytocin and vasopressin receptor genes on rhesus macaque social behavior. Am. J. Primatol. *80*, e22873. https://doi.org/10.1002/ajp.22873.

101. R Core Team (2020). R: A Language and Environment for Statistical Computing (Foundation for Statistical Computing).

102. Heinz, S., Benner, C., Spann, N., Bertolino, E., Lin, Y.C., Laslo, P., Cheng, J.X., Murre, C., Singh, H., and Glass, C.K. (2010). Simple combinations of

CellPress
OPEN ACCESS

lineage-determining transcription factors prime cis-regulatory elements required for macrophage and B cell identities. Mol. Cell *38*, 576–589. https://doi.org/10.1016/j.molcel.2010.05.004.

103. Konopka, T. (2020). umap: Uniform Manifold Approximation and Projection.

104. Krijthe, J. (2015). Rtsne: T-Distributed Stochastic Neighbor Embedding Using a Barnes-Hut Implementation.

105. Suzuki, R., and Shimodaira, H. (2006). Pvclust: an R package for assessing the uncertainty in hierarchical clustering. Bioinformatics *22*, 1540–1542. https://doi.org/10.1093/bioinformatics/btl117.

106. Hoffman, G.E., and Schadt, E.E. (2016). variancePartition: interpreting drivers of variation in complex gene expression studies. BMC Bioinf. *17*, 483. https://doi.org/10.1186/s12859-016-1323-z.

107. Hoffman, G.E. (2020). Theory and Practice of Random Effects and REML in variancePartition and Dream.

108. Romero, I.G., Pai, A.A., Tung, J., and Gilad, Y. (2014). RNA-seq: impact of RNA degradation on transcript quantification. BMC Biol. *12*, 1–13. https://doi.org/10.1186/1741-7007-12-42.

109. Urbut, S.M., Wang, G., Carbonetto, P., and Stephens, M. (2019). Flexible statistical methods for estimating and testing effects in genomic studies with multiple conditions. Nat. Genet. *51*, 187–195. https://doi.org/10.1038/s41588-018-0268-8.

110. Stephens, M., Carbonetto, P., Dai, C., Gerard, D., Lu, M., Sun, L., Willwerscheid, J., Xiao, N., and Zeng, M. (2020). ashr: Methods for adaptive shrinkage, using Empirical Bayes.

111. Bovy, J., Hogg, D.W., and Roweis, S.T. (2011). Extreme deconvolution: Inferring complete distribution functions from noisy, heterogeneous and incomplete observations. Ann. Appl. Stat. *5*, 1657–1677. https://doi.org/10.1214/10-AOAS439.

112. Godfrey, A.K., Naqvi, S., Chmátal, L., Chick, J.M., Mitchell, R.N., Gygi, S.P., Skaletsky, H., and Page, D.C. (2020). Quantitative analysis of Y-Chromosome gene expression across 36 human tissues. Genome Res. *30*, 860–873. https://doi.org/10.1101/gr.261248.120.

113. Chen, B., Khodadoust, M.S., Liu, C.L., Newman, A.M., and Alizadeh, A.A. (2018). Profiling tumor infiltrating immune cells with CIBERSORT. Methods Mol. Biol. *1711*, 243–259. https://doi.org/10.1007/978-1-4939-7493-1_12.

114. Chiou, K.L., Huang, X., Bohlen, M.O., Tremblay, S., DeCasien, A.R., O'Day, D.R., Spurrell, C.H., Gogate, A.A., Zintel, T.M., Cayo Biobank Research Unit, et al. (2023). A single-cell multi-omic atlas spanning the adult rhesus macaque brain. Sci. Adv. eadh1914. https://doi.org/10.1126/sciadv.adh1914.

115. Mendez, E.F., Wei, H., Hu, R., Stertz, L., Fries, G.R., Wu, X., Najera, K.E., Monterey, M.D., Lincoln, C.M., Kim, J.W., et al. (2021). Angiogenic gene networks are dysregulated in opioid use disorder: evidence from multi-omics and imaging of postmortem human brain. Mol. Psychiatry *26*, 7803–7812. https://doi.org/10.1038/s41380-021-01259-y.

116. Narendra, S., Klengel, C., Hamzeh, B., Patel, D., Otten, J., Lardenoije, R., Newman, E.L., Miczek, K.A., Klengel, T., Ressler, K.J., and Suh, J. (2022). Genome-wide transcriptomics of the amygdala reveals similar oligodendrocyte-related responses to acute and chronic alcohol drinking in female mice. Transl. Psychiatry *12*, 476. https://doi.org/10.1038/s41398-022-02231-2.

117. Alexa, A., and Rahnenfuhrer, J. (2010). topGO: enrichment analysis for gene ontology.

118. Brionne, A., Juanchich, A., and Hennequet-Antier, C. (2019). ViSEAGO: a Bioconductor package for clustering biological functions using Gene Ontology and semantic similarity. BioData Min. *12*, 16. https://doi.org/10.1186/s13040-019-0204-1.

119. Grossmann, S., Bauer, S., Robinson, P.N., and Vingron, M. (2007). Improved detection of overrepresentation of Gene-Ontology annotations with parent–child analysis. Bioinformatics *23*, 3024–3031. https://doi.org/10.1093/bioinformatics/btm440.

120. Wang, J.Z., Du, Z., Payattakool, R., Yu, P.S., and Chen, C.-F. (2007). A new method to measure the semantic similarity of GO terms. Bioinformatics *23*, 1274–1281. https://doi.org/10.1093/bioinformatics/btm087.

121. Ward, J.H. (1963). Hierarchical grouping to optimize an objective function. J. Am. Stat. Assoc. *58*, 236–244. https://doi.org/10.1080/01621459.1963.10500845.

122. Pletscher-Frankild, S., Pallejà, A., Tsafou, K., Binder, J.X., and Jensen, L.J. (2015). DISEASES: Text mining and data integration of disease–gene associations. Methods *74*, 83–89. https://doi.org/10.1016/j.ymeth.2014.11.020.

123. Korotkevich, G., Sukhov, V., Budin, N., Shpak, B., Artyomov, M.N., and Sergushichev, A. (2016). Fast gene set enrichment analysis. Preprint at bioRxiv. https://doi.org/10.1101/060012.

124. Kolberg, L., Raudvere, U., Kuzmin, I., Adler, P., Vilo, J., and Peterson, H. (2023). g:Profiler—interoperable web service for functional enrichment analysis and gene identifier mapping (2023 update). Nucleic Acids Res. *51*, W207–W212. https://doi.org/10.1093/nar/gkad347.

125. Friedman, J.H. (2001). Greedy function approximation: A gradient boosting machine. Ann. Statist. *29*, 1189–1232. https://doi.org/10.1214/aos/1013203451.

126. Garieri, M., Stamoulis, G., Blanc, X., Falconnet, E., Ribaux, P., Borel, C., Santoni, F., and Antonarakis, S.E. (2018). Extensive cellular heterogeneity of X inactivation revealed by single-cell allele-specific expression in human fibroblasts. Proc. Natl. Acad. Sci. USA *115*, 13015–13020. https://doi.org/10.1073/pnas.1806811115.

127. Ding, B., Gentleman, R., and Carey, V. (2012). bioDist: Different distance measures.

128. Yanai, I., Benjamin, H., Shmoish, M., Chalifa-Caspi, V., Shklar, M., Ophir, R., Bar-Even, A., Horn-Saban, S., Safran, M., Domany, E., et al. (2005). Genome-wide midrange transcription profiles reveal expression level relationships in human tissue specification. Bioinformatics *21*, 650–659. https://doi.org/10.1093/bioinformatics/bti042.

129. Fadista, J., Oskolkov, N., Hansson, O., and Groop, L. (2017). LoFtool: a gene intolerance score based on loss-of-function variants in 60 706 individuals. Bioinformatics *33*, 471–474. https://doi.org/10.1093/bioinformatics/btv602.

STAR★METHODS

KEY RESOURCES TABLE

REAGENT or RESOURCE	SOURCE	IDENTIFIER
Deposited data		
Analyzed macaque data	This paper	GEO: GSE180092
Analyzed human (GTEx v8) data	GTEx Consortium	https://gtexportal.org/home/
Software and algorithms		
All code to reproduce analyses presented in this work	This paper	https://github.com/ardecasien/cayo_brain_transcriptome_sex; https://doi.org/10.5281/zenodo.11068290
STAR (v2.5)	Dobin et al.[90]	https://github.com/alexdobin/STAR; RRID: SCR_004463
SAMtools (v1.9)	Danecek et al.[91]	https://www.htslib.org/; RRID: SCR_002105
GATK (v4.1.2.0)	McKenna et al.[92]	https://gatk.broadinstitute.org/hc/en-us; RRID: SCR_001876
VCFtools (v0.1.16)	Danecek et al.[94]	https://vcftools.github.io/; RRID: SCR_001235
lcMLkin (v20190218)	Lipatov et al.[93]	https://github.com/COMBINE-lab/maximum-likelihood-relatedness-estimation; RRID: SCR_025418
kallisto (v0.43.1)	Bray et al.[85]	https://pachterlab.github.io/kallisto/; RRID: SCR_016582
R (v4.0.0)	R Core[101]	https://cran.r-project.org/; RRID: SCR_001905
Homer (v4.10)	Heinz et al.[102]	http://homer.ucsd.edu/homer/; RRID: SCR_010881

RESOURCE AVAILABILITY

Lead contact

Requests should be directed to and will be fulfilled by the lead contact, Alex R. DeCasien (alex.decasien@gmail.com).

Materials availability

No materials were generated in this study.

Data and code availability

- RNA-seq data have been deposited in the Gene Expression Omnibus (GEO) and are publicly available as of the date of publication. Accession numbers are listed in the key resources table.
- All code has been deposited in a publicly available GitHub Repository and an unchanging archive of this repository was created in Zenodo. Links to both repositories are listed in the key resources table.
- Any additional information required to reanalyze the data reported in this paper is available from the lead contact upon request.

METHOD DETAILS

Tissue procurement and processing

All animals were adults from a single social group of the free-ranging, semi-provisioned colony on Cayo Santiago. They were removed from the island and humanely euthanized as part of the population management strategy implemented by the Caribbean Primate Research Center, University of Puerto Rico. No individuals were used for brain invasive procedures or had signs of malformations or lesions. Within 30 min of euthanasia, following perfusion with cold saline, whole brains were extracted. Left and right hemispheres were separated using a sterilized razor, and left hemispheres were set aside for fixation.[79] Right hemispheres were placed in a mold and cut into ½ centimeter coronal slabs. Slabs were flash frozen using an ethanol and dry ice mixture. Brains were stored at -80°C until dissection.

Tissue dissection

Brain samples were collected postmortem from 36 adult macaques (20 females, 16 males; Figure S1). Frozen slabs were kept on dry ice during sampling. Samples were collected using 1mm surgical punches with reference to coronal cross-sections from the rhesus macaque anatomical brain atlas.[80] This sampling method allowed us to sample relatively evenly across all cortical layers, which exhibit distinct cell composition and gene expression patterns.[81]

Fifteen brain regions of interest were identified on frozen hemispheres using gross landmarks (e.g., cortical sulci/gyri and white matter tracts). Specifically: 1) The ventromedial prefrontal cortex (vmPFC; areas 10m/32) was sampled from the rostral most plane when the cingulate, principal, and medio-orbital sulci were visible. Six punches were taken on the cingulate gyrus, from slightly inferior to the tip at the medial surface towards the underlying white matter in the latero-inferior direction; 2) The dlPFC (area 46d) was sampled from the rostral most plane when the cingulate, principal, and medio-orbital sulci were visible. Six punches were taken on gyrus superior to the principal sulcus, from the tip at the lateral surface towards the underlying white matter in the medio-inferior direction; 3) The vlPFC (area 12r) was samples from the rostral most plane when the cingulate, principal, and medio-orbital sulci were visible. Six punches were taken on gyrus inferior to the principal sulcus, from the tip at the lateral surface towards the underlying white matter in the medial direction; 4) The dmPFC (area 9m) was sampled from rostral most plane when the cingulate, principal, and medio-orbital sulci were visible. Six punches were taken on the superior frontal gyrus, from the tip at the medial surface towards the underlying white matter in the latero-inferior direction; 5) The anterior cingulate gyrus (ACCg; area 24) was sampled from the rostral most plane when the corpus callosum was visible. Six punches were taken on the cingulate gyrus, from the tip at the medial surface towards the underlying white matter in the latero-inferior direction; 6) The mid-superior temporal sulcus (mid-STS) was sampled when the central, intraparietal, lateral, and superior temporal sulci were visible. Six punches were taken from the inferior-most point of STS towards the superior white matter[37]; 7) The primary motor cortex (M1; area 4) was sampled when the precentral, central, lateral, and superior temporal sulci were visible. Six punches were taken from the superior-medial portion; 8) The primary visual cortex (V1; area 17) was sampled on the anterior surface of the most posterior slab, in the inferior arm of the calcarine sulcus; 9) The caudate nucleus (CN) was sampled at the rostral most point at which the internal capsule was visible and clearly separated the caudate nucleus from the putamen. Six punches were taken from the most superior-lateral point moving in the inferomedial direction; 10) The putamen (Pu) was sampled at the rostral most point at which the internal capsule was visible and clearly separated the caudate nucleus from the putamen. Six punches were taken from the most superior-lateral point moving in the inferomedial direction; 11) The amygdala was sampled when both it and optic chiasm were clearly visible. Seven punches were taken lateral to optic chiasm, sampling across the superior portion of the amygdaloid complex (representing the anterior cortical nucleus, central nucleus, medial nucleus, and superior portions of the accessory basal and basal nuclei); 12) The dentate gyrus (DG) was identified within the hippocampal formation by its slightly darker color, caused by a high density of small granule cells. This sampling also included CA4, which is difficult to differentiate from the polymorphic layer of the DG. Accordingly, other studies have combined these regions, collectively referring to them as the hilus (which is the formal name for the polymorphic layer of the DG)[82]; 13) The CA3 was sampled from the area superior to the DG within the hippocampal formation, in the medio-lateral direction. This sampling likely also included portions of CA2 and CA1; 14) The lateral geniculate nucleus of the thalamus (LGN) was sampled when it was clearly visible, and six punches were taken across all layers in the medio-inferior direction; 15) The ventromedial hypothalamus (VMH) was sampled from rostral most plane when the hypothalamus was visible. This sampling likely also included portions of surrounding nuclei (e.g., arcuate). Four to six punches were taken from the most medio-inferior portion. Dissected tissue samples were stored at -80°C prior to further processing.

RNA extraction

1 mL of Trizol was added to dissected frozen tissue samples immediately before lysing. A single chilled 5mm stainless steel bead was added to each tube before placing samples in the TissueLyser II bead mill. Samples were homogenized for 2 min at 20 hz. Plates were rotated before homogenization was repeated. Homogenized samples were then transferred to new tubes and incubated at room temperature for 5 min. 200 mL of chloroform was added to each sample, tubes were manually shaken for 15 s, incubated at room temperature for 2 min, and centrifuged at 12k at 4°C for 15 min. The upper aqueous solution (containing RNA) was transferred to a new sample tube, and Total RNA was extracted using Zymo *Quick*-RNA Microprep kits. Each sample was subjected to DNase treatment as per manufacturer's instructions.

RNA quality assessment

RNA quality was assessed using a Fragment Analyzer or a Tapestation, which provided RQN or RINe values, respectively. For later analyses, RQN and RINe values were converted to RIN values using published regression lines (RQN = 0.9697*RIN, R^2 = 0.9635; RINe = 0.991*RIN, R^2 = 0.936).[83,84]

Library preparation and sequencing

cDNA libraries were prepared using the NEBNext Ultra II RNA Library Prep Kit for Illumina, as per the manufacturer's instructions with some modifications. Briefly, poly-adenylated mRNA was purified from 200 ng of total RNA using the NEBNext Poly(A) mRNA Magnetic Isolation Module. The mRNA was then reverse transcribed into cDNA, ligated to Illumina adapters, size-selected for a median size of ~600 bp, and amplified via PCR for 12 cycles. Each sample was tagged with a unique molecular barcode and pooled samples into Illumina NovaSeq lanes (across 2 sequencing runs, one using 2x50bp sequencing on the S2 flow cell and another using 2x100bp sequencing on the S4 flow cell).

Reference genomes and read alignment

Following sequencing, we mapped reads to the rhesus macaque transcriptome v10 (Ensembl) using the pseudoaligner kallisto v0.43.1.[85] Given that sequence homology across the sex chromosomes present in reference genomes/transcriptome can lead to

technical mapping errors, we created two modified, sex-specific transcriptomes and separately mapped reads from males and females following Webster et al.[86] Specifically, the Y chromosome was removed from the female-specific transcriptome, and *CD99* on the Y chromosome (within the pseudoautosomal region[87]) was removed from the male-specific transcriptome.

We imported the transcript count matrices for males and females into R using the function *tximport* (R package *tximport*) and combined them into one count matrix. We summarized transcript counts to the gene level using the appropriate functions in the R package *biomaRt* and the function *summarizeToGene* (R package *tximport*). This procedure resulted in a 22514 x 532 (p x n) read-count matrix, where p is the number of genes measured and n is the number of samples. We confirmed the identity of all samples based on genotyping from the RNA-seq reads.

Quality assessment

We removed 5 samples that were low quality (e.g., samples with low Phred scores and/or high PCR duplication rates). This resulted in a 22514 x 527 (p x n) read-count matrix, where p is the number of genes measured and n is the number of samples. We also confirmed the chromosomal sex of all individuals/samples by mapping to an unedited (non-sex-specific transcriptome) and examining Y chromosome gene expression (Figure S17).

Read normalization

We normalized the read count matrix using the functions *calcNormFactors* (R package *edgeR*[88] and *voom* in the R package *limma*.[89] Prior to further RNA-seq data analysis, we filtered out genes that were very lowly or not detectably expressed in our samples. Specifically, within each region we removed any gene with mean TPM<10 in both males and females (i.e., genes with ≥10 mean TPM in at least one sex were retained). This procedure resulted in a mean of 10,171 genes (range: 9,617-11,135), and 12,672 unique genes were detectably expressed in at least one brain region. These data (normalized log2 counts per million reads) were used throughout the statistical analyses described below.

Genotyping

We used genotype data (with variants called from RNAseq data) to control for genetic relatedness among individuals in this study. For each sample, we mapped reads to the rhesus macaque reference genome v10 (Ensembl) using STAR[90] (and SAMtools[91]) and then pooled mapped reads for each individual across all brain regions. We used the Genome Analysis Toolkit (GATK)[92] to mark duplicates (MarkDuplicates), split reads spanning splice events (SplitNCigarReads), and recalibrate base quality scores (BaseRecalibrator and ApplyBQSR) before calling variants (HaplotypeCaller) using a standard minimum confidence threshold for calling of 20.0. We retained sites that passed the following filters: QD < 2.0; MQ < 40.0; FS > 60.0; HaplotypeScore >13.0; MQRankSum < −12.5; and ReadPosRankSum < −8.0. We estimated kinship with the program lcMLkin[93] using variants that were genotyped in all 36 individuals, had minor allele frequencies >0.3, minimum completeness of 0.9, and were at least 100 kb apart (thinned using VCFtools[94]). These relatedness estimates were confirmed using known mother-offspring pairs (5 known pairs: mean relatedness estimate = 0.48; remaining pairs: relatedness estimates ≤ 0.25)

Behavioral data collection

Previous work has shown that dominance rank can impact gene expression in the brain and peripheral tissues of wild and laboratory animals.[95–97] Here, dominance rank reflects the direction and outcome of win-loss agonistic interactions (e.g., aggression, threats, displacements, submissions) recorded during focal animal samples or during *ad libitum* observations. To calculate individual dominance ranks, behavioral data were collected for all animals in this study (and all other members of this social group age 4 and above) in the three months prior to removal. Methods for behavioral data collection as well as dominance rank inference in this population are described by Testard and colleagues.[79,98] Ranks were calculated separately within each sex because dominance is attained differently in male and female macaques. Specifically, male macaques tend to disperse from their natal groups and their rank in the new groups are largely determined by their duration of tenure.[99] Female macaques are philopatric and dominance rank is inherited maternally, resulting in stable linear dominance hierarchies among females.[79,98] Accordingly, known maternal relatedness was used to resolve behavioral gaps in the female hierarchy. To account for group size, dominance rank was first defined as the percentage of same sex individuals that a subject outranked. We then followed previous work[100] in creating categorical dominance ranks, calculated by classifying animals as high- (rank ≥ 80%), mid- (50% ≤ rank < 80%), or low-ranking (rank < 50%) based on their percentage dominance ranks within each sex. We modeled categorical dominance rank as an ordinal variable for all differential expression analyses using the ordered factor class in R.

QUANTIFICATION AND STATISTICAL ANALYSIS

All statistical analyses were performed using R v4.0.0[101] or Homer v4.10.[102]

Dimensionality reduction

To visualize the structure of the expression data, we applied dimension reduction methods to the normalized, filtered expression matrix. Prior to dimension reduction, the effects of library batch and RIN were removed from the data using the *removeBatchEffect*

function in the R package *limma*.[89] Dimension reduction was performed using Uniform Manifold Approximation and Projection (UMAP) via the *umap* function in the R package *umap*[103] with the following metrics: n_neighbors = 200, min_dist = 0.5, metric = 'manhattan'. We also provide t-SNE and PCA plots in the supplement using the *Rtsne* function (perplexity = 30) in the R package *Rtsne*[104] and the *prcomp* function in the R package *stats*).

Hierarchical clustering

Unsupervised hierarchical clustering was conducted using the normalized, filtered expression matrix. Prior to hierarchical clustering, the effects of library batch and RIN were removed from the data using the *removeBatchEffect* function in the R package *limma*.[89] Cluster analyses were performed by the *pvclust* function (R package *pvclust*).[105] Correlation was used as the distance measure. This function provides both approximately unbiased (AU) *p* value and bootstrap probability (BP) value. AU values are calculated using multiscale bootstrap resampling, while BP values are calculated by the ordinary bootstrap resampling.[105] This method was applied to expression values averaged across samples per region (to examine clustering by region).

Variance partitioning

We performed variance partitioning on the normalized, filtered expression matrix using the *fitExtractVarPartModel* and *plotVarPart* functions in the R package *variancePartition*.[106] This function allowed us to fit a linear mixed model to estimate contribution of multiple sources of variation while simultaneously correcting for all other variables. Prior to hierarchical clustering, the effects of library batch and RIN were removed from the data using the *removeBatchEffect* function R package *limma*.[89] We modeled the expression of each gene as a function of individual, region, sex, age, and ordinal rank. Categorical terms were modeled as random effects, as recommended by the package's creator.[107] We then extracted and visualized the fraction of variance explained by each biological or demographic term, in addition to the residual variance.

Modeling sex effects on gene expression

To identify genes that were affected by sex within each region, we used linear mixed effects models that control for relatedness. We analyzed each of the 15 brain regions separately using the *emmreml* function in the R package *EMMREML*.[36] Normalized gene expression values were modeled as a function of sex, age, ordinal rank, RIN, and library batch. Although standard normalizations fail to account for the effects of RNA degradation, statistically controlling for RNA quality corrects for most of these effects.[108] For each gene in the normalized, filtered expression matrix, we estimated the effect of sex on gene expression using the Equation 1 below:

Y = intercept + sex + age + ordinal rank + RIN + library batch

$$y = \boldsymbol{\mu} + \boldsymbol{\nu}\boldsymbol{\beta} + a\boldsymbol{\gamma} + r\boldsymbol{\delta} + r^2\boldsymbol{\delta}^2 + \boldsymbol{\rho}\partial + \omega_{1-k}\tau_{1-k} + Zu + \boldsymbol{\varepsilon}, u \sim MVN\left(0, \boldsymbol{\sigma}_u^2 K\right)\boldsymbol{\varepsilon} \sim MVN\left(0, \boldsymbol{\sigma}_e^2 I\right) \quad \text{(Equation 1)}$$

where *y* is the *n* by 1 vector of normalized gene expression levels for the *n* samples collected per region; μ is the intercept; $\boldsymbol{\nu}$ is an *n* by 1 vector of sex and β is its effect size; *a* is an *n* by 1 vector of age in years at the time of sample collection and γ is its effect size; *r* is an *n* by 1 vector of linear contrasts of sex-specific rank and $\boldsymbol{\delta}$ is its effect size; r^2 is an *n* by 1 vector of quadratic contrasts of sex-specific rank and $\boldsymbol{\delta}^2$ is its effect size; ρ is an *n* by 1 vector of RIN values and ∂ is its effect size; and $\omega_{1\text{-}k}$ are *k* vectors (with *k* equal to the number of library batches for the given region), each of which is an *n* by 1 vector of a dummy variable for that library batch (0 = sample not included in this batch; 1 = sample included in this batch), and $\tau_{1\text{-}k}$ are the effect sizes for each vector. The *m* by 1 vector *u* is a random effects term to control for kinship and other sources of genetic structure. Here, *m* is the number of unique individuals sampled for each region, the *m* by *m* matrix *K* contains estimates of pairwise a relatedness derived from a genotype data set, $\boldsymbol{\sigma}_u{}^2$ is the genetic variance component (0 for a non-heritable trait), and *Z* is an incidence matrix of 1's and 0's that maps samples to individuals in the random effects term. Residual errors are represented by ε, an *n* by 1 vector, where $\boldsymbol{\sigma}_e{}^2$ represents the environmental variance component (unstructured by genetic relatedness), *I* is the identity matrix, and *MVN* denotes the multivariate normal distribution.

Multivariate adaptive shrinkage (MASH)

To identify genes that are differentially expressed between males and females and whether or not these effects are shared or region-specific sex effects, we used the outputs from the EMMA mixed models described above (i.e., per gene βs and their standard errors within each of 15 regions) as inputs for multivariate adaptive shrinkage models (R package *mashr*).[109] For missing data, βs were set to 0 and standard errors were set to 100 (as recommended by the *mashr* package's creators). We first selected strong signals by running a condition-by-condition (1by1) analysis on all the data (*mash_1by1* function) and extracting those results with local false sign rate (LFSR) < 0.05 in any condition. Specifically, this analysis runs *ash* in the R package *ashr*[110] on the data from each condition, an Empirical Bayes approach to FDR analysis that incorporates effect size estimates and standard errors, and assumes the distribution of the actual effects is unimodal, with a mode at 0.[40] We also generated a random subset of the data (50% of expressed genes), computed a list of canonical covariance matrices (*cov_canonical* function), and used these data and matrices to estimate the correlation structure in the null tests (*estimate_null_correlation* function). We then set up the main data objects (i.e., "strong" and "random") with this correlation structure in place (*mash_set_data* function). We used the strong tests to set up data-driven covariances by performing PCA on the data (using 5 PCs; *cov_pca* function) and using the resulting 5 candidate covariance matrices to

initialize and perform "extreme deconvolution" (*cov_ed* function).[111] We then estimated canonical covariances from the random tests and then fit mash to the random tests using both data-driven and canonical covariances. We extracted the fitted g mixture from this model and specified this mixture model when fitting mash to the strong tests. Significant genes (i.e., 'sex-biased genes') passed an LFSR cutoff of 0.05.

Human (GTEx) comparison

We estimated sex effects across 10 tissues from the human GTEx data (V8), including the amygdala, BA24, caudate nucleus, cerebellar hemisphere, BA9, hippocampus, hypothalamus, nucleus accumbens, putamen, and substantia nigra (mean n = 39F/119M; Table S26). Technical replicates for two regions ("Cortex" and "Cerebellum") were excluded. Using the EMMA models described above, we modeled gene expression (within each region and for each gene) as a function of sex, age, RIN, experimental batch, and ischemic time. We then applied MASH to the model outputs (as described above). To test for the consistency of sex effects on gene expression across data sets, we compared the results across 8 overlapping regions (AMY/amygdala, ACCg/BA24, CN/caudate, dmPFC/BA9, DG/hippocampus, CA3/hippocampus, VMH/hypothalamus, Pu/putamen) for all one-to-one orthologues. We report Spearman's rank order correlation coefficients and the quadrant count ratio (q = (# concordant - # discordant)/total). We do not expect that differences in mapping methods impact this cross-species comparison, as previous work showed that remapping the GTEx data using *kallisto* produced similar expression levels (for non-Y chromosome genes).[112]

Conservation analysis

Following Naqvi and colleagues,[22] we modeled adjusted normalized expression levels for each one-to-one orthologue (within each tissue) as a function of sex and species using linear mixed models (LMMs). Prior to modeling, we merged the human and macaque count matrices, calculated normalization factors (*calcNormFactors* in the R package *edgeR*),[88] and used the *voomWithQualityWeights* function (R package *limma*)[89] to normalize expression levels and estimate species-specific variances (for all genes with mean CPM >10). We then adjusted the normalized expression values (within each species) using the *removeBatchEffect* function (R package *limma*) (human covariates: age, ischemic time, RIN, and experimental batch; macaque covariates: age, rank, RIN, and library batch). We modeled adjusted expression levels as a function of sex (fixed effect) and species (random effect) to control for between-species differences in sample size and variability. The latter was done using the *duplicateCorrelation* function (R package *limma*), with species specified as the block variable. Conserved genes were identified within each overlapping brain region as those with significant sex terms in the LMMs ($p_{adj} < 0.05$) and βs estimated in the consistent direction in both species-specific mashr analyses. Differences between the conserved sex-biased gene set (from the LMM analysis) and the species-specific sex-biased gene sets (from the EMMREML/mashr analyses) reflect methodological differences between these approaches, including: i) removing age and technical effects prior to LMM modeling vs. simultaneous modeling in EMMREML; ii) gains in power from combining the species in the LMM analysis; iii) exploiting shared patterns across regions in mashr vs. LMM modeling within regions; and iv) applying voom normalization with (LMM) or without (EMMREML/mashr) quality weights.

Cell type enrichment analysis

We tested for cell type enrichment among male- and female-biased genes using cell type markers from the R package *BRETIGEA* (BRain cEll Type specIfic Gene Expression Analysis).[44] In this package, the 'markers_df_brain' data frame contains the top 1000 marker genes (ranked by specificity) from each of the six major brain cell types (i.e., astrocytes, endothelial cells, microglia, neurons, oligodendrocytes, and OPCs), which were estimated from their meta-analysis of brain cell gene expression data from both humans (*Homo sapiens*) and mice (*Mus musculus*). *Homo sapiens* gene names were converted to *Macaca mulatta* Ensembl gene IDs using the *bioMart* R package. Sex-biased gene sets included any genes that were significantly male-biased or female-biased in any region (LFSR < 0.05). Fisher's exact tests were used to test for cell type-specific enrichments (*fisher.test* function in the R package *stats*; alternative = 'greater'). p values were adjusted using the Benjamini-Hochberg method, and tests with adjusted p values less than 0.05 were considered significant. This analysis was also performed on sex-biased genes (LFSR < 0.05 in any region) identified in our analysis of the human GTEx data (described above).

Deconvoluting cell type proportions and modeling sex effects on gene expression

Given that sex-biased gene sets were enriched for certain cell types (see results), we also estimated sex effects after performing cell type deconvolution analysis in the R package *BRETIGEA*.[44] Using the cell type marker genes described above, cell type deconvolution analyses were conducted within each region. First, the effects of library batch and RIN were removed from each normalized, filtered expression matrix using the *removeBatchEffect* function (R package *limma*). This matrix and the marker gene list were used as inputs to estimate the relative cell type proportions (i.e., surrogate proportion variables (SPVs) for each cell type). This was performed by the *findCells* function, using the top 50 markers for each cell type and the singular value decomposition (SVD) dimension reduction approach, and scaling the gene expression data from each marker gene prior to using it as an input for dimension reduction. SPVs are eigenvectors of an SVD and do not directly quantify cell type proportions; rather, SPVs reflect relative differences in cell type composition within each cell type and, therefore, some SPVs will take on negative values. Sex differences in SPVs were tested using t-tests

(*t.test* function in the R package *stats*). Finally, we adjusted each row of gene expression for sample differences in relative cell type proportions using the *adjustCells* function, which outputs the residuals from a linear model for downstream analysis.

We also validated the SPVs estimated from BRETIGEA using a complementary cell type deconvolution approach – CIBERSORT.[113] Specifically, we used macaque brain single nuclei RNAseq data from our previous work[114] as a reference. For both human and macaque bulk expression datasets, we used the TPM matrices (normalized to everything measured). We then subset the human data to genes with single copy orthologs (SCOs). Additional genes were removed if SCOs included multiple genes with the same gene name. We then reindexed the human nonredundant SCOs with their corresponding macaque ENSEMBL IDs. For the reference dataset, we calculated CPMs (normalized to all genes measured) and removed genes that did not appear in either the macaque or human bulk datasets. We then: i) dropped all cells with UMI < 500 and those from cerebellar, midbrain, or brainstem regions; ii) dropped all rare cell labels by filtering out cell classes with < 750 cells remaining; and iii) randomly sampled 100 cells per cell class to use as the reference. Sex differences in cell type proportions were tested using t-tests (*t.test* function in the R package *stats*). We obtained expected cell type proportions (Figure S18) and found that estimated cell type proportions per sample were highly correlated across CIBERSORT and BRETIGEA (except for astrocytes; Figure S18). These findings are consistent with multiple previous studies that applied both BRETIGEA and CIBERSORT.[115,116] Differences in estimated cell type proportions across methods (in this study) are likely to reflect that: i) BRETIGEA incorporates cell type markers derived from human and mouse data while our CIBERSORT analysis used a macaque single nuclei gene expression reference; and ii) we removed age and technical effects prior to modeling in BRETIGEA but not in CIBERSORT.

For each gene in the adjusted expression matrix (from BRETIGEA analysis above), we estimated the effect of sex on expression using Equation 2 below (see ‘modeling sex effects on gene expression’ section for details). Technical effects (i.e., library batch and RIN) were not included here since they were removed prior to the estimation of relative cell type proportions.

Y = intercept + sex + age + ordinal rank

$$y = \boldsymbol{\mu} + \boldsymbol{\nu}\boldsymbol{\beta} + a\gamma + r\delta + r^2\delta^2 + \boldsymbol{\varepsilon}, u \sim MVN\left(0, \boldsymbol{\sigma}_u^2 K\right), \boldsymbol{\varepsilon} \sim MVN\left(0, \boldsymbol{\sigma}_e^2 I\right) \quad \text{(Equation 2)}$$

We then used the outputs from these models (i.e., per gene βs and their standard errors within each of 15 regions) as inputs for multivariate adaptive shrinkage models (see ‘multivariate adaptive shrinkage (MASH)’ section above for details). Significant genes (i.e., ‘sex-biased genes’) passed an LFSR cutoff of 0.05.

Gene ontology enrichment analyses

Gene ontology (GO) enrichment analyses were performed using the R packages *topGO*[117] and *ViSEAGO*.[118] GO term names were obtained from Ensembl using the *Ensembl2GO* and annotate functions. Enrichment analyses were conducted on: 1) macaque brain male-biased genes and female-biased genes, with each set of genes defined as those that were significantly biased in mashr (LFSR < 0.05) in any region (excluding Y chromosome genes, which are not expressed in females); and ii) genes that are female-biased in macaques and/or humans and also upregulated in ASD. For these tests test, background genes represented all genes that were: i) expressed in the macaque brain but not in the macaque male- or female-biased gene set of interest; or ii) expressed one-to-one orthologs that were not included in the target gene set. We used Fisher’s exact tests, which are based on gene counts. Enrichments with nominal $p < 0.05$ were considered significant, as suggested by the *topGO* package’s creators. The parent child algorithm[119] was used since it determines overrepresentation of terms in the context of annotations to the term’s parents. Other approaches to measuring overrepresentation of GO terms cannot cope with the dependencies resulting from the structure of GO because they analyze each term in isolation. The parent child approach reduces the dependencies between the individual term’s measurements, and thereby avoids producing false-positive results owing to the inheritance problem. We computed the semantic similarity between GO terms using Wang’s method (compute_SS_distances function),[120] and clustered GO terms using Ward’s clustering criterion (GOterms_heatmap function).[121]

Disease gene set enrichment analysis

Enrichment tests for disease ontology (DO) terms were performed using human risk genes downloaded from the DISEASES resource, which integrates the results of text mining and manually curated disease-gene associations, cancer mutation data, and genome-wide association studies from existing databases (dataset = text mining channel, filtered).[122] *Macaca mulatta* Ensembl IDs were linked to human diseases from this database using one-to-one human orthologues (and their associated proteins) from the R package *bioMart*. Diseases with at least 10 associated genes were retained for further analysis (n = 1257 for macaques, n = 1208 for humans).

For each disease, we performed three complementary enrichment tests: 1) Fisher’s exact tests were performed on 2x2 contingency tables, with genes separated into those with positive or negative mean *mashr* βs (*fisher.test* function in the R package *stats*; alternative = ‘greater’); 2) two-sample Kolmogorov-Smirnov (K-S) tests on a list of genes ranked according to their mean standardized *mashr* β (using two alternative hypotheses, i.e., the cumulative distribution function for the target set is either less than or greater than that of the background set, and the null is that the distribution functions are the same) (*ks.test* function in the R package *stats*; alternative = ‘less’ or ‘greater’); and 3) the fast GSEA method implemented by *fgsea*[123] on a list of genes ranked according to their mean standardized *mashr* β, where the null distribution is derived from resampling random gene sets (and estimating their enrichment

scores). These tests provide slightly different insights into the data, since approach #1 tests for an association between each disease gene set and sex-biased expression, regardless of the relative order or magnitude of per-gene sex effects, while #2 incorporates the relative order of per-gene sex effects and #3 considers both the relative order and magnitude of per-gene sex effects. *p* values were adjusted using the Benjamini-Hochberg method, and tests with adjusted *p* values less than 0.05 were considered significant. These analyses were also run on the mean standardized β from our analysis of the human GTEx data (described above).

We also tested whether the sex-biased genes identified here tend to exhibit altered expression levels in human disease. ASD-upregulated and -downregulated genes were collected from Gandal et al.[59] and Voineagu et al.[60] Genes in ASD-upregulated and -downregulated co-expression modules were collected from Voineagu et al.[60] and Gupta et al.[61] *Macaca mulatta* Ensembl IDs were linked to human diseases from this database using one-to-one human orthologues (and their associated proteins) from the R package *bioMart*. For each ASD gene set, Fisher's exact tests were performed on female- and male-biased gene sets (for macaques and humans, separately).

Functional enrichment analyses of conserved/divergent genes

We used g:Profiler[124] to investigate the functions and human phenotypes associated with genes showing a conserved or divergent pattern of sex-bias in human and macaque brains. Analyses were run on three target sets: i) $n = 3{,}922$ conserved male-biased genes; ii) $n = 1{,}105$ conserved female-biased genes; and iii) $n = 4{,}681$ genes that do not show a conserved pattern. These target lists exclude $n = 16$ genes that showed inconsistent patterns of conserved sex-bias across regions (i.e., those with conserved female-bias in some regions and conserved male-bias in others). All $n = 9{,}724$ expressed one-to-one orthologues were included as a custom background. Significant terms were identified as those with $p_{adj} < 0.05$. *p* values were adjusted using the default g:SCS algorithm in g:Profiler. Results from all functional information sources are provided (GO:MF = gene ontology molecular function; GO:CC = gene ontology cellular compartment; GO:BP = gene ontology biological function; KEGG. = KEGG Reactome database; HP = human phenotype ontology. Only significant terms (g:SCS < 0.05) were visualized.

Motif enrichment analyses

We used HOMER (Hypergeometric Optimization of Motif EnRichment)[102] to analyze the promoters of genes and look for motifs that are enriched in the target gene promoters relative to other promoters. The target gene sets consisted of all genes that were sex-biased, male-biased, or female-biased (LFSR < 0.05) in at least one region. We searched for motifs from -1000 to $+300$ relative to the transcriptional start site (TSS) using HOMER's curated set of 414 known vertebrate motifs. The program assigns weights to the background promoters based on the distribution of GC content in the target gene promoters to ensure that comparable numbers of low and high-GC promoters are analyzed. It also performs auto-normalization to remove sequence content bias from lower order oligos (1/2/3-mers) by adjusting background weights based on the target distribution. The hypergeometric distribution is used to score motifs. Enrichment *p* values less than 0.05 were considered significant.

Sex prediction

For each region, we created sex prediction models using residual gene expression values (i.e., expression levels after removing the effects of age, ordinal rank, RIN, library batch, and relatedness using the EMMA models and the normalized, filtered expression matrix described above). Specifically, we implemented gradient boosted models (GBM) using leave-one-out cross validation in the R package *caret*. We fit these models across various tuning parameters (interaction depths = 1, 3, 5, 9; number of trees = 50, 100, 150, 200, 250; shrinkage = 0.1; n.minobsinnode = 5), and models with the highest receiver operating characteristic (ROC) values were selected as the optimal model for each region. For each optimal model, we extracted the prediction probabilities for each sample, calculated the relative influence of each gene using the GBM model based technique in the *varImp* function (i.e., relative influence = the reduction in sums of squared error due to any split on that predictor, summed over all trees in the model[125] scaled to a maximum value of 100) and calculated multiple performance metrics, including accuracy, sensitivity, and specificity (see below). This was done separately for: 1) combined X chromosome and autosomal genes; 2) autosomal genes only; and 3) X chromosome genes only (results are in Tables S21–S23). While most genes (87%) were only influential in one region (Table S22), they tended to be ubiquitously expressed (89% expressed in at least 13 regions), which is likely to reflect that the magnitude of sex differences in expression per gene varies across brain regions, even for shared sex-biased genes. Female samples that were misclassified in X chromosome gene models tend to exhibit relatively low expression of the most influential X chromosome genes in those regions (Figure S19), which may partially reflect variability in XCI escape across female individuals and tissues.[126]

True "Positives" (TP) = the number of samples correctly identified as female.
True "Negatives" (TN) = the number of samples correctly identified as male.
False "Positives" (FP) = the number of samples incorrectly identified as female.
False "Negatives" (FN) = the number of samples incorrectly identified as male.
Accuracy = (TP + TN)/(TP + TN + FP + FN)
Sensitivity = TP/(TP + FN)
Specificity = TN/(TN + FP)

We tested for age effects on the accuracy of our sex prediction models by modeling mean prediction probability of known sex per individual as a function of age. We also examined age effects on the variability of our sex predictions by modeling the standard

deviation of known sex prediction probabilities per individual as a function of age. To test for the stability of these effects within old and young individuals and for varying sample sizes, we examined age effects on accuracy within subsamples of young ($\leq$ 8 years) and old (> 8 years) individuals. Specifically, for each region, we randomly sampled 7 males and 7 females (6 for the LGN) and used these individuals to re-create sex prediction models (using both X chromosome and autosomal genes) and estimate known sex prediction probabilities per individual.

Sex differences in gene expression heterogeneity

To investigate sex and age differences in gene expression heterogeneity, we calculated Euclidean distances for residual gene expression between all pairs of samples using the *euc* function in the R package *bioDist*,[127] maintained all pairs of same-sex, same-age group (< or > 8 years), and cross-individual samples, and compared average distances between sex and age group combinations using Tukey's HSD.

Interactive sex and aging effects

To investigate the potential interactive effects of sex and aging on macaque brain transcriptomes and how these effects may impact our sex prediction models, we: 1) pulled results from our previous analysis of age-related changes in macaque brain transcriptomes[35] (age-related increase = $\beta > 0$, LFSR < 0.2; age-related decrease = $\beta > 0$, LFSR < 0.2); and 2) re-ran those analyses (using the same model structure) within males and females only. Results are described for the top 5 most influential genes in autosomal sex prediction models for $n = 8$ regions with $\geq$1 misclassified sample.

Chromosome overrepresentation analysis

We tested for chromosome overrepresentation among sex-biased genes (LFSR < 0.05 in any region) using one-sided Fisher's Exact tests (*fisher.test* function in the R package *stats*; alternative = 'greater'). We also ran this analysis on male- and female-biased gene sets separately. p values for each chromosome were adjusted using the Bonferroni correction (*p.adjust* function in the R package *stats*) and adjusted p values less than 0.05 were considered significant.

Tissue specificity

For each gene in the macaque dataset, we calculated τ (a measure of tissue specificity) across all brain tissues sampled using the following formula[70,128]:

$$\tau = \frac{\Sigma_i \left[1 - \frac{ln(TPM_i)}{ln(TPM_{max})} \right]}{N - 1}$$

where N is the number of tissues examined, TPM_i is the mean TPM per gene within each region, and TPM_{max} is the highest expression level detected for a given gene over all tissues examined (i.e., maximum TPM_i value). The value of τ ranges from 0 to 1, with lower values indicating an expression pattern that is evenly distributed through all tissues examined and higher τ values indicating more variation in expressional levels across tissues and, thus, a greater degree of tissue specificity. Following other studies,[70] we did not normalize the τ calculations in order to reflect this biological reality of gene expression levels. For genes with expression values approaching 0 and low TPM_{max}, calculations of τ are subject to sampling stochasticity. In order to reduce this effect, TPM_i was set to 1 for samples with no detected expression (i.e., less than 1 TPM).[70] Per gene, we modeled log(τ) as a function of the absolute value of the difference between male mean and female mean residual expression values (averaged across all samples). To test for a significant relationship between these variables, we: 1) calculated the Spearman rank order correlation; and 2) calculated the moving average log(τ) across non-overlapping window increments of 0.05 expression-level differences, and then calculated the best-fit linear regression line for this moving average. We ran these analyses on all non-Y chromosome genes (as their expression is limited to males).

Comparisons of loss-of-function (LOF) mutation tolerance

We used LoFtools[129] to assign LOF metrics to each gene. This database is based on the ratio of loss-of-function to synonymous mutations, with lower LoFtool percentiles representing more intolerance to functional variation. *Homo sapiens* gene names from the LoFtools list were converted to *Macaca mulatta* Ensembl gene IDs using the *bioMart* R package, resulting in a set of 7786 orthologous genes. Per gene, we modeled LOF intolerance percentiles as a function of the absolute value of the difference between male and female mean residual expression values (see above) across all samples and regions. We tested for a significant association between these variables as above (see tissue specificity).

Comparisons of genetic variance

For each gene within each region, the genetic variance of expression (*Vu*) was estimated from the EMMA models described above. Per gene and within each region, we modeled log(*Vu*) as a function of the absolute value of the difference between male and female mean residual expression values (see above). The structure of this data resulted in a bimodal distribution for estimated values of *Vu*, so we evaluated the relationship between *Vu* and sex differences in expression separately within each distribution. This bimodality

reflects estimates of Vu that are $\sim$0 versus those that are >0. This is reflected by observations that the pedigree does not improve the likelihood of EMMREML models for genes in the lower Vu distribution (i.e., $Vu \sim 0$) (mean likelihood improvement = $-1.44e-9$), whereas the pedigree improves the likelihood of EMMREML models for genes in the upper Vu distribution (i.e., $Vu > 0$) (mean likelihood improvement = 0.49) (Figure S15). We tested for a significant association between these variables as above (see tissue specificity).

Cell Genomics, Volume *4*

Supplemental information

Evolutionary and biomedical implications of sex differences in the primate brain transcriptome

Alex R. DeCasien, Kenneth L. Chiou, Camille Testard, Arianne Mercer, Josué E. Negrón-Del Valle, Samuel E. Bauman Surratt, Olga González, Michala K. Stock, Angelina V. Ruiz-Lambides, Melween I. Martínez, Cayo Biobank Research Unit, Susan C. Antón, Christopher S. Walker, Jérôme Sallet, Melissa A. Wilson, Lauren J.N. Brent, Michael J. Montague, Chet C. Sherwood, Michael L. Platt, James P. Higham, and Noah Snyder-Mackler

Supplementary Figures

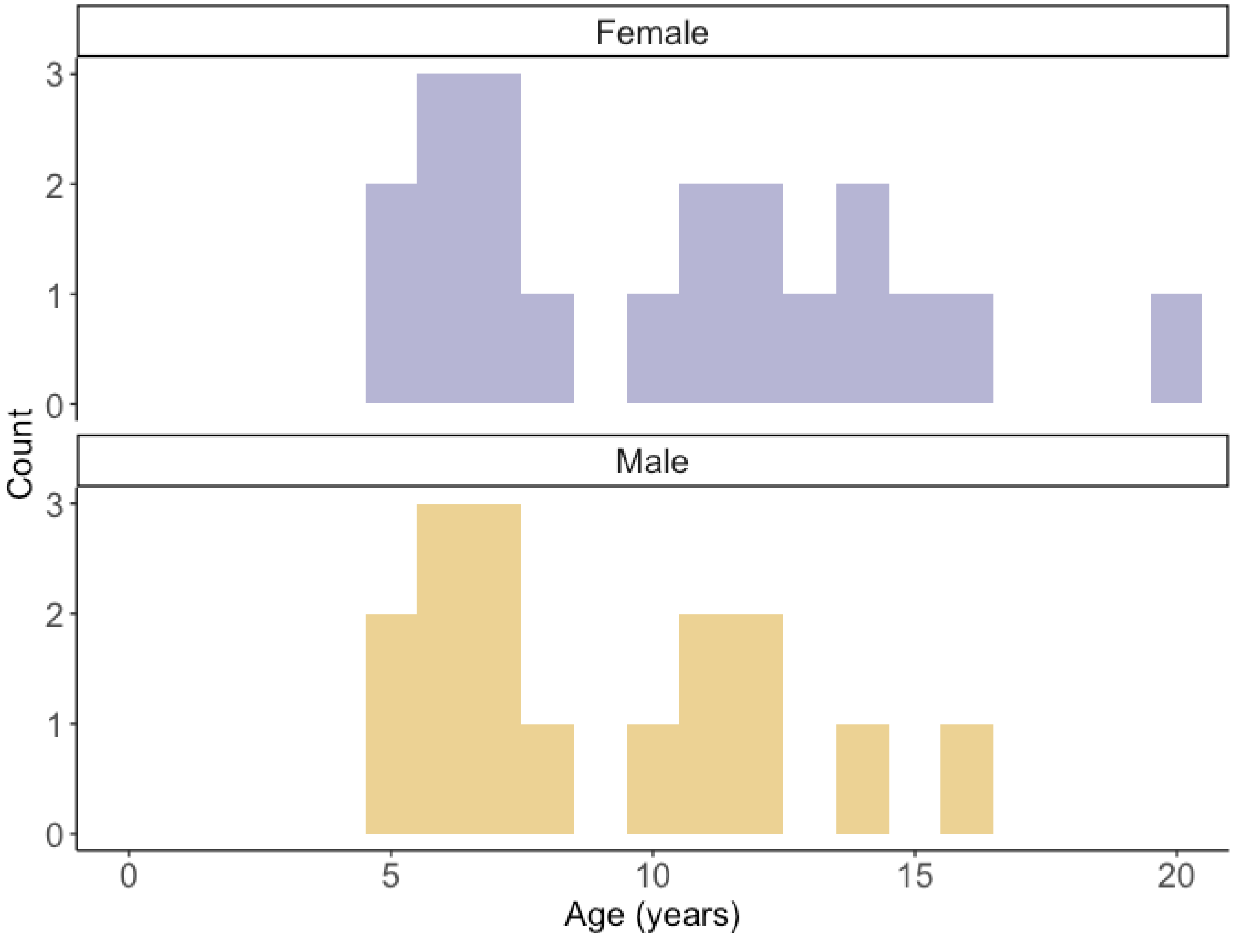

Figure S1 | Age distribution of adult male and female macaques included in this study (see Methods)

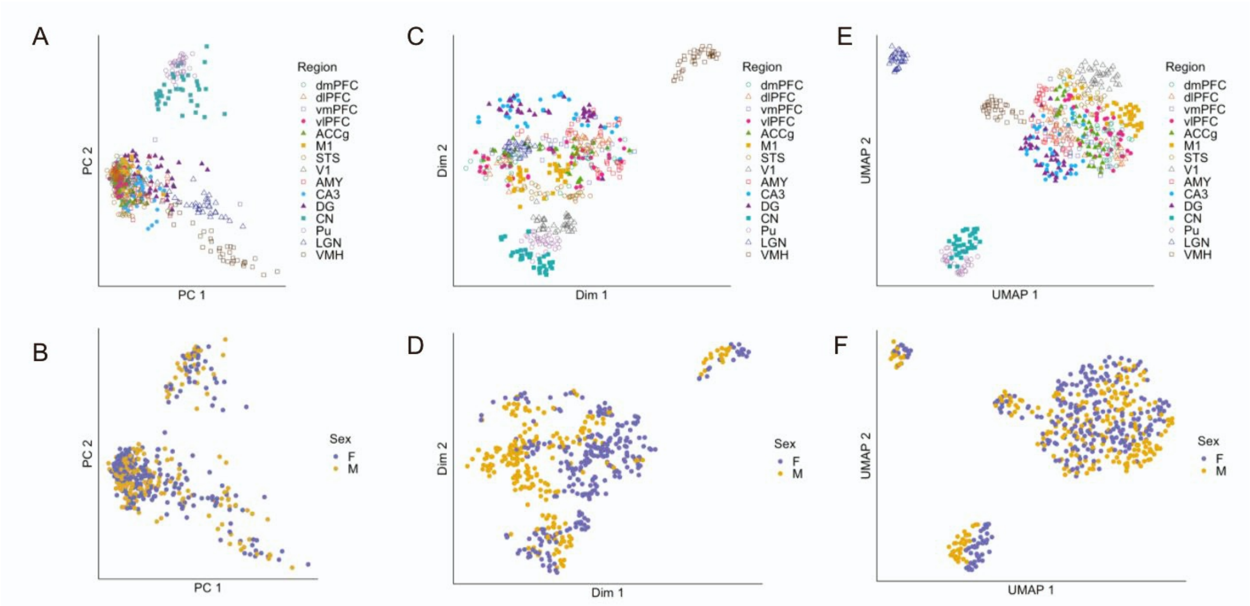

Figure S2 | Visualization of expression data obtained for study, related to Figure 1B.

A) Principal components analysis (PCA) plot of expression data. Each point represents one sample (N=527). Colors and shapes indicate region (see legend).

B) Principal components analysis (PCA) plot of expression data. Each point represents one sample (N=527). Colors indicate sex (see legend).

C) t-SNE plot of expression data. Each point represents one sample (N=527). Colors and shapes indicate region (see legend).

D) t-SNE plot of expression data. Each point represents one sample (N=527). Colors indicate sex (see legend).

E) Uniform Manifold Approximation and Projection (UMAP) plot of expression data. Each point represents one sample (N=527). Colors and shapes indicate region (see legend).

F) Uniform Manifold Approximation and Projection (UMAP) plot of expression data. Each point represents one sample (N=527). Colors indicate sex (see legend).

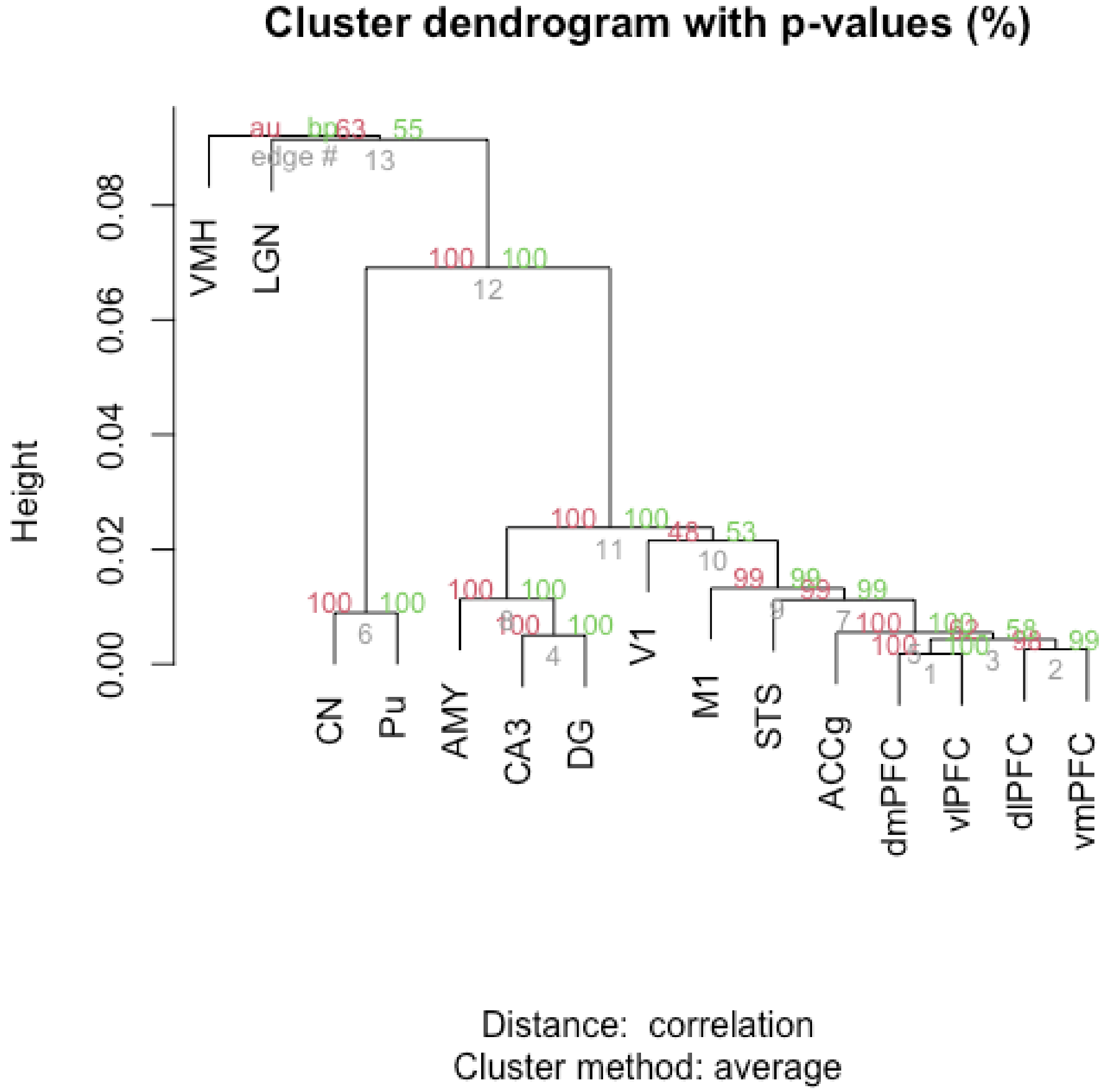

Figure S3 | Hierarchical clustering of brain regions, related to Figure 1B.

Dendrogram depicting hierarchical clustering of brain regions (using expression values averaged across samples within each region after removing batch effects). Approximately unbiased (AU) p-values (red) and bootstrap probability (BP) values (green) are depicted for each cluster. AU values are calculated using multiscale bootstrap resampling, while BP values are calculated by the ordinary bootstrap resampling.

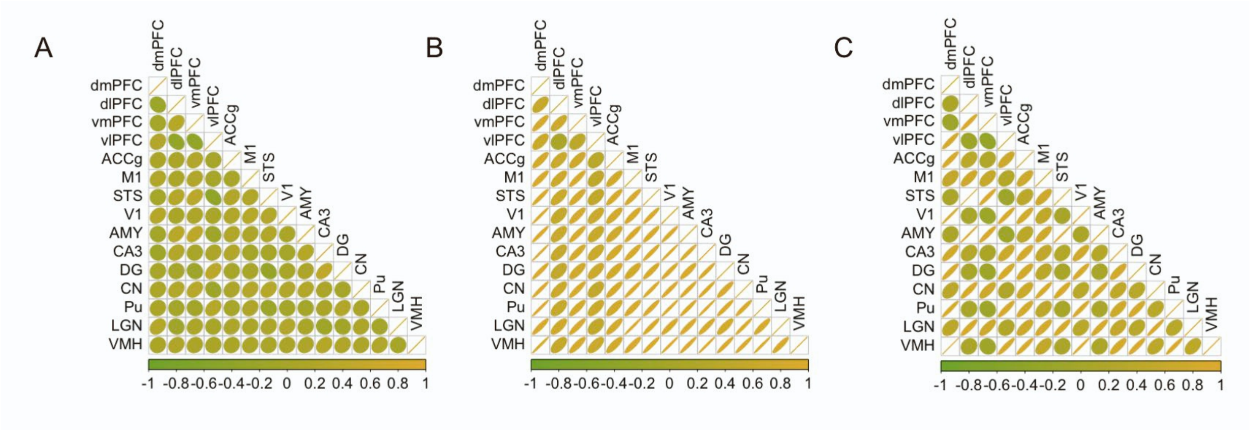

Figure S4 | Correlations of sex effects across regions, related to Figure 2A.

A) Correlations are shown for un-shrunken sex effect sizes (from EMMREML) using cell type corrected data across regions. Of these inter-regional correlations, 83 are significantly positive ($p<0.05$), 15 are significantly negative ($p<0.05$), and are 7 not significant ($p>0.05$). Yellow = positive correlation, green = negative correlation, circle = weak correlation; oval = stronger correlation in the given direction.

B) Correlations are shown for shrunken sex effect sizes (from MASHR) across regions. Of these inter-regional correlations, 105 are significantly positive ($p<0.05$). Yellow = positive correlation, green = negative correlation, circle = weak correlation; oval = stronger correlation in the given direction.

C) Correlations are shown for shrunken sex effect sizes (from MASHR) using cell type corrected data across regions. Of these inter-regional correlations, 96 are significantly positive ($p<0.05$) and 9 are significantly negative ($p<0.05$). Yellow = positive correlation, green = negative correlation, circle = weak correlation; oval = stronger correlation in the given direction.

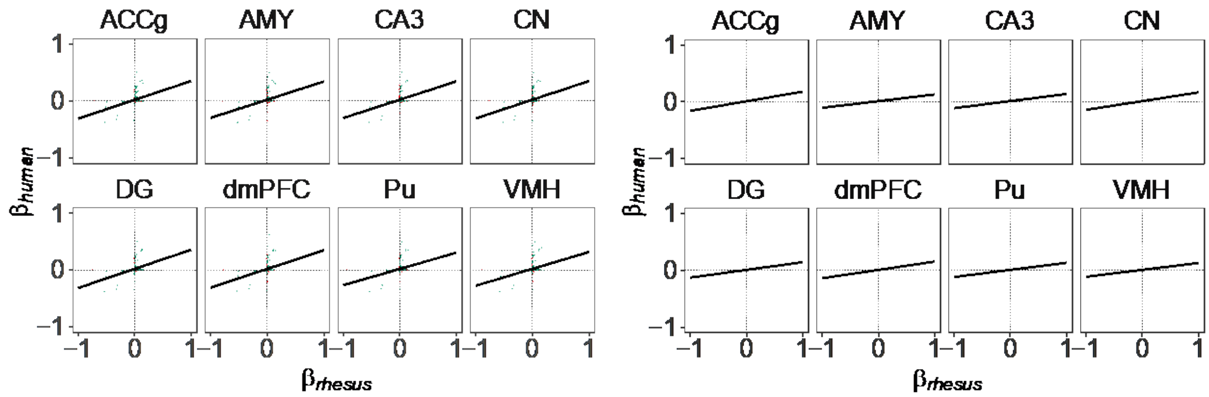

Figure S5 | Comparisons of sex effects in macaques vs. humans, related to Figure 3A.

Scatterplots of estimated sex effects for all one-to-one orthologous X chromosome genes (left) or autosomal genes (right). Green points represent genes with concordant sex-bias across species, while red points represent discordance.

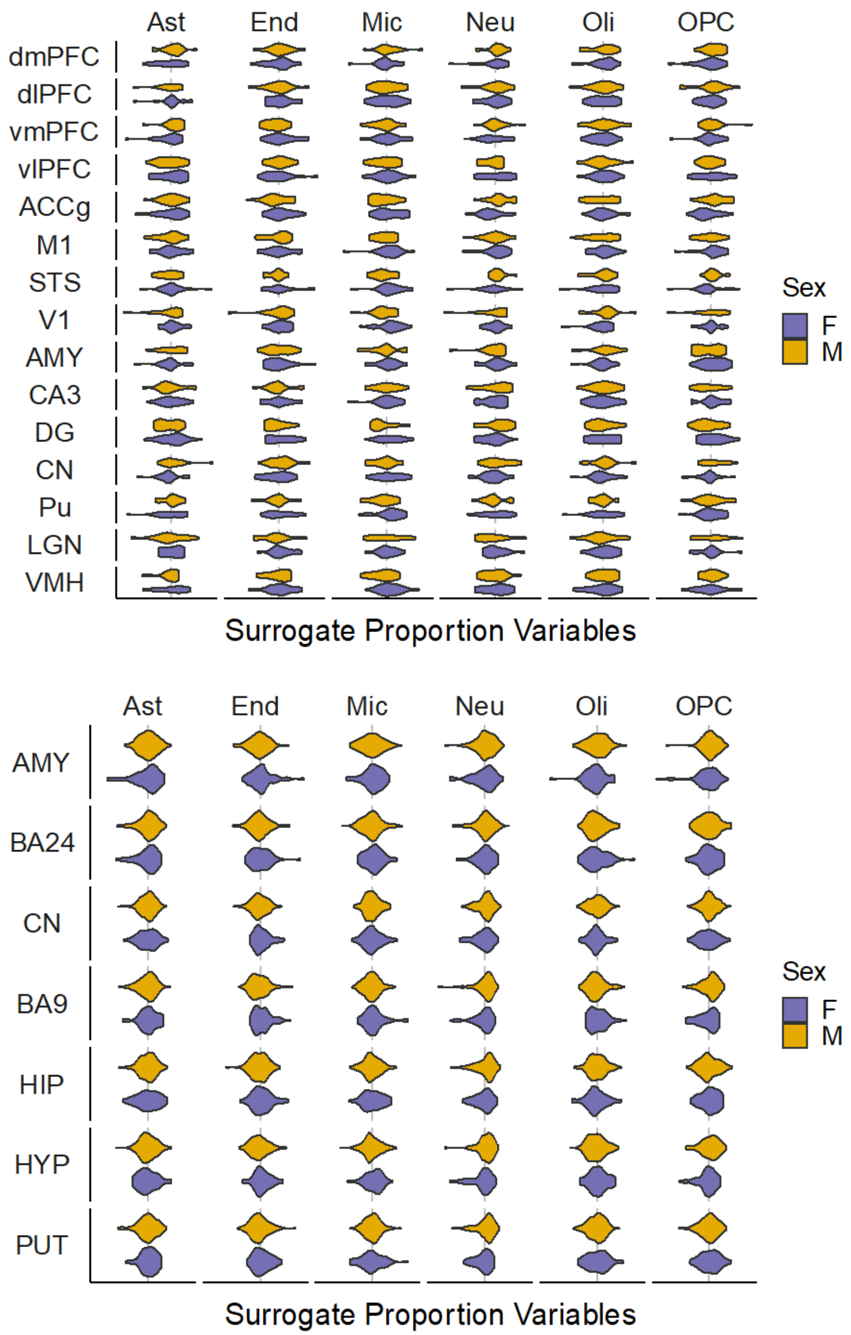

Figure S6 | Sex differences in estimated cell type proportions, related to Figure 3D.

Violin plots of estimated surrogate proportion variables (SPVs) estimated using BRETIGEA (see Methods) for macaques (top) and humans (bottom) within each region (y-axis). Vertical dashed grey line indicates SPV = 0. Ast = astrocytes; End = endothelial; Mic = microglia; Neu = neurons, Oli = oligodendrocytes; OPC = oligodendrocyte precursor cells.

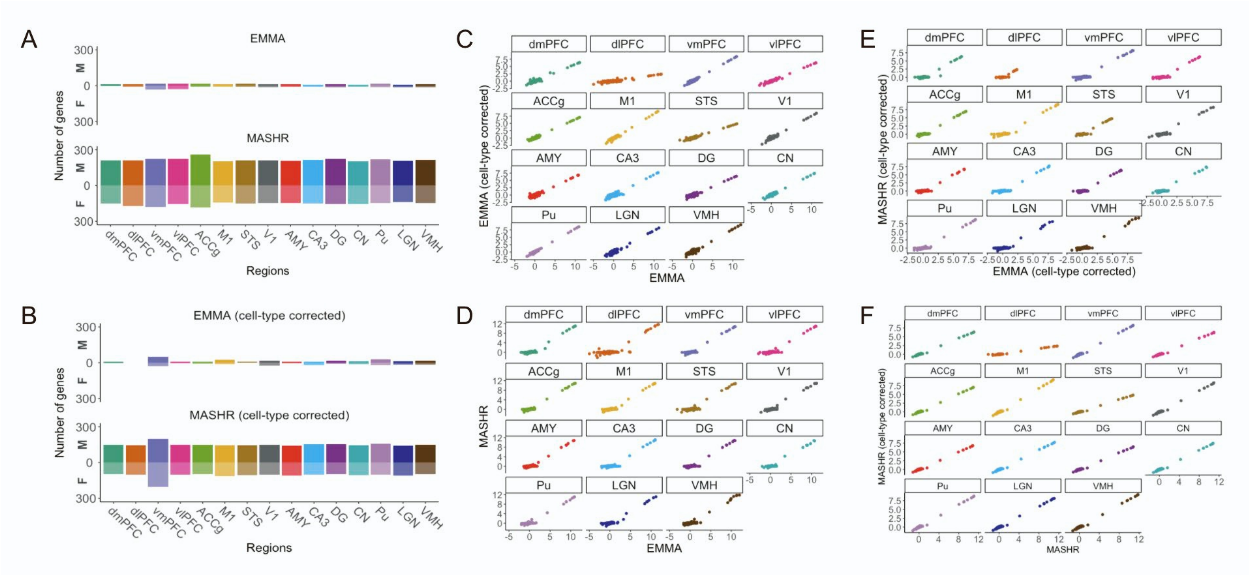

Figure S7 | Comparisons of unadjusted vs. cell type-adjusted analyses, related to Figures 4A-C.

A) Counts of sex-biased genes identified using EMMREML (top) and mashr (bottom) on unadjusted expression data. M = male-biased, F = female-biased.

B) Counts of sex-biased genes identified using EMMREML (top) and mashr (bottom) on cell-type adjusted expression data. M = male-biased, F = female-biased.

C) Sex effects (β) estimated for each gene within each region (x-axis: EMMREML, unadjusted expression data; y-axis: EMMREML, cell-type adjusted expression data) (positive values = male-biased; negative values = female-biased)

D) Sex effects (β) estimated for each gene within each region (x-axis: mashr, unadjusted expression data; y-axis: EMMREML, unadjusted expression data) (positive values = male-biased; negative values = female-biased)

E) Sex effects (β) estimated for each gene within each region (x-axis: EMMREML, cell-type adjusted expression data; y-axis: mashr, cell-type adjusted expression data) (positive values = male-biased; negative values = female-biased)

F) Sex effects (β) estimated for each gene within each region (x-axis: mashr, unadjusted expression data; y-axis: mashr, cell-type adjusted expression data) (positive values = male-biased; negative values = female-biased). Estimated sex effects tended to be in the same direction (i.e., male- or female-biased) whether cell type proportions were considered (74% concordance across all estimated effects; 99% concordance across effects that were significant in at least one analysis; $\rho = 0.635$, $p < 2.20e-16$).

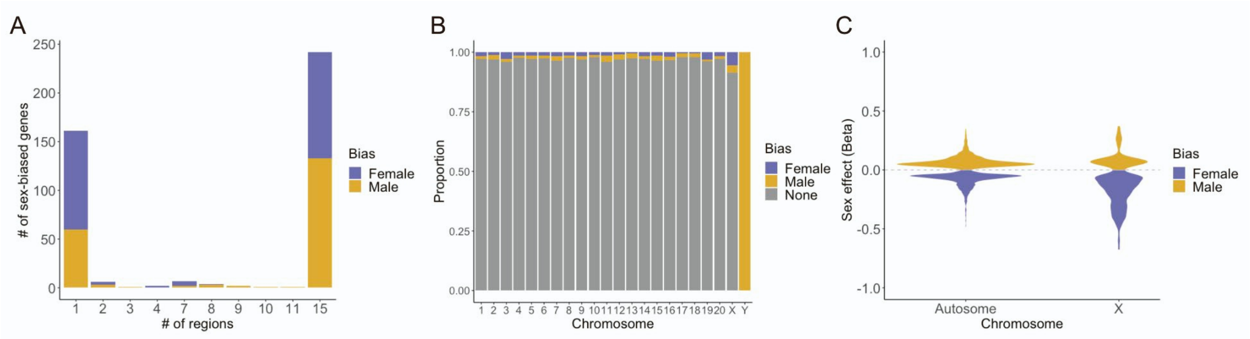

Figure S8 | Results for cell-type adjusted mashr analyses, related to Figures 4A-C.

A) Stacked bar chart of the number of sex-biased genes shared across different numbers of regions. 3.3% (422/12672) of brain expressed genes were differentially expressed in at least one region, and most of these genes exhibited sex-bias in a majority of regions (58.8% were biased in at least 8 tissues).

B) Proportions of genes on each chromosome that are not biased in any region (grey), female-biased in at least 1 region (purple), or male-biased in at least 1 region (yellow). Of the identified sex-biased genes, 9% were X-linked, 2% were Y-linked, and 89% were autosomal.

C) Violin plots of sex effect sizes for sex-biased autosomal versus X chromosome genes. Female-biased genes exhibited significantly larger (t-test: $p < 0.001$) magnitudes of their sex effects (mean $|\beta| = 0.10$) than male-biased genes (mean $|\beta| = 0.07$; excluding N = 9 Y chromosome genes, which are not expressed in females). In particular, female-biased X-linked genes exhibited significantly larger sex effects (N=25 genes; mean $|\beta| = 0.20$) than either male-biased X-linked genes (N = 14; mean $|\beta| = 0.10$; Tukey's HSD $p_{adj} < 0.001$) or sex-biased autosomal genes (female: N = 196, mean $|\beta| = 0.08$, $p_{adj} < 0.001$; male: N = 183: mean $|\beta| = 0.07$, $p_{adj} < 0.001$).

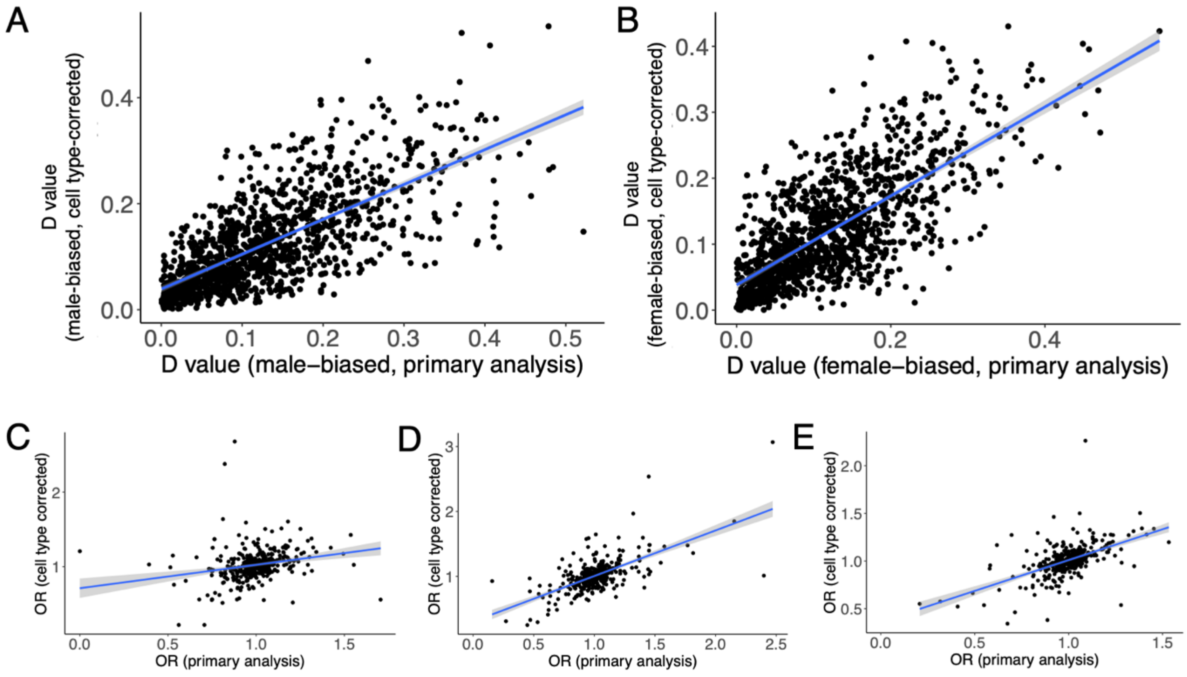

Figure S9 | Comparisons of macaque vs. human disease and motif enrichments, related to Figures 4D and 5.

A) D values for N=1257 diseases across primary and cell-type-corrected analyses (direction = male-biased; ρ = 0.722; p < 2.2e-16)

B) D values for N=1257 diseases across primary and cell-type-corrected analyses (direction = female-biased; ρ = 0.704; p < 2.2e-16)

C) ORs for N=414 motifs across primary and cell type-corrected analyses for male-biased genes (ρ = 0.278; p = 3.048e-15)

D) ORs for N=414 motifs across primary and cell type-corrected analyses for female-biased genes (ρ = 0.542; p < 2.2e-16)

E) ORs for N=414 motifs across primary and cell type-corrected analyses for sex-biased genes (ρ = 0.552; p < 2.2e-16)

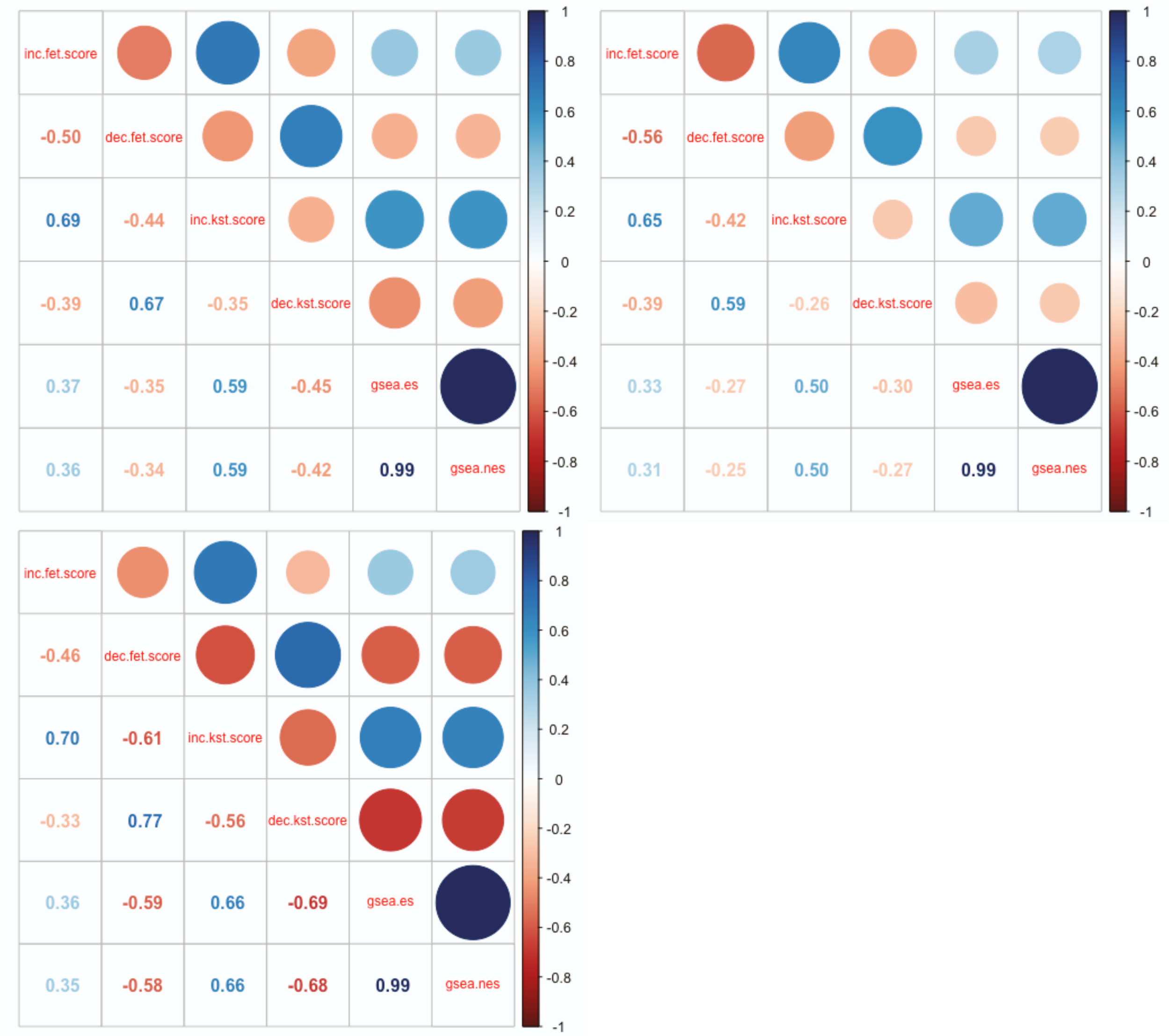

Figure S10 | Similarities across disease enrichment methods, related to Figure 5 (see Methods).

Correlations between disease enrichment scores across three approaches for macaque data (top left), macaque cell type-corrected data (top right), and human data (bottom). “inc.fet.score” = odds ratios (male-biased genes); “dec.fet.score” = odds ratios (female-biased genes); “inc.kst.score” = D-values (male-biased genes); “dec.kst.score” = D-values (female-biased genes); “gsea.es” = GSEA enrichment score (positive = male-biased, negative = female-biased); “gsea.nes” = GSEA normalized enrichment score (positive = male-biased, negative = female-biased). For the purposes of the correlation plot for the human data, diseases without enrichment scores (gsea.es = NA) were removed, and ORs = Inf were set to 15.

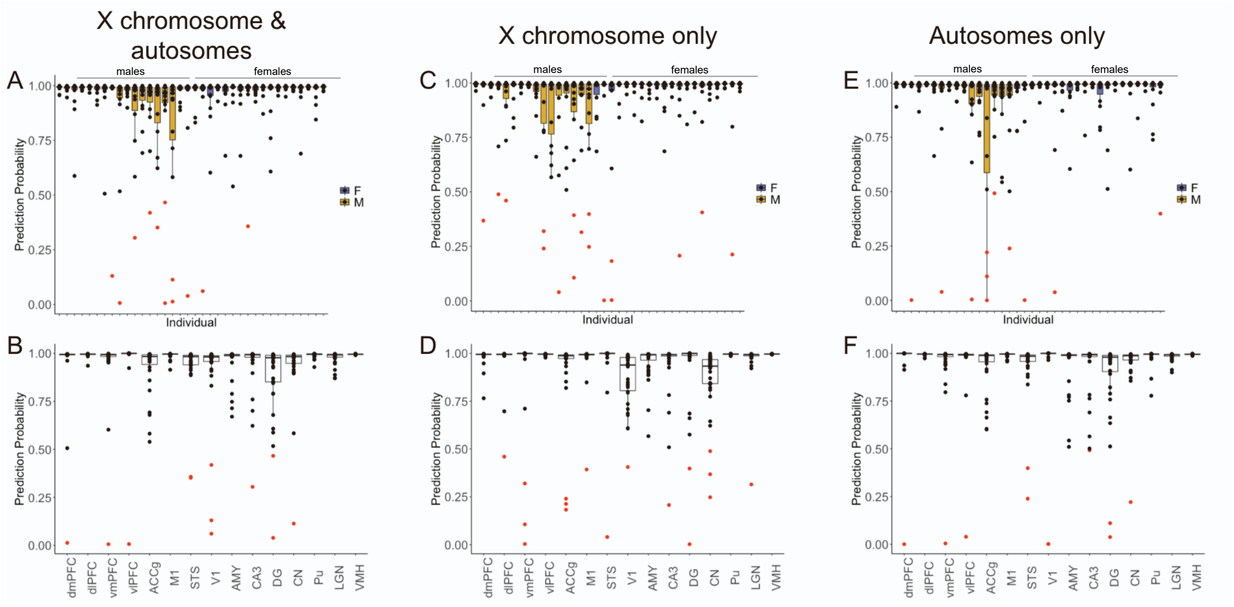

Figure S11 | Per sample sex prediction probabilities, related to Figure 6A.

A-F) Boxplots of prediction probabilities of the known sex per individual (top row) and region (bottom row). Dots indicate values for individual samples. Purple boxes = female, yellow boxes = male, black dots = correctly classified samples, red dots = incorrectly classified sample (prediction probability of correct sex < 0.5). Results are shown separately for models run incorporating all X chromosome and autosomal genes (A,B), X chromosome genes only (C,D), and autosomal genes only (E,F).

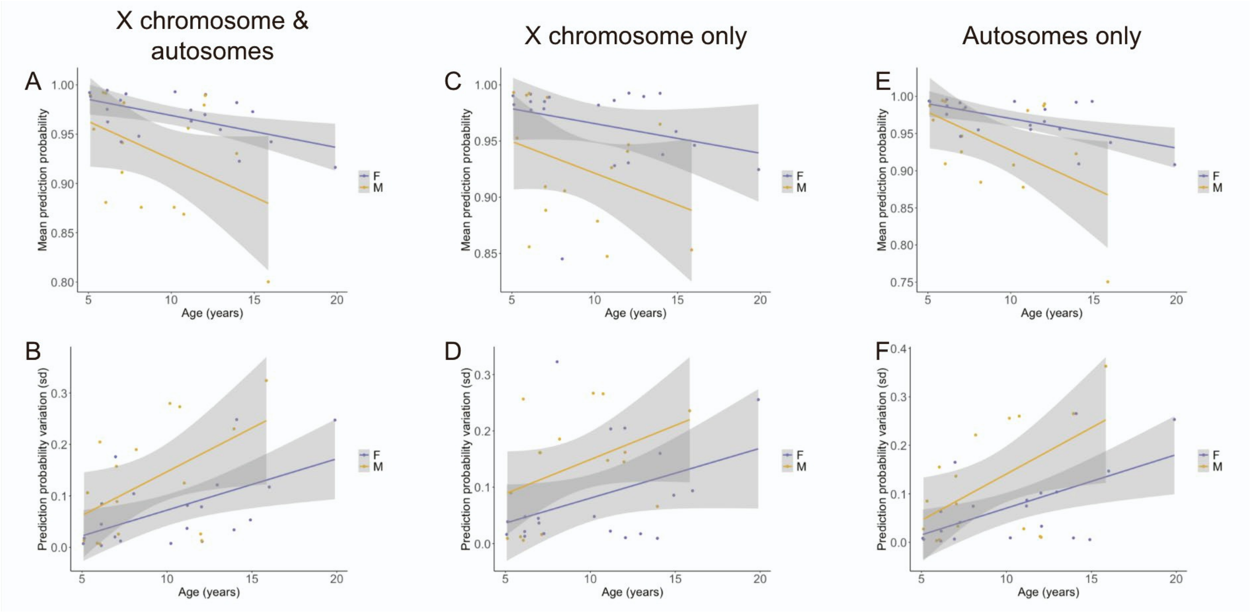

Figure S12 | Sex prediction probability (mean and sd) as a function of sex, related to Figure 6B.

A) Average prediction probability of known sex as a function of age (years) for females (purple) and males (yellow) or models run incorporating all X chromosome and autosomal genes (all: estimate = -0.003750, p = 0.0606; females: estimate = -0.003259, p = 0.00798; males: estimate = -0.007654, p = 0.0893)

B) Average standard deviation of prediction probability of known sex as a function of age (years) for females (purple) and males (yellow) or models run incorporating all X chromosome and autosomal genes (all: estimate = 0.010668, p = 0.0089; females: estimate = 0.009981, p = 0.0115; males: estimate = 0.01698, p = 0.0426)

C) Average prediction probability of known sex as a function of age (years) for females (purple) and males (yellow) or models run incorporating X chromosome genes only (all: estimate = -0.002656, p = 0.211; females: estimate = -0.002640, p = 0.202; males: estimate = -0.005653, p = 0.17)

D) Average standard deviation of prediction probability of known sex as a function of age (years) for females (purple) and males (yellow) or models run incorporating X chromosome genes only (all: estimate = 0.008390, p = 0.0469; females: estimate = 0.008853, p = 0.0853; males: estimate = 0.012116, p = 0.0983)

E) Average prediction probability of known sex as a function of age (years) for females (purple) and males (yellow) or models run incorporating autosomal genes only (all: estimate = -0.005151, p = 0.0158; females: estimate = -0.003971, p = 0.00482; males: estimate = -0.010242, p = 0.0356)

F) Average standard deviation of prediction probability of known sex as a function of age (years) for females (purple) and males (yellow) or models run incorporating autosomal genes only (all: estimate = 0.012182, p = 0.00411; females: estimate = 0.011033, p = 0.00782; males: estimate = 0.019074, p = 0.0315)

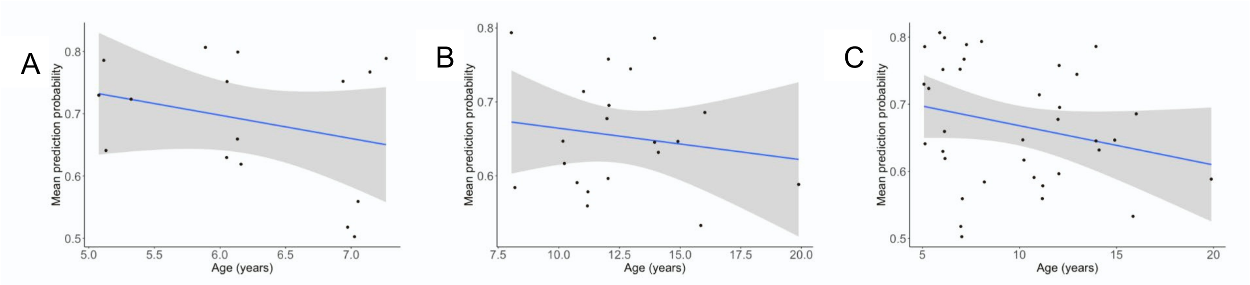

Figure S13 | Sex prediction probability (mean and standard deviation) as a function of sex, related to Figure 6B.

A) Prediction probability per individual (<=8 years group), averaged across regions and simulations (estimate = -0.037, p = 0.282).

B) Prediction probability per individual (>8 years group), averaged across regions and simulations (estimate = -0.004, p = 0.509).

C) Prediction probability per individual (combined results from separate analyses of both age groups), averaged across regions and simulations (estimate = -0.006, p = 0.139).

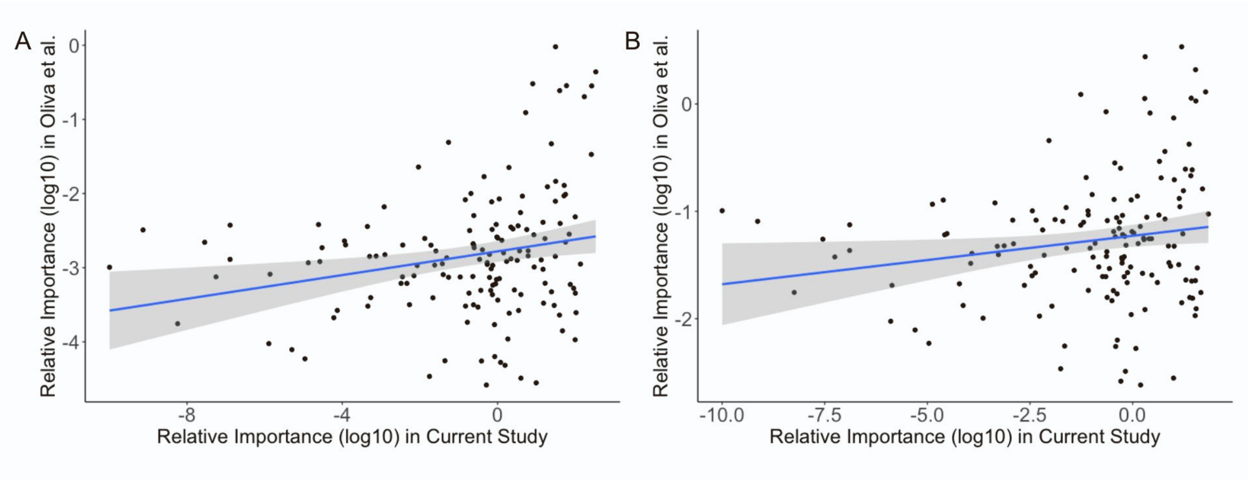

Figure S14 | Relative importance of X chromosome genes for sex prediction in macaques vs. humans, related to Figure 6D.

A) Relevance is summed across regions in both studies (ρ = 0.222, p = 0.006).

B) Relevance is averaged across regions in both studies (ρ = 0.0170, p = 0.038).

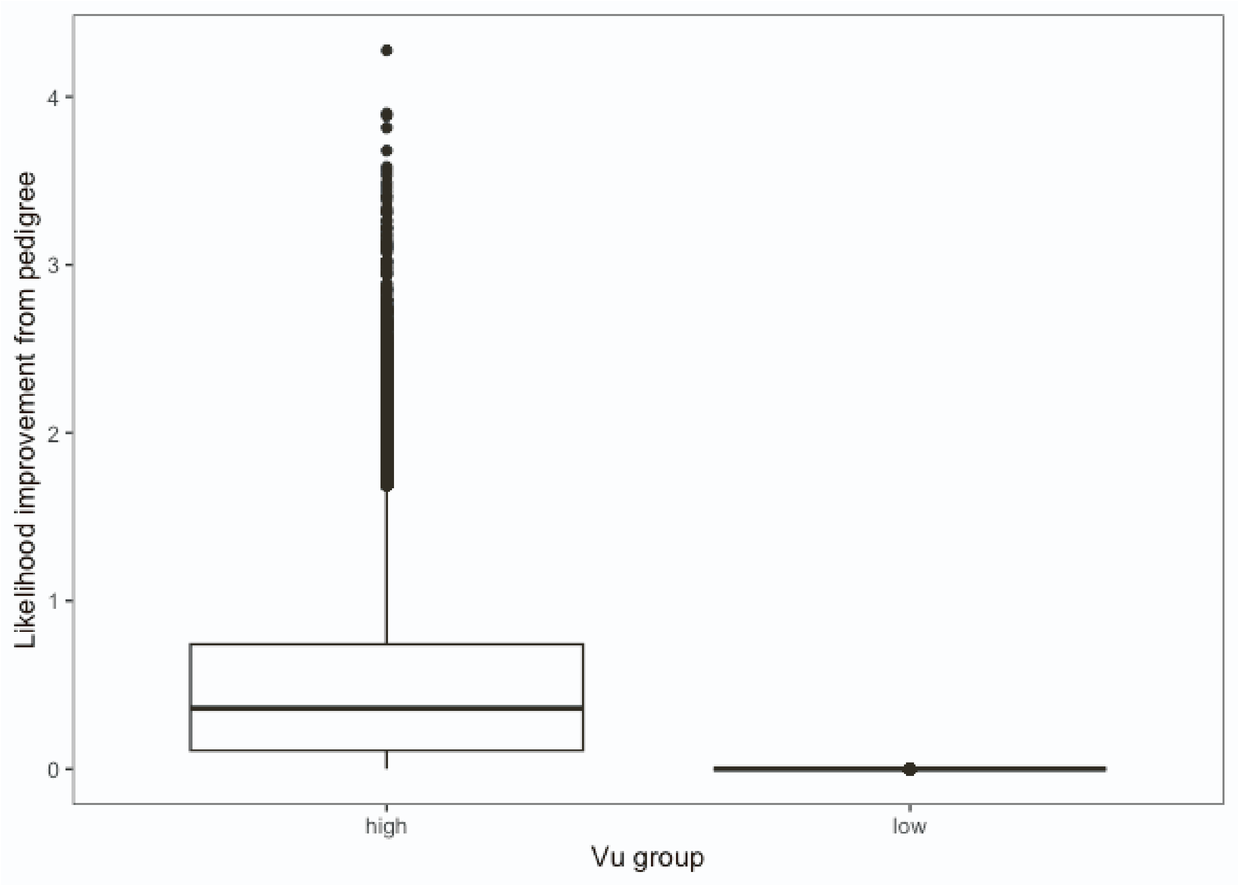

Figure S15 | Difference in likelihood between models that include the pedigree and those that do not, related to Figure 7B (see Methods).

“high *Vu*” group = log(*Vu*) > -15; “low *Vu*” group = log(*Vu*) < -15

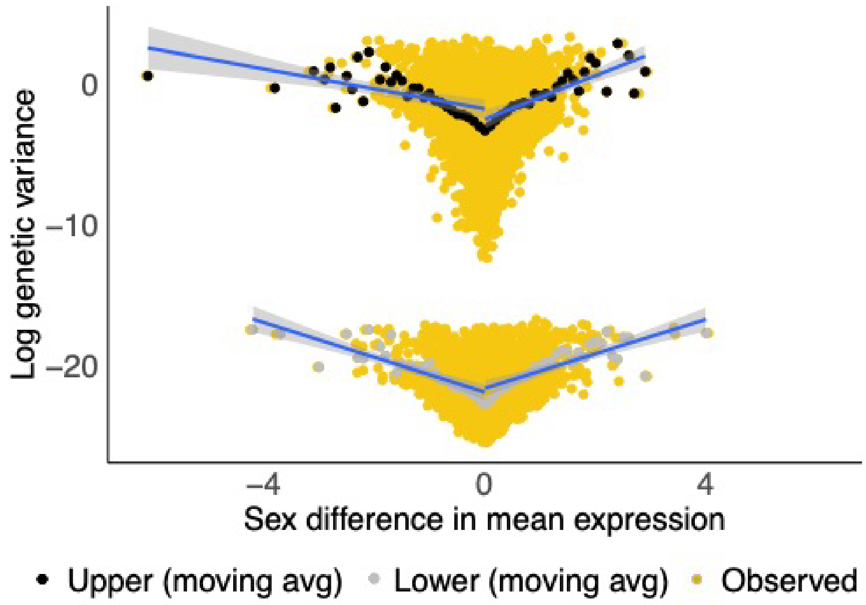

Figure S16 | Genetic variance as a function of sex-biased expression, related to Figure 7B.

Figure 4C with directional (instead of absolute) sex-bias (x-axis). Genetic variance (log) as a function of the difference in mean residual expression per gene and region (male-bias and upper *Vu*: $\rho = 0.286$; female-bias and upper *Vu*: -0.248; male-bias and lower *Vu*: $\rho = 0.\ 315$; female-bias and lower *Vu*: -0. 290; all p-values < 2.2e-16).

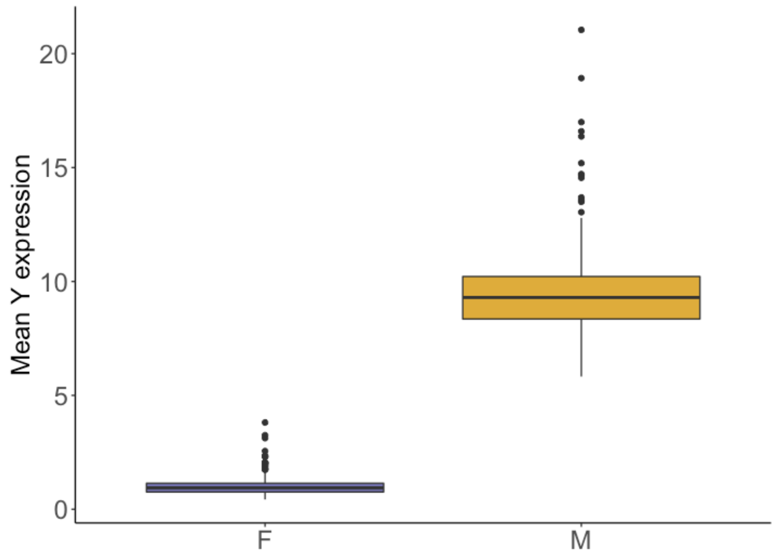

Figure S17 | Confirming chromosomal sex of samples using Y chromosome gene expression (see Methods).

Mean expression (TPM) of all Y genes for female and male samples. Reads were mapped to the original (non-sex specific) transcriptome. The female reads detected here represent the gametologues (X-Y homologues) and are not present in the sex-specific mapped data used in the manuscript. Boxplots indicate the median (black horizontal line), first and third quartiles (i.e., interquartile range, IQR; lower and upper hinges), and ranges extending from each to 1.5 x IQR beyond each hinge (whiskers). Points represent individual genes that are outliers (i.e., beyond whiskers).

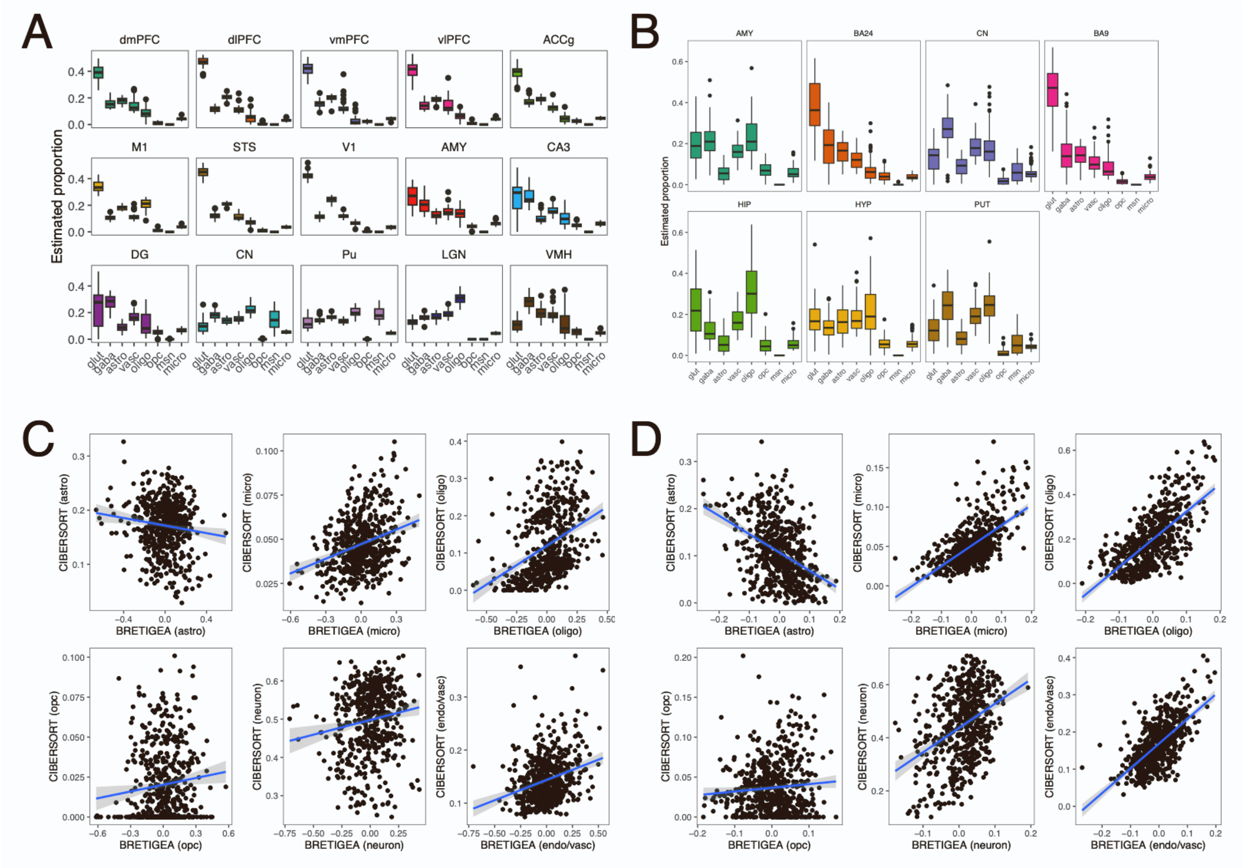

Figure S18 | Estimated cell type proportions across two methods, related to Figure 4A-C (see Methods).

A) CIBERSORT proportions in macaques for each region.

B) CIBERSORT proportions in humans for each region.

C) Correlations between CIBERSORT cell type proportions and BRETIGEA surrogate proportion variables across all macaque samples (astro = astrocytes, ρ = -0.070, p = 0.106; micro = microglia, ρ = 0.251, p = 5.728e-09; oligo = oligodendrocytes, ρ = 0.455, p = < 2.2e-16; opc = OPCs, 0.135, p = 0.002; neuron = neurons in BRETIGEA and glutaminergic + GABAergic neurons in CIBERSORT, ρ = 0.166, p < 0.001; endo/vasc = endothelial in BRETIGEA and vascular in CIBERSORT, ρ = 0.280, p < 7.216e-11).

D) Correlations between CIBERSORT cell type proportions and BRETIGEA surrogate proportion variables across all human samples (astro = astrocytes, ρ = -0.399, p = < 2.2e-16; micro = microglia, ρ = 0.632, p < 2.2e-16; oligo = oligodendrocytes, ρ = 0.656 p = < 2.2e-16; opc = OPCs, ρ = 0.020, p = 0.641; neuron = neurons in BRETIGEA and glutaminergic + GABAergic neurons in CIBERSORT, ρ = 0.327, p = 1.982e-15; endo/vasc = endothelial in BRETIGEA and vascular in CIBERSORT, ρ = 0.626, p < 2.2e-16).

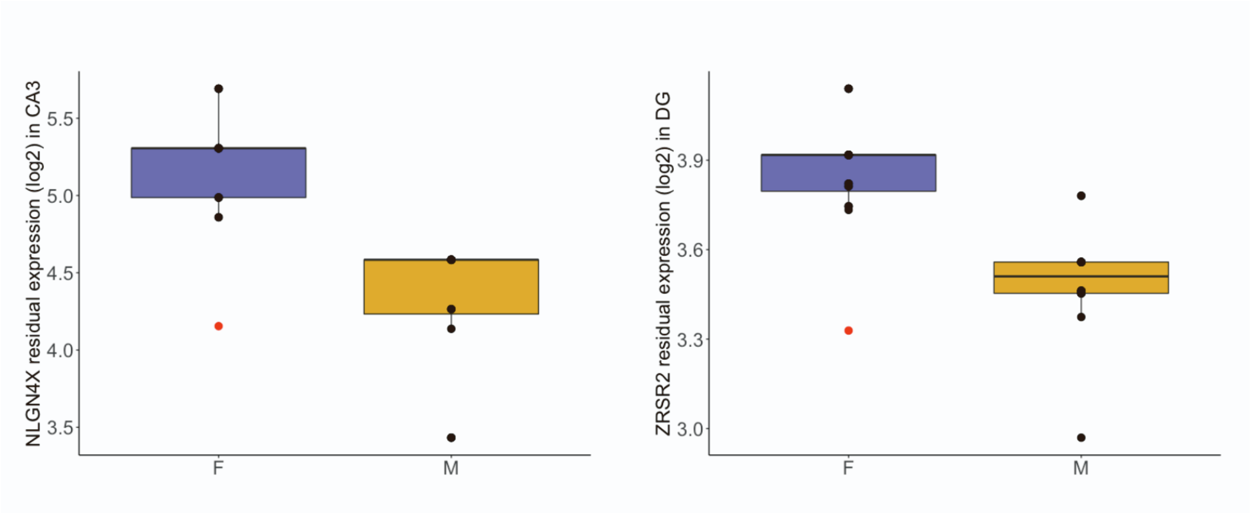

Figure S19 | Interindividual variation in expression of sex-predictive genes, related to Figure 6A (see Methods).

Boxplots of the residual expression values for the most influential gene in a given region from X chromosome gene models (left: *NLGN4X* in CA3; right: *ZRSR2* in DG). The red point represents the misclassified female sample. In humans, *ZRSR2* escapes and *NLGN4X* variably escapes XCI. Boxplots indicate the median (black horizontal line), first and third quartiles (i.e., interquartile range, IQR; lower and upper hinges), and ranges extending from each to 1.5 x IQR beyond each hinge (whiskers). Points represent individual genes that are outliers (i.e., beyond whiskers).
